# The USP46 deubiquitylase complex increases Wingless/Wnt signaling strength by stabilizing Arrow/LRP6

**DOI:** 10.1038/s41467-023-41843-0

**Published:** 2023-10-05

**Authors:** Zachary T. Spencer, Victoria H. Ng, Hassina Benchabane, Ghalia Saad Siddiqui, Deepesh Duwadi, Ben Maines, Jamal M. Bryant, Anna Schwarzkopf, Kai Yuan, Sara N. Kassel, Anant Mishra, Ashley Pimentel, Andres M. Lebensohn, Rajat Rohatgi, Scott A. Gerber, David J. Robbins, Ethan Lee, Yashi Ahmed

**Affiliations:** 1 Department of Molecular and Systems Biology and the Dartmouth Cancer Center, Geisel School of Medicine, Dartmouth College, Hanover, NH 03755 USA; 2 Department of Cell and Developmental Biology, Vanderbilt University, Nashville, TN 37232 USA; 3 Laboratory of Cellular and Molecular Biology, Center for Cancer Research, National Cancer Institute, National Institutes of Health, Bethesda, MD 20892 USA; 4 Department of Biochemistry, Stanford University School of Medicine, Stanford, CA 94305 USA; 5 Department of Molecular and Systems Biology and the Dartmouth Cancer Center, Geisel School of Medicine at Dartmouth, Lebanon, NH 03766 USA; 6 Department of Oncology, Lombardi Comprehensive Cancer Center, Georgetown University, Washington, DC 20057 USA

**Keywords:** Morphogen signalling, Cell signalling, Pattern formation

## Abstract

The control of Wnt receptor abundance is critical for animal development and to prevent tumorigenesis, but the mechanisms that mediate receptor stabilization remain uncertain. We demonstrate that stabilization of the essential Wingless/Wnt receptor Arrow/LRP6 by the evolutionarily conserved Usp46-Uaf1-Wdr20 deubiquitylase complex controls signaling strength in *Drosophila*. By reducing Arrow ubiquitylation and turnover, the Usp46 complex increases cell surface levels of Arrow and enhances the sensitivity of target cells to stimulation by the Wingless morphogen, thereby increasing the amplitude and spatial range of signaling responses. Usp46 inactivation in Wingless-responding cells destabilizes Arrow, reduces cytoplasmic accumulation of the transcriptional coactivator Armadillo/β-catenin, and attenuates or abolishes Wingless target gene activation, which prevents the concentration-dependent regulation of signaling strength. Consequently, Wingless-dependent developmental patterning and tissue homeostasis are disrupted. These results reveal an evolutionarily conserved mechanism that mediates Wnt/Wingless receptor stabilization and underlies the precise activation of signaling throughout the spatial range of the morphogen gradient.

## Abstract (web summary)

Wnt receptor levels drive signaling. Here the authors find that precise signaling in the Wnt morphogen gradient relies on Usp46, a deubiquitylase that enhances target cells sensitivity by increasing cell surface levels of the Wnt receptor Arrow/LRP6.

## Introduction

Morphogens are secreted ligands that spread from localized sources of synthesis to direct growth and patterning during animal development, maintain tissue homeostasis during adulthood, and promote regeneration following injury. The evolutionarily conserved Wnt/Wingless family of ligands, which are associated with numerous developmental disorders and cancers^1^, have provided a paradigm for morphogen action. Pioneering studies in *Drosophila* revealed that Wingless/Wnt forms an extracellular concentration gradient that directly activates signaling at long range in the developing wing, leg, and eye^2,3^. This long-range, concentration-dependent action of Wingless/Wnt was subsequently found essential for organogenesis and tissue homeostasis in other physiological contexts^2–11^. Studies on the formation of Wnt signaling gradients have focused largely on how Wnt is released from producing cells and delivered to target cells^8,12–15^. In contrast, the mechanisms in target cells that ensure precision in the amplitude and spatial range of signaling responses within the gradient are not well-understood.

Among the top hits in genome-wide insertional mutagenesis screens for positive regulators of Wnt signaling in human cells^16^, we identified an evolutionarily conserved WD40 repeat (WDR) containing protein, WDR20^17^. WDR20 is an obligate stimulatory subunit for two deubiquitylating enzymes, Ubiquitin-specific protease 12 (USP12) and USP46^17–20^, paralogs that share 88% amino acid identity^21^. The activity of USP12 and USP46 is facilitated by WDR20 and another stimulatory WDR protein, USP1-associated factor (UAF1)/WDR48^17,22,23^. WDR20 and UAF1 potentiate the activity of USP12 and USP46 by allosterically increasing their catalytic efficiency without increasing their substrate-binding affinity^24–26^. Some substrates are shared by both USP12 and USP46^27,28^, whereas others are targeted exclusively by only one of the paralogs^18–21,27,29–32^.

Here, we investigated the roles of the *Drosophila* USP46, UAF1, and WDR20 orthologs in the reception of the Wingless/Wnt morphogen. By reducing Arrow/LRP6 ubiquitylation, the Usp46 complex increases Arrow/LRP6 stability and cell surface levels, thereby enhancing the sensitivity of target cells to Wingless stimulation. Consequently, the Usp46 complex regulates Wingless-dependent target gene activation, cell fate specification, and tissue homeostasis in two well-characterized physiological contexts: the developing wing and the adult intestine. Inactivation of the Usp46 complex destabilizes Arrow/LRP6 in cultured cells and in vivo, which diminishes the cytoplasmic accumulation of the transcriptional activator Armadillo/β-catenin following Wingless exposure. As a result, the strength of signaling responses is decreased cell-autonomously, with reductions in both amplitude and spatial range. These findings indicate that Arrow/LRP6 stabilization mediated by Usp46-dependent deubiquitylation is required for concentration-dependent responses in target cells, providing precision in signaling throughout the spatial range of the Wingless morphogen gradient.

## Results

### Usp46, Wdr20, and Uaf1 are positive regulators of Wingless signaling in vivo

To determine whether the USP46-UAF1-WDR20 complex regulates Wnt signaling in physiological contexts, we took a loss-of-function approach in *Drosophila*. The closest relative of the human USP46 and USP12 paralogs is encoded by a single *Drosophila melanogaster* gene, *Usp12-46* (*CG7023*, herein *Usp46*). Similarly, single genes encode the WDR20 (*CG6420*, herein *Wdr20*) and UAF1 (*CG9062*, herein *Uaf1*) orthologs in *Drosophila*. Alignment of the human and *Drosophila* orthologs of USP46, WDR20, and UAF1 revealed a high degree of primary sequence conservation across all known domains (Fig. S1). Specifically, the *Drosophila* and human orthologs share 63% amino acid similarity in the USP peptidase domain in USP46, between 50% and 86% similarity in the WD40 repeats in WDR20, and between 70% and 95% similarity in the WD40 repeats in UAF1.

**Fig. 1:**
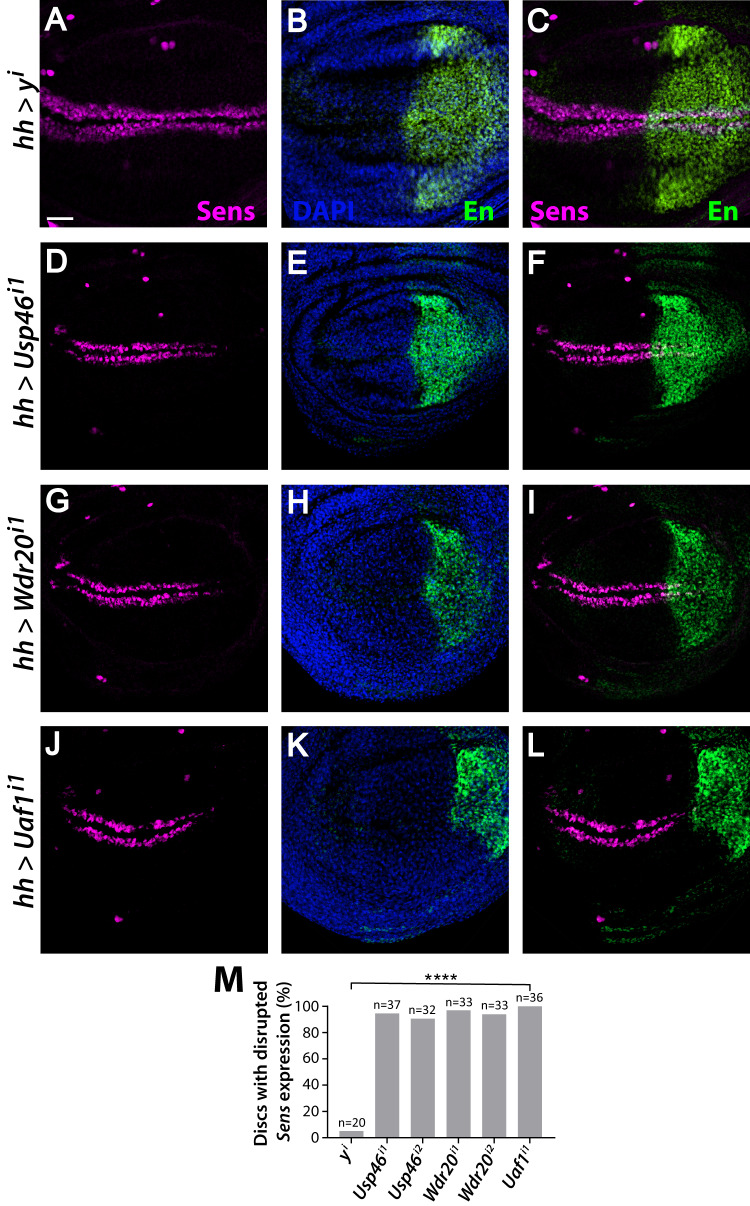
The Usp46 complex promotes expression of the Wingless target gene *senseless* (*sens*) in the larval wing disc. **A**–**L** RNAi constructs targeting each Usp46 complex component or the *yellow* negative control were expressed in the posterior compartment (marked by Engrailed (En, green)) of the third instar larval wing discs using the *hedgehog (hh)-Gal4* driver. Senseless (Sens, magenta). DAPI (blue) marks nuclei. Scale bar (**A**–**L**): 20 µM. Dorsal, top and posterior, right. **A**–**C** *hh-Gal4*-driven expression of a control RNAi construct targeting the *yellow (y)* gene. No loss of Sens was observed. **D**–**L** *hh-Gal4-*driven expression of RNAi constructs targeting *Usp46* (**D**, **F**), *Wdr20* (**G**, **I**) and *Uaf1* (**J**, **L**) results in decreased Sens in the posterior compartment. Only one RNAi line targeting *Uaf1* was available. **M** Quantification is shown as percentage of discs of each genotype with decreased Sens. N is the number of discs analyzed. ****p < 0.0001 (0 for all genotypes, one-tailed t-test). Source data are provided in the Source Data file.

To evaluate the role of the Usp46 complex in Wingless signaling, we investigated a well-characterized model: the third instar larval wing imaginal disc, the precursor of the adult wing^2,3,11^. The Wingless concentration is highest at the dorsoventral (D-V) boundary of the wing disc and there specifies the fate of cells that will form sensory bristles at the adult wing margin^2,3^, in part through expression of the Wingless target gene *senseless (sens*)^33,34^. RNAi-mediated knockdown of *Usp46* in the posterior compartment of the wing disc using the *hedgehog* (*hh)-Gal4* driver decreased Sens levels solely in the posterior compartment (marked by Engrailed) in >90% of discs (Fig. 1D–F, M). RNAi-mediated depletion of *Wdr20* and *Uaf1* had comparable effects (Fig. 1G–M). Multiple independent RNAi constructs that target different regions of *Usp46* or *Wdr20* reduced Sens specifically in the posterior compartment, indicating a robust effect (Fig. S2A–F, Fig. 1M). In contrast, RNAi-mediated knockdown of a control gene, *yellow (y)*, resulted in nearly no Sens reduction (Fig. 1A–C, M), supporting the specificity of the Usp46 complex RNAi results. Furthermore, depletion of *Usp46, Wdr20* or *Uaf1* did not inhibit *wingless* expression, indicating that the observed reduction in Sens was not due to decreased Wingless levels, but instead resulted from impaired reception of the Wingless ligand or downstream defects in the Wingless signaling pathway (Fig. S3). As *wingless* is a target gene of Notch signaling at the D-V boundary^35^, these results also indicate that the Usp46 complex does not regulate Notch in this context, in contrast with a previous analysis of thoracic bristle sockets^32^. These findings indicate that Usp46, and its allosteric regulators Uaf1 and Wdr20, promote Wingless signaling in target cells in the wing imaginal disc.

We further characterized *Usp46* mutant cells in the wing disc that were generated by combining CRISPR-based mutagenesis with tissue-specific expression of *Cas9* and *Usp46* guide RNAs. To identify *Usp46* mutant cells, we added a C-terminal V5 epitope tag to endogenous Usp46 using CRISPR-induced homology-directed repair^36^. Usp46-V5 immunostaining revealed that Usp46 is present at invariant levels throughout the wing disc (Fig. S4A). Confirming the specificity of the Usp46-V5 signal, RNAi-mediated knockdown of *Usp46* in the dorsal compartment of the wing disc using the *apterous (ap)-Gal4* driver resulted in a nearly complete loss of V5 staining in the dorsal compartment (Fig. S4D–F). We then used the UAS/Gal4 system^37^ to express *Usp46* single guide RNAs (sgRNAs) for CRISPR-based mutagenesis^38^. Concomitant expression of *Cas9* and *Usp46* sgRNAs in the posterior compartment using *hh-Gal4* reduced Usp46 levels in the posterior compartment, as revealed by loss of the Usp46-V5 signal (Fig. S5D, F). The compartment-specific reduction of Usp46 resulted in a compartment-specific decrease in Sens (Fig. S5E, F). Together, these findings support the conclusion that Usp46 is a positive regulator of Wingless signaling.

To rule out potential off-target effects, we expressed other sgRNAs that target different parts of the *Usp46* coding region using *hh-Gal4*. Co-expression of *Cas9* with these independent *Usp46* sgRNAs also reduced Sens solely in the posterior compartment (Fig. S6E, F). In contrast, the *hh-Gal4*-driven expression of Cas9 in the posterior compartment in the absence of *Usp46* sgRNAs resulted in no observed Sens loss (Fig. S6B, C), ruling out the possibility that these findings were caused by Cas9-induced cell death^39^. As an additional control for specificity, we examined *hh-Gal4*-driven expression of *ebony (e)* sgRNAs, which resulted in a characteristic dark cuticle phenotype solely in the posterior compartment of the wing (Fig. S6N), but no reduction in Sens levels (Fig. S5A–C). Together, these findings provide additional evidence that Usp46 promotes Wingless signaling in the larval wing disc.

Inactivation of Wdr20 and Uaf1 using the same approach also decreased Wingless signaling in the wing disc. *hh-Gal4*-driven expression of *Cas9* and *Wdr20* or *Uaf1* sgRNAs reduced levels of Wdr20 (Fig. S5G, I) or Uaf1 (Fig. S5J, L), respectively, and concomitantly reduced Sens levels solely in the posterior compartment (Fig. S5H, K). sgRNAs that target different parts of the *Wdr20* or the *Uaf1* coding regions also reduced Sens (Fig. S6H, K**)**. Furthermore, *Usp46*, *Wdr20*, or *Uaf1* sgRNAs did not reduce *wingless* expression (Fig. S7), confirming that the reduction in Sens resulted from attenuation of Wingless pathway activity, rather than decreased Wingless levels. We conclude that all three components of the Usp46 complex are necessary for activating Wingless signaling in the developing wing.

### The Usp46 complex increases the amplitude and spatial range of signaling induced by the Wingless morphogen

To determine the extent to which the Usp46 complex modulates signaling in target cells responding to the Wingless morphogen, and whether this occurs cell-autonomously, we isolated *Usp46*, *Wdr20*, and *Uaf1* null alleles. We used CRISPR-based mutagenesis^38^ to isolate alleles with either large deletions in the coding region or frameshift mutations resulting in premature stop codons that encode truncated proteins lacking most of the evolutionarily conserved domains (Fig. S8). *Usp46*, *Wdr20*, and *Uaf1* null mutants were viable but displayed reduction in lifespan (Fig. S9), severe defects in the adult intestinal epithelium (see below), and sterility. We therefore were unable to evaluate *Usp46* mutant embryos in which both maternal and zygotic contributions were depleted.

To further test the role of the Usp46 complex in Wingless-receiving cells, we examined the adult intestine, an excellent model for evaluating Wingless signaling gradients^40^. The expression of Wingless target genes, such as *frizzled 3* (*fz3)* and *naked (nkd)*, is high near the Wingless source at intestinal compartment boundaries and decreases as a function of distance from these boundaries, reflecting the decreasing Wingless concentration (Fig. S10A–C)^9,40–42^. To confirm the specificity of the *fz3-GFP* transcriptional reporter at the boundary between the midgut and hindgut (midgut-hindgut boundary, MHB), we used MARCM (mosaic analysis with a repressible cell marker)^43^ to generate and mark homozygous null mutant clones of the Wingless receptor *arrow* with dsRed. Complete loss of *fz3-GFP* expression was observed in all *arrow* mutant clones examined (Fig. S10D–I). In contrast, clones of wild-type cells displayed no reduction in *fz3-GFP* (Fig. S10J–O).

**Fig. 2:**
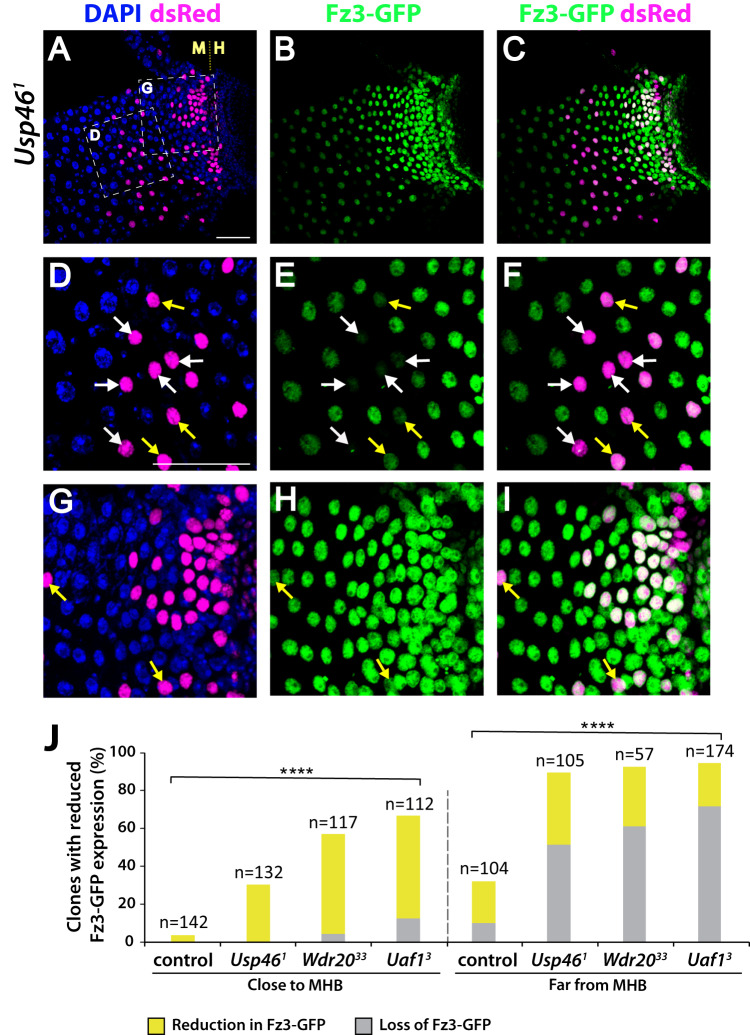
Usp46 promotes expression of the Wingless target gene *fz3* in the posterior midgut. **A**–**C** *Usp46*^*1*^ null mutant clones (magenta) and *frizzled3-GFP* (*fz3-GFP)* expression (green) in the adult intestinal epithelium of the posterior midgut. The midgut-hindgut boundary (MHB) is delineated (M | H). DAPI (blue) marks nuclei. Posterior, right. **D**–**F** Higher magnification view of box D in panel A showing a region distant from the MHB. *Usp46*^*1*^ null mutant cells in this region showed complete loss of *fz3-GFP* (white arrows), whereas those slightly closer to the MHB showed a nearly complete reduction in *fz3-GFP* (yellow arrows). **G**–**I** Higher magnification view of box G in panel A showing a region near the MHB. *Usp46*^*1*^ null mutant cells in this region display either a partial decrease (yellow arrows) or no decrease in *fz3-GFP*. Scale bars (**A**–**C**) and (**D**–**I**): 50 µM. **J** Quantification is shown as percentage of clones of each genotype with decreased *fz3-GFP* (yellow) or the absence of *fz3-GFP* (gray) expression. Clones (n=number) close and far from the MHB were analyzed. ****p < 0.0001 (0 for all genotypes, one-tailed *t*-test). Source data are provided in the Source Data file.

To test the role of Usp46 in the Wingless signaling gradient, we examined the effects of Usp46 loss on Wingless target gene activation. In *Usp46* null mutant clones located ~8– 20 cell lengths from the MHB (Fig. 2A–C, magenta), *fz3-GFP* expression was completely lost (Fig. 2D–F, white arrows and Fig. 2J) or severely reduced (Fig. 2D–F, yellow arrows and Fig. 2J) in a cell autonomous manner, whereas in *Usp46* mutant clones located one to seven cell lengths from the MHB, *fz3-GFP* expression was partially reduced (Fig. 2G–I, yellow arrows, and Fig. 2J), or exhibited no apparent change. We repeated this experiment with a different *Usp46* null allele and observed the same defects (Fig. S11). These results reveal that a cell autonomous role of Usp46 in Wingless-receiving cells is critical for increasing both the amplitude and spatial range of responses to Wingless stimulation and thus for precision in the signaling gradient.

Wdr20 and Uaf1 inactivation similarly resulted in decreased Wingless signaling in the posterior midgut. *fz3-GFP* expression was entirely or nearly completely lost in a cell autonomous manner in *Wdr20* mutant clones (Fig. S12D–F, white and yellow arrows) and *Uaf1* mutant clones (Fig. S13D–F, white and yellow arrows) located ~8–20 cell lengths from the MHB, whereas in *Wdr20* mutant clones or *Uaf1* mutant clones located 1 to 7 cell lengths from the MHB there was a partial reduction (Fig. S12G–I and S13G–I, yellow arrows) or no apparent change. These results were confirmed with additional *Wdr20* and *Uaf1* null alleles (Fig. S14 and Fig. S15). These findings indicate that all three components of the Usp46 complex are essential for increasing the amplitude and spatial range of signaling responses within the Wingless morphogen gradient.

**Fig. 3:**
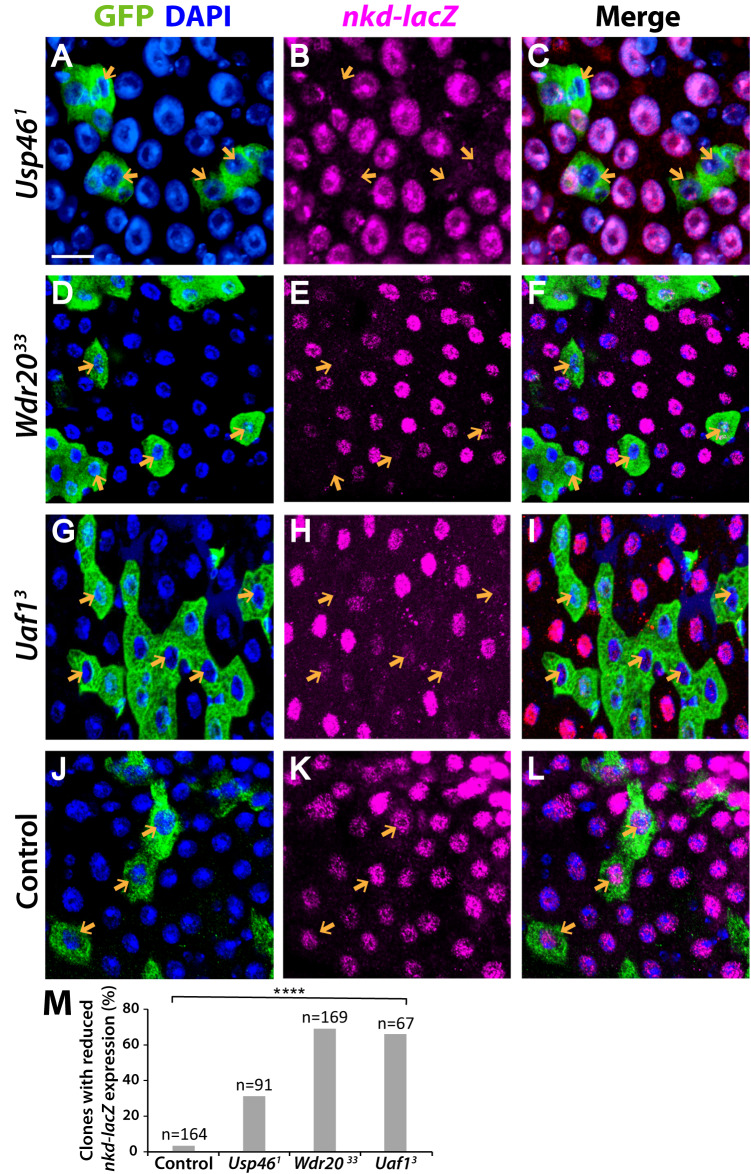
The Usp46 complex is required for expression of the Wingless target gene *naked* in the posterior midgut. **A**–**I** Null mutant MARCM clones (green) of *Usp46* (**A**, **C**), *Wdr20* (**D**, **F**), and *Uaf1* (**G**, **I**) in the posterior midgut resulted in decreased expression of *nkd-lacZ* (magenta) in a cell-autonomous manner (orange arrows). For inactivation of all three Usp46 components, the largest reduction in *nkd-lacZ* was displayed in mutant clones far from the MHB. DAPI (blue) marks nuclei. **J**–**L** *nkd-lacZ* does not decrease in wild-type (*FRT82B*) clones. Scale bar (**A**, **L**): 20 µM. **M** Quantification is shown as percentage of clones of each genotype with decreased *nkd-lacZ* expression. N is the number of clones analyzed. ****p < 0.0001 (0 for all genotypes, one-tailed t-test). Source data are provided in the Source Data file.

To determine whether the Usp46 complex is required for the activation of other Wingless target genes within the gradient, we investigated the regulation of *nkd*, using the transcriptional reporter *nkd-lacZ*^44^. Similar to that of *fz3-GFP*, the level of *nkd-lacZ* is highest at the MHB and decreases with distance from the MHB^42,45^. Inactivation of any of the three Usp46 complex components in the posterior midgut reduced the levels of *nkd-lacZ* in a cell-autonomous manner (Fig. 3A–I). For all three Usp46 components, we observed results that were qualitatively similar to those observed with *fz3-GFP*: in mutant clones located at a distance from the MHB, *nkd-lacZ* expression was completely lost, whereas in mutant clones closer to the MHB, *nkd-lacZ* expression was either partially reduced in comparison with the neighboring wild-type cells or displayed no apparent change. These findings further support the conclusion that by increasing the amplitude and spatial range of signaling responses, the Usp46 complex facilitates the precise, concentration-dependent regulation of Wingless target genes within the morphogen gradient.

### The Usp46 complex is required for the Wingless-dependent regulation of intestinal stem cell proliferation

**Fig. 4:**
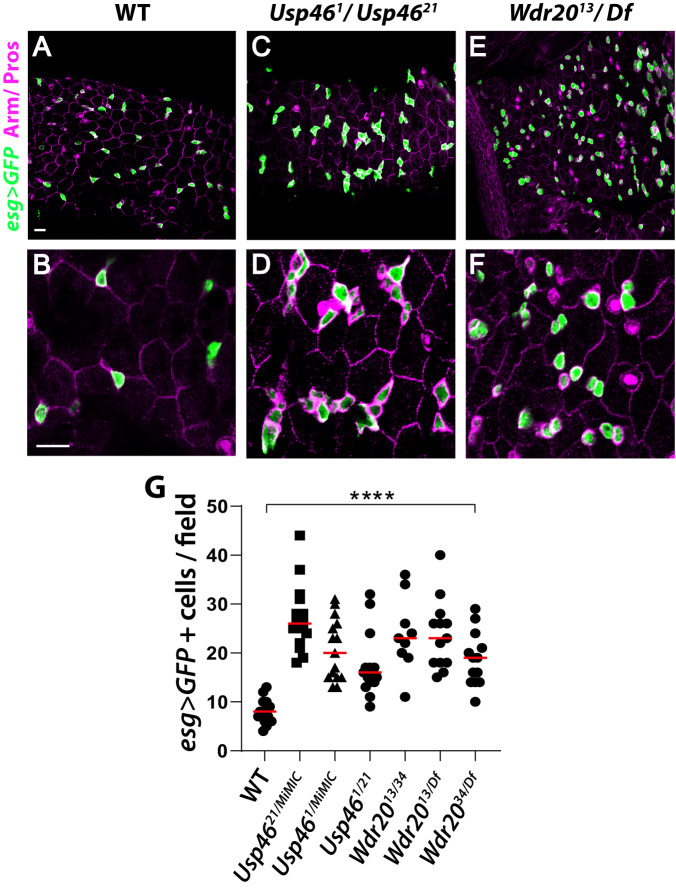
Usp46 and Wdr20 regulate intestinal stem cell proliferation in the adult midgut. **A**, **B** Wild-type adult posterior midgut epithelium stained for *escargot (esg)>GFP* (green) to mark intestinal stem and progenitor cells, Prospero (Pros, magenta) to mark enteroendocrine cells, and Armadillo (Arm, magenta) to mark the plasma membrane of all epithelial cells. Panel B is a higher magnification view of A. **C–F** Posterior midgut from transheterozygote null mutants of *Usp46* (**C**, **D**) or *Wdr20* (**E**, **F**) are shown. Overproliferation of adult midgut epithelial cells, revealed by *esg* > *GFP* (green), is observed following inactivation of *Usp46* or *Wdr20*. Panels D and F are higher magnification views of C and E, respectively. Armadillo (Arm, magenta), Prospero (Pros, magenta). *Df* refers to a chromosomal deficiency that eliminates *Wdr20* (see Methods). Scale bars (**A**, **C**, **E**) and (**B**, **D**, **F**): 20 µM. **G** Quantification of *esg* > *GFP* positive cells is shown as mean (red line). Each point represents an individual posterior midgut (*n* = 15 for WT, *n* = 15 for *Usp46*^*21/MiMIC*^, *n* = 15 for *Usp46*^*1/MiMIC*^, *n* = 15 for *Usp46*^*1/21*^, *n* = 9 for *Wdr20*^*13/34*^, *n* = 13 for *Wdr20*^*13/Df*^, *n* = 13 for *Wdr20*^*34/Df*^). A 0.051mm^2^ field was measured in the R5 region of each posterior midgut. ****p < 0.0001 (p values in order:  < 1E-8, 7E-8, 1.5E-5, 1.5E-7, 2E-8 and 3.6E-7, two-tailed t-test). *Usp46*^*MiMIC*^ contains an insertion of a *Minos*-mediated integration cassette in the *Usp46* gene (see Methods). Source data are provided in the Source Data file.

Wingless signaling was initially proposed to promote adult intestinal stem cell (ISC) self-renewal and proliferation during homeostasis^46^, though this conclusion was later challenged^40,47,48^. Subsequently, Wingless pathway activation was found essential for the non-autonomous control of ISC proliferation by enterocytes during homeostasis^42^. Attenuation of Wingless signaling in the adult posterior midgut results in increased numbers of ISCs and ISC-derived progenitor cells termed enteroblasts (EB)^9,42^. We therefore investigated whether Usp46 promotes this Wingless-dependent control of intestinal homeostasis. We found that in *Usp46* null mutants, stem and progenitor cell number is aberrantly increased by comparison to wild-type, as revealed by the stem/progenitor cell marker *escargot* > *GFP* (*esg* > *GFP*) (Fig. 4A–D, G). Similar to *Usp46* inactivation, *Wdr20* inactivation also resulted in an increased number of intestinal stem and progenitor cells (Fig. 4E–G). Independently-derived *Usp46* and *Wdr20* null alleles confirmed these results (Fig. 4G). *Uaf1* was not tested with this assay due to technical constraints resulting from its genomic location. These findings demonstrate that the Usp46 complex is necessary to restrict stem and progenitor cell number in the adult midgut, a known function of Wingless signaling.

Inactivation of Wingless signaling is known to increase ISC proliferation without disrupting the asymmetric ISCs division that specifies enteroblasts (EBs)^49^. To determine whether inactivation of the Usp46 complex has similar effects, we examined an EB-specific marker, *Suppressor of Hairless (Su(H)-lacZ)*. Inactivation of *Usp46*, *Wdr20, or Uaf1* resulted in an aberrant increase in the number of EBs by comparison to wild-type (Fig. S16A). Thus, the loss of Usp46 complex components did not block the asymmetric division of ISCs. We conclude that the Usp46 complex restricts ISC proliferation but is dispensable for asymmetric stem cell division, properties shared with known positive regulators in the Wingless signaling pathway.

In the adult posterior midgut, the ability of enterocytes to non-autonomously restrict the proliferation of neighboring ISCs requires Wingless signaling^42,45^. To determine whether the Usp46 complex has a similar role, we generated control wild-type clones, *Usp46* null mutant clones, *Wdr20* null mutant clones, and *Uaf1* null mutant clones in the posterior midgut and analyzed the effects on neighboring stem and progenitor cells, which were marked with *esg-lacZ*, or ISCs only, which were marked with *Delta-lacZ*. We observed that the number of stem and progenitor cells surrounding *Usp46* mutant clones was increased by comparison to wild-type clones (Fig. S17A–D, G). Similarly, a non-autonomous increase in ISC/progenitor cell number was observed upon loss of either *Wdr20* or *Uaf1* (Fig. S17E–G and Fig. S18G–M). To determine whether the aberrantly increased ISCs resulted from overproliferation, we compared the mitotic index in posterior midguts harboring either wild-type control clones or *Usp46* mutant clones. We found a significant increase in phospho-histone H3, a marker for mitosis, near *Usp46* mutant clones (Fig. S19B, F), *Wdr20* mutant clones (Fig. S19C, F), and *Uaf1* mutant clones (Fig. S19D, F), as also occurred near *arrow* mutant clones (Fig. S19E, F), and following inactivation of other Wingless pathway components^42,45^. These findings reveal that in a manner similar to known Wingless pathway components, the Usp46 complex non-autonomously restricts proliferation of neighboring ISCs, providing additional evidence that Usp46 promotes Wingless signaling during intestinal homeostasis.

### Genetic interaction between Arrow/LRP6 and the Usp46 complex

To hierarchically order Usp46 complex activity in the Wingless pathway, we used the β-catenin destruction complex scaffold Axin as a reference point in genetic epistasis analysis. Axin inactivation results in aberrantly increased levels of cytoplasmic/nuclear β-catenin and the constitutive activation of Wingless signaling^50^. In the wild-type third instar larval wing imaginal disc, the Wingless target gene reporter *nkd-lacZ* is expressed in a broad region that surrounds the D-V boundary (Fig. S20A–C). In contrast, *nkd-lacZ* is expressed ectopically in *Axin* null mutant clones, regardless of their location in the wing disc (Fig. S20D–F). Ectopic *nkd-lacZ* expression was comparable in *Axin* single mutant clones and *Usp46 Axin* double null mutant clones, suggesting that Usp46 acts upstream of the destruction complex (Fig. S20G–I). Similarly, ectopic *nkd-lacZ* expression was comparable in *Axin* single mutant clones and *Wdr20 Axin* double null mutant clones (Fig. S20J–L). These findings suggest that the Usp46 complex acts upstream of the β-catenin destruction complex.

**Fig. 5:**
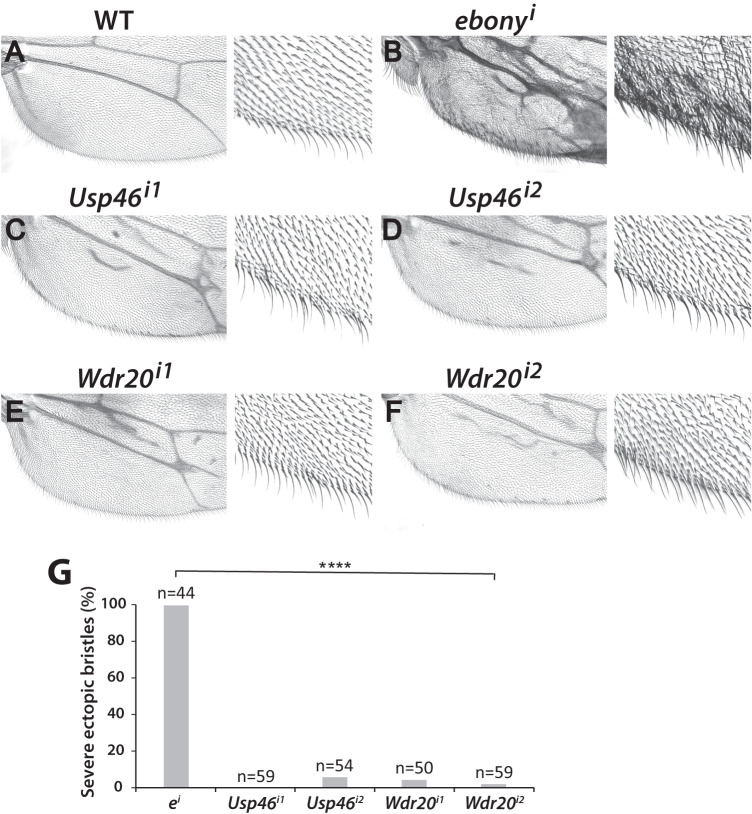
RNAi-mediated depletion of Usp46 complex components reduces Arrow-induced ectopic Wingless signaling. **A** Wild-type wing. Higher magnification of the posterior wing margin is shown on the right. **B** Overexpression of *arrow* with the *hh-Gal4* driver activates Wingless signaling, resulting in the formation of ectopic sensory bristles within the adult wing blade. Formation of ectopic bristles is not rescued by RNAi-mediated depletion of the *ebony* control. **C**–**F** Expression of two different *Usp46* RNAi (**C**, **D**) or *Wdr20* RNAi (**E**, **F**) constructs with *hh-Gal4* reduced the number of ectopic bristles induced by Arrow overexpression. **G** Quantification of percentage of flies with severe ectopic wing bristles resulting from Arrow overexpression coupled with RNAi-mediated knockdown of *Usp46*, *Wdr20*, or *ebony*. N is the number of flies analyzed. ****p < 0.0001 (0 for all genotypes, one-tailed t-test). Source data are provided in the Source Data file.

**Fig. 6:**
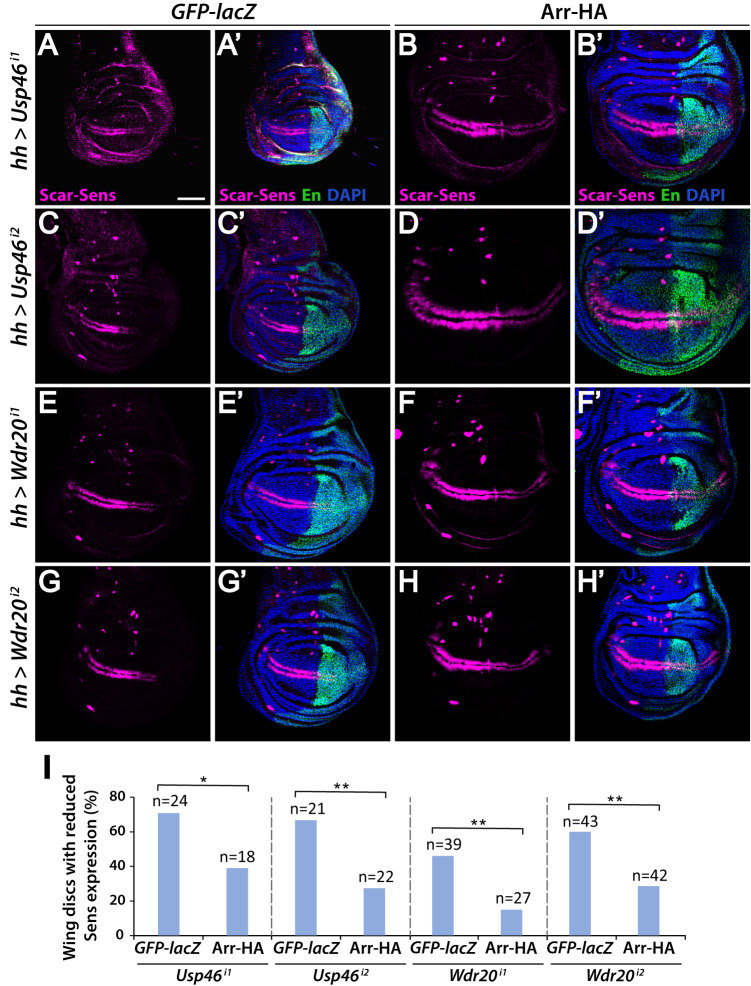
Arrow expression rescues loss of Sens resulting from RNAi-mediated depletion of Usp46 complex components. **A**–**H** *hh-Gal4-*driven expression in third instar larval wing discs of RNAi constructs targeting *Usp46* (**A**, **C**) and *Wdr20* (**E**, **G**) results in decreased Sens (magenta) in the posterior compartment (marked by Engrailed (En, green)). Sens expression is rescued by co-expression of Arrow (**B**, **D**, **F**, **H**). *GFP-lacZ* is expressed in control discs (**A**, **C**, **E** and **G**). DAPI (blue) marks nuclei. Scale bar: 50 µM. Dorsal, top and posterior, right. **I** Quantification is shown as percentage of discs of each genotype with decreased Sens. N is the number of discs analyzed. *p < 0.05 (0.022), **p < 0.01 (p values in order: 0.006, 0.0041, and 0.002, one-tailed *t*-test). Source data are provided in the Source Data file.

We therefore tested the hypothesis that the Usp46 complex acts at the level of the Wingless receptor complex using genetic interaction experiments. In the developing wing, the ectopic activation of Wingless signaling induces formation of ectopic sensory bristles in the wing blade^35,50,51^. This phenotype is recapitulated by overexpression of Arrow (the *Drosophila* ortholog of the Wnt receptor LRP6)^52^ using the *hh-Gal4* wing driver (Fig. 5A, B, G). RNAi-mediated knock down of *Usp46* in the wing rescued this Arrow overexpression phenotype (Fig. 5C, G). An independent *Usp46* RNAi construct yielded the same results (Fig. 5D, G), as did depletion of *Wdr20* using two independent RNAi constructs (Fig. 5E–G), in contrast with the negative control RNAi construct targeting *ebony* (Fig. 5B, G). Similarly, when driven by the *C96-Gal4* driver, the Arrow overexpression phenotype was rescued by depletion of either Usp46 or Wdr20 (Fig. S21). These findings reveal a strong genetic interaction between Arrow and the Usp46 complex, supporting the hypothesis that Arrow is a Usp46 substrate. Further supporting this hypothesis, the decreased expression of the Wingless target gene *senseless* resulting from knock down of either Usp46 (Fig. 6A, C, I) or Wdr20 (Fig. 6E, G, I) was rescued by Arrow overexpression (Fig. 6B, D, F, H, I).

### The Usp46 complex increases cell surface Arrow/LRP6 by decreasing its ubiquitylation

**Fig. 7:**
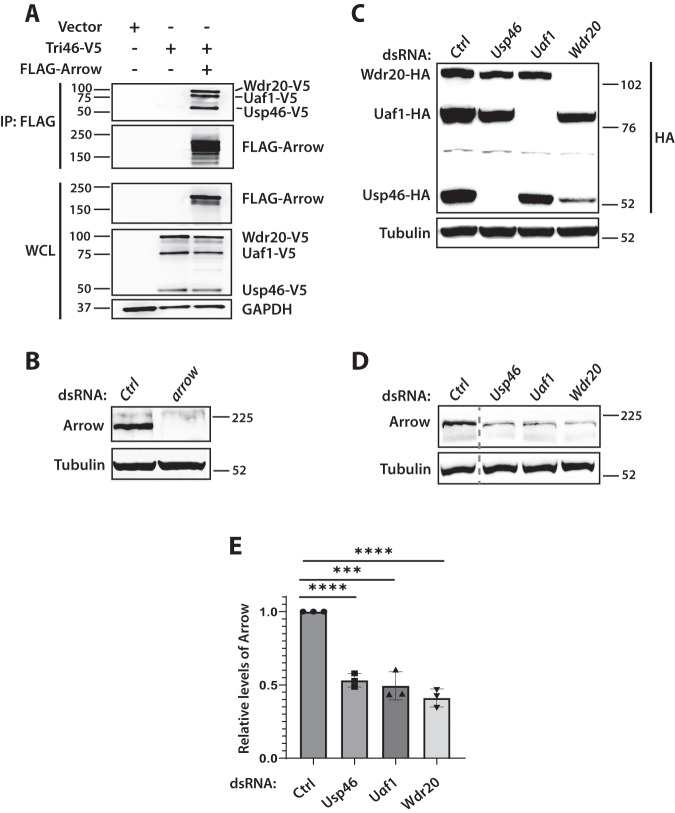
The Usp46 complex interacts with Arrow and regulates steady-state Arrow levels. **A** V5-tagged Usp46 complex components co-immunoprecipitate with FLAG-tagged Arrow. HEK293 cells were transfected with V5-tagged Usp46 complex components and FLAG-tagged Arrow as indicated; FLAG-Arrow was immunoprecipitated (IP) with anti-FLAG conjugated beads. Co-immunoprecipitated V5-tagged Usp46 complex components were detected by immunoblotting. WCL = whole cell lysates. A representative immunoblot (*n* = 3 independent experiments) is shown. **B** Arrow antibody specificity and knockdown efficiency. RNAi-mediated knockdown with dsRNAs targeting Ctrl (*white* negative control) or *arrow* demonstrate efficient Arrow knockdown and the specificity of the Arrow antibody. A representative immunoblot (*n* = 3 independent experiments) is shown. **C** Efficient RNAi-mediated knockdown of the Usp46 complex. Drosophila S2R+ cells were transfected with HA-tagged Usp46 complex components, followed by knockdown with indicated dsRNAs. A representative immunoblot (*n* = 3 independent experiments) is shown. **D** RNAi-mediated knockdown of the Usp46 complex decreases steady-state levels of Arrow. *Drosophila* S2R+ cells were treated with Ctrl or Usp46 complex dsRNAs, followed by immunoblotting with Arrow antibody. RNAi-mediated knockdown of the Usp46 complex resulted in decreased Arrow levels. Tubulin was used as a loading control. Dashed line: lanes between Ctrl and Usp46 were removed. A representative immunoblot (*n* = 3 independent experiments) is shown. **E** Quantification of Arrow levels normalized to tubulin, mean ± SD, *n* = 3. ****p < 0.0001 (6E-5 for *Usp46* and 7.8E-5 *for Wdr20*), ***p < 0.001 (8E-4 for *Uaf1*) (two tailed t-test). Source data are provided in the Source Data file.

Based on these genetic interactions, we tested whether the Usp46 complex interacts with and regulates Arrow in cultured cells. We found that V5-tagged Usp46 complex components co-immunoprecipitated with Flag-tagged Arrow when expressed in HEK293 cells (Fig. 7A). Furthermore, to determine whether the endogenous Usp46 complex regulates endogenous Arrow, we performed RNAi-mediated knock down of the Usp46 complex in *Drosophila* embryonic S2R+ cells and evaluated Arrow levels. We first verified the specificity of an Arrow polyclonal antibody (Fig. 7B) and confirmed the efficiency of Usp46 complex knockdown (Fig. 7C). Of note, this analysis also revealed that Wdr20, and to a lesser extent Uaf1, stabilized Usp46, as knockdown of either Uaf1 or Wdr20 reduced Usp46 levels (Fig. 7C). Conversely, overexpression of either Wdr20 or Uaf1 increased the levels of Usp46 (Fig. S22). The stabilization of endogenous Usp46 by Uaf1 and Wdr20 was confirmed in vivo by knockdown of endogenous Uaf1 or Wdr20 in larval wing discs (Fig. S23). Supporting these results, a similar stabilizing effect of UAF1 on human USP12 was found previously^17^.

**Fig. 8:**
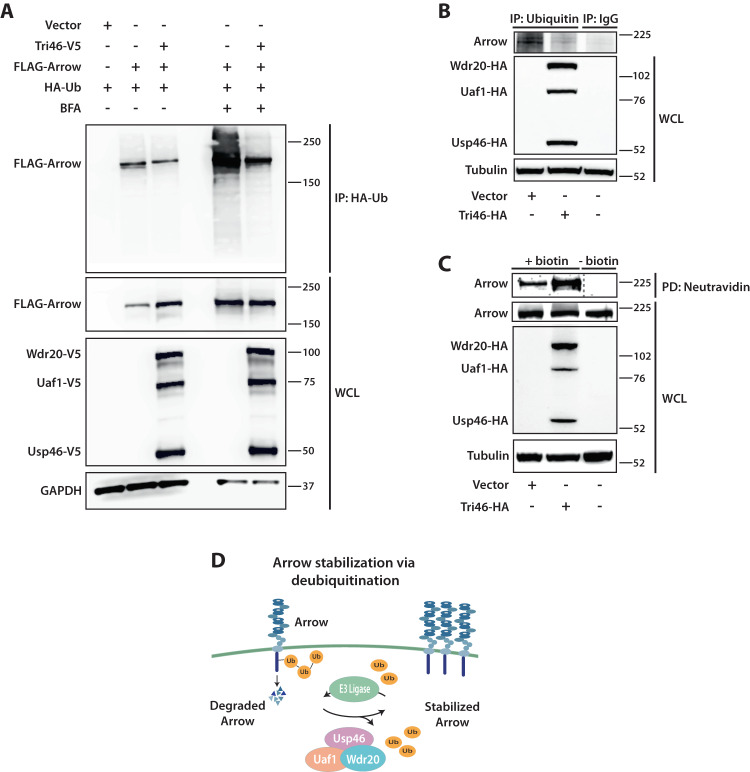
The Usp46 complex deubiquitylates and stabilizes Arrow at the cell surface. **A** The Usp46 complex deubiquitylates Arrow. HEK293 cells were transfected with FLAG-tagged Arrow, V5-tagged Usp46 complex, and HA-tagged Ubiquitin. Lysates were immunoprecipitated with HA antibody and blotted with FLAG antibody. Co-transfection of V5-Usp46 complex components with FLAG-Arrow decreased the levels of ubiquitylated Arrow. Treatment with bafilomycin A (BFA) increased ubiquitylated Arrow, which decreased upon co-transfection of V5-Usp46 complex components (Tri46-V5). A representative immunoblot (*n* = 3 independent experiments) is shown. **B** The Usp46 complex (Tri46-HA) decreased ubiquitylation of endogenous Arrow. *Drosophila* S2R+ cells were transfected with the indicated plasmids. Lysates were immunoprecipitated with Ubiquitin antibody or control IgG and analyzed by immunoblot with Arrow antibody. A representative immunoblot (*n* = 3 independent experiments) is shown. **C** The Usp46 complex (Tri46-HA) increases cell surface levels of Arrow. S2R+ cells were transfected with Usp46 complex components as indicated. Following cell surface biotinylation, lysates were subjected to neutravidin pull down and immunoblotted for endogenous Arrow. Tubulin was used as a loading control. WCL whole cell lysates. Dashed line: A lane between plus biotin and minus biotin that contained the protein ladder was removed. A representative immunoblot (*n* = 3 independent experiments) is shown. For (**A**–**C**) source data are provided in the Source Data file. **D** Model for the regulation of Arrow/LRP6 by the Usp46 complex in Wingless/Wnt signaling. Regulation of cell surface Arrow abundance is mediated by a tightly controlled balance in the opposing processes of ubiquitylation and deubiquitylation that is essential for achieving concentration-dependent signaling responses in the morphogen gradient.

RNAi-mediated reduction of any one of the three Usp46 complex components decreased Arrow levels, in contrast with RNAi knockdown of the negative control gene *white* (Fig. 7D, E). To rule out off-target effects, we tested a second set of dsRNAs targeting components of the Usp46 complex, which also resulted in decreased Arrow levels (Fig. S22B, C). Conversely, expression of the *Drosophila* Usp46 complex stabilized Arrow in HEK293 cells (Fig. 8A). These findings support the conclusion that the Usp46 complex interacts with and increases Arrow stability.

To determine whether the Usp46 complex stabilizes Arrow by reducing its ubiquitylation, we expressed HA-tagged ubiquitin in HEK293 cells followed by co-immunoprecipitation assays. To facilitate detection of ubiquitylated Arrow, cells were treated with the lysosome inhibitor bafilomycin A to block Arrow degradation. We found that the Usp46 complex markedly decreased levels of ubiquitylated Arrow (Fig. 8A). Similarly, the Usp46 complex decreased endogenous levels of ubiquitylated Arrow in S2R+ cells (Fig. 8B). Finally, to test if the Usp46 complex promotes the deubiquitylation and resultant stabilization of the plasma membrane pool of endogenous Arrow, we performed cell surface biotinylation of S2R+ cells. These findings revealed that the Usp46 complex increased Arrow levels at the cell surface (Fig. 8C). These results provide evidence that Arrow deubiquitylation by the Usp46 complex increases Arrow levels at the cell surface and thereby promotes Wingless signaling (Fig. 8D**)**.

**Fig. 9:**
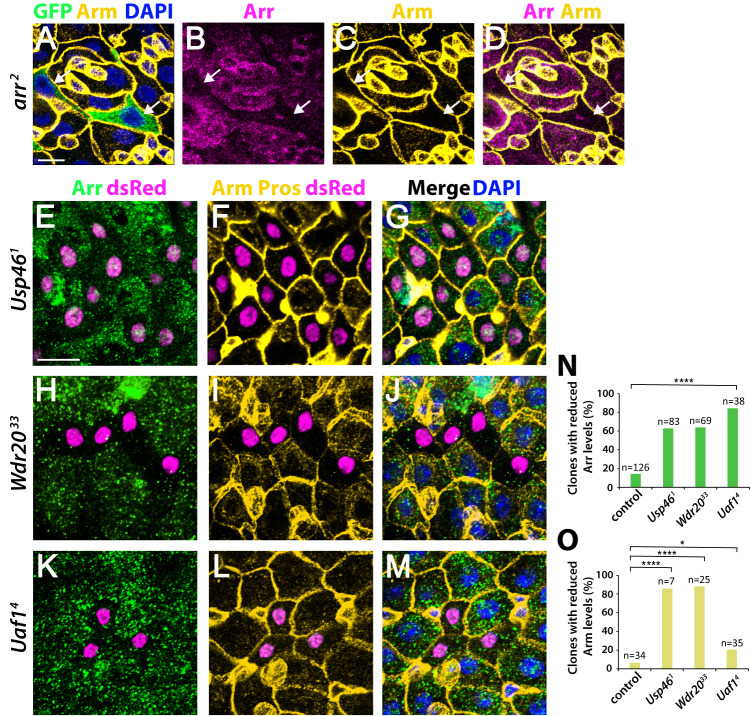
Usp46 complex inactivation reduces Arrow levels in a cell autonomous manner. **A**–**D** *arr*^*2*^ null mutant clones (green) in the adult posterior midgut. Upon Arrow loss, the Arrow signal intensity is decreased at the cell membrane and in the cytoplasm, confirming the specificity of the Arrow antisera. Additionally, cytoplasmic Arm (yellow) is reduced in Wingless-responding cells upon Arrow loss. **E**–**G** *Usp46*^*1*^ null mutant clones (magenta) in the adult posterior midgut. When Usp46 is inactivated, the levels of cell membrane-associated and cytoplasmic Arrow (green) are decreased cell-autonomously, as is cytoplasmic Arm (yellow). **H**–**J** *Wdr20*^*33*^ null mutant clones (magenta) in the adult posterior midgut. Both Arrow (green) and cytoplasmic Arm (yellow) are decreased cell autonomously when Wdr20 is inactivated. Some cells also display a non-autonomous decrease in cell membrane-associated Arm. **K**–**M** *Uaf1*^*4*^ null mutant clones (magenta) in the adult posterior midgut. When Uaf1 is inactivated, the levels of cell membrane-associated and cytoplasmic Arrow (green) are decreased cell-autonomously. Some cells also display a reduction in membrane-associated Arm (yellow). DAPI (blue) marks nuclei. Scale bars (A-D) and (E-M): 20 µM (**N**, **O**) Quantification is shown as percentage of clones of each genotype with decreased Arrow (**N**) or Arm (**O**). N is the number of clones analyzed, *p < 0.05 (0.043 for *Uaf1*^*4*^ in **O**), ****p < 0.0001 (0 for all genotypes in **N**, 0 for *Wdr20*^*33*^ and 1E-5 for *Usp46*^*1*^ in **O**, one-tailed *t*-test). Source data are provided in the Source Data file.

Building on these findings, we tested the Usp46-dependent regulation of Arrow/LRP6 abundance in vivo. In the wild-type adult intestine, endogenous Arrow was observed at the plasma membrane and in the cytoplasm, representing both fully processed and newly synthesized Arrow during its biogenesis (Fig. 9A–D). Confirming the specificity of the Arrow antibody, Arrow signal decreased in a cell-autonomous manner in clones of *Arrow* null mutant cells (Fig. 9A, B, D). The cytoplasmic accumulation of Armadillo (Arm)/β-catenin also decreased upon Arrow loss (Fig. 9A, C, D), indicating an expected block in transduction of Wingless signaling. Similarly, in clones of *Usp46* null mutant cells, Arrow signal decreased in a cell-autonomous manner by comparison with the adjacent wild-type cells (Fig. 9E, G, N). Cytoplasmic Arm/β-catenin levels also decreased in *Usp46* null mutant clones, whereas the levels of membrane-associated Arm did not (Fig. 9F, G, O). The subcellular distribution and levels of the basolateral cell membrane marker Discs Large (Dlg) and the DNA marker DAPI were normal in *Usp46* mutant cells, indicating retention of normal cell polarity and the absence of aberrant apoptosis (Fig. 9G and Fig. S24A, C). An independently derived *Usp46* null allele displayed the same results (Fig. S25A–C, N, O). This reduction in the cytoplasmic/signaling pool of Arm/β-catenin provides an explanation for the decreased Wingless target gene activation observed in clones of *Usp46* null mutant intestinal cells (Fig. 2E, F, H, I and Fig. 3A–C).

Inactivation of Wdr20 or Uaf1 in vivo also reduced Arrow abundance. Arrow levels decreased cell-autonomously in *Wdr20* (Fig. 9H, J, N) and *Uaf1* (Fig. 9K, M, N) null mutant clones by comparison with their wild-type neighbors. Additional *Wdr20* and *Uaf1* null alleles similarly resulted in a cell-autonomous decrease in Arrow levels (Fig. S25D, F, G, I, N). Moreover, as observed in *Usp46* mutant cells, the cytoplasmic accumulation of Arm/β-catenin was reduced in *Wdr20* mutant cells (Fig. 9I, J, O and Fig. S25E, F, O). These findings provide in vivo evidence that the Usp46 complex increases signaling strength by stabilizing Arrow/LRP6 and consequently promoting the cytoplasmic accumulation of Arm/β-catenin in Wingless-responding cells.

## Discussion

We have identified an evolutionarily conserved tripartite deubiquitylase complex, composed of the catalytic subunit Usp46 and its allosteric regulators Wdr20 and Uaf1, that increases the abundance of the Wingless/Wnt receptor Arrow/LRP6 in *Drosophila* (Fig. 8D). By reducing Arrow/LRP6 ubiquitylation and turnover and thus increasing cell surface Arrow levels, Usp46 enhances the sensitivity of target cells to Wingless stimulation. The Usp46 complex thereby increases the amplitude and spatial range of signaling responses that mediate the concentration-dependent transcriptional regulation of Wingless target genes. Consequently, depletion of the Usp46 complex disrupts Wingless-directed development and tissue homeostasis. Supporting these findings, a genome-wide screen identified Usp46 and Uaf1 among many potential co-regulators that act at or above the level of Wnt receptors^53^. Our results provide evidence that the function of Usp46 in Arrow/LRP6 deubiquitylation is essential for the precise activation of signaling throughout the Wingless/Wnt morphogen gradient.

Whereas the mechanisms by which Wnt ligands spread from their source of synthesis to form morphogen gradients have been well-investigated^8,12–15^, how target cells fine-tune signaling to achieve precision in the gradient has remained unclear. Uncovering these mechanisms will inform our understanding of Wnt signaling in physiological contexts and our ability to intervene in pathological contexts arising from Wnt pathway deregulation. The findings herein, coupled with those from vertebrate models^54–56^, reveal that regulation of Wnt receptor abundance, mediated by a tightly controlled balance in the opposing processes of ubiquitylation and deubiquitylation, is essential for concentration-dependent signaling responses following Wnt stimulation. Loss of Wnt receptor ubiquitylases and deubiquitylases in vivo shifts the signaling strength within the morphogen gradient, rather than constitutively activating or abrogating signaling. For example, inactivation of Zinc and Ring Finger Protein 3 (ZNRF3), a membrane-bound ubiquitin ligase that targets Wnt receptors for clearance from the plasma membrane and subsequent degradation^57,58^, disrupts a Wnt signaling gradient in the developing adrenal gland by increasing signaling strength to a moderate level in a region of the cortex where signaling is normally low^54–56^. As a consequence, ZNRF3 loss results in adrenal hyperplasia and aberrant cell fate specification^54^. Conversely, loss of the Usp46 deubiquitylase attenuates, but does not abolish signaling in much of the Wingless gradient in the *Drosophila* posterior midgut, and thereby disrupts Wingless-regulated tissue homeostasis. Whereas ZNRF3 and its paralog Ring Finger Protein 43 (RNF43) are known to promote Wnt receptor proteolysis in vertebrates, neither of these ubiquitylases is conserved in *Drosophila*. As such, the E3 ubiquityase that destabilizes Arrow by counteracting Usp46 awaits discovery and is predicted to have a key function in the negative regulation of Wingless signaling. Vertebrate homolog(s) of this E3 ubiquitylase may similarly oppose vertebrate USP46, since the role of the USP46 complex in Arrow/LRP6 stabilization is evolutionarily conserved, as we report in an accompanying paper^59^.

Our findings identify a component required for Wnt receptor regulation and build on other mechanisms that control Arrow/LRP6 abundance. First, in the wing disc, the genes encoding the Wingless receptors Arrow and Frizzled 2 are transcriptionally repressed in cells responding to Wingless stimulation^52,60,61^. The degree of transcriptional repression correlates directly with the Wingless concentration, such that receptor levels are lowest in cells near the Wingless source and increase as a function of distance from this source. As high receptor levels stabilize Wingless, this setup is thought to enhance the spread of Wingless from its source (where receptor levels are low) to distant cells (where receptor levels are high). Second, following proper post-translational folding of LRP6, the endoplasmic reticulum (ER)-specific Ubiquitin-specific protease 19 (USP19) promotes ER exit of LRP6 via deubiquitylation of a single lysine residue^62^. Third, an ER chaperone protein dedicated to the low-density lipoprotein family of receptors, Mesoderm development (Mesd or Boca in *Drosophila*), is essential for trafficking LRP6/Arrow through the secretory pathway and thereby facilitates Wnt-dependent patterning during development^63,64^. The Usp46 complex adds an essential layer of control — one that not only increases Arrow/LRP6 abundance at the cell surface in Wingless target cells but also is critical for precise, concentration-dependent signaling responses throughout the Wingless morphogen gradient.

Inactivating *RNF43* or *ZNRF3* mutations result in increased Wnt receptor levels in mammalian cells and promote the growth of numerous human cancers, including colorectal, endometrial, ovarian, pancreatic, gastric, and adrenocortical carcinomas^1,65^. An oncogenic role therefore exists for aberrantly elevated Wnt receptor levels and the resultant hypersensitivity they confer to Wnt stimulation. These clinical observations suggest that reduction in receptor abundance may provide a therapeutic strategy for a subset of Wnt-driven cancers^58,66^. Whether a Wnt receptor deubiquitylase of the USP class would be amenable to specific targeting with selective inhibitors had been uncertain due to structural similarity among members of this enzyme family. However, more recent studies have documented the vulnerability of USP family deubiquitylases to specific inhibition by small molecules, raising their promise as therapeutic targets and the feasibility of this approach^67,68^. Accordingly, the identification of USP46 small molecule inhibitors may provide a novel therapeutic option to combat cancers dependent on Wnt ligand stimulation. Our identification of a conserved Wnt receptor stabilization mechanism may therefore have relevance not only for animal development and tissue homeostasis, but also Wnt-driven cancers.

## Methods

### Fly stocks and genetics

Fly crosses were performed at 25 °C unless otherwise indicated.

Reporters: *nkd-lacZ* (nls)^44^, *esg-lacZ*^69^, *esg-Gal4, UAS-GFP (esg* > *GFP)*^69^, *GBE-Su(H)-lacZ*^69^, *Delta-lacZ* (Bloomington Drosophila Stock Center (BDSC #11651)), *mScar:T2A:sens*^70^, and *fz3-GFP*^71^.

MARCM and hs-flp lines: MARCM 82B: *yw hs-flp UAS-CD8*::*GFP; tub-Gal4 FRT82B tub-Gal80/TM6B*^72^ and *yw hs-flp tub-Gal4 UAS-dsRed; FRT82B tub-Gal80/TM3, Ser*^73^.

MARCM 42D: *yw hs-flp UAS-(nls)GFP tub-Gal4; FRT42D tub-Gal80*^74^ and *yw hs-flp tub-Gal4 UAS-dsRed; FRT42D tub-Gal80/CyO*; *tub-Gal4/TM3 Sb*.

*hs-flp; 82B ubi-GFP* (BDSC#52012).

RNAi and Gal4 driver lines: The RNAi lines *Usp46*^*i1*^ (Vienna Drosophila Resource Center (VDRC) #27799), *Usp46*^*i2*^ (VDRC #100586), *Wdr20*^*i1*^ (VDRC #110609), *Wdr20*^*i2*^ (VDRC #42060), *Uaf1*^*i1*^ (VDRC #3810) *and y*^*i*^ (VDRC #106068) were expressed in third instar larval wing discs using *hh-Gal4*^75^ with *UAS-dcr2*^76^, or *C96-Gal4*^77,78^ with *UAS-dcr2* (BDSC #25757), or *ap-Gal4*^79^.

Other stocks: *arr*^*2 *^^52^, *Axin*^*s044230 *^^50^, *Usp46*^*MiMIC*^ (*yw*; *MiMIC* *Usp12-46*^*MI02353*^ *CG7029*^*MI02353*^,BDSC #35110)^80^, *Wdr20*^*Df*^ (*Df*(*3R*) *BSC524/TM6C*, BDSC #25025), *Usp46*^*Df*^ (*Df*(*3R*) *BSC618/TM6C*, BDSC #25693), *UAS-GFP::lacZ.nls* (BDSC #6452) and *UAS-Arrow-HA*^81^. Wild-type controls were *FRT42D*, *FRT82B*, and Canton S.

*Usp46*, *Wdr20* and *Uaf1* mutants: *Usp46*, *Wdr20* and *Uaf1* mutants (Supplementary Table 1) were generated using CRISPR/Cas9. CRISPR target sites were identified using https://flycrispr.org/target-finder/^82^. Four guide RNAs for each gene (Supplementary Table 2) were cloned into the *pCFD6: UAST::gRNA* plasmid^38^, following the protocol described at http://www.crisprflydesign.org/. Plasmids were injected by BestGene and integrated at the *attP40* site. gRNA-containing lines were crossed to *yw; UAS-Cas9 nos-Gal4::VP16* (BDSC #54593).

Knock in of V5 epitope tags into the endogenous *Usp46*, *Wdr20* and *Uaf1* genes: Epitope tagging of *Usp46*, *Wdr20* and *Uaf1* was performed using a co-CRISPR method^36^. Two guide RNAs (Supplementary Table 3) close to the stop codon were identified using https://flycrispr.org/target-finder/ and were cloned into *pCFD3: U6:3-gRNA*^83^, following the protocol described at http://www.crisprflydesign.org/. A template for homology-directed repair was generated by synthesizing a gene block (Integrated DNA Technologies, Inc.) that was cloned into the *pMiniT* *2.0* plasmid using the New England Biolabs PCR cloning kit. The gene blocks were 2 kb long, contained a linker sequence (AAGGGCCGAGCCGATCCCGCCTTCCTGTACAAGGTGGTCAGCTCCGCCACC) and a 3X V5 tag (GGTAAACCTATTCCTAATCCTCTCCTAGGTTTAGATTCTACTGCTGCCGGCAAGCCCATCCCCAACCCCTTGCTTGGCTTGGACTCCACCGCCGCAGGAAAACCAATACCAAATCCACTTCTCGGACTTGATTCAACA) upstream of the stop codon, and ~900 bp homology arms on either side of the linker and tag. DNA mixes containing 100 ng/ml of each pCFD3-gRNA plasmid, including a gRNA for the *ebony* gene, and 500 ng/ml of the repair template were injected into *nos-Cas9* embryos by BestGene.

### Tissue-specific CRISPR-generated *Usp46*, *Uaf1*, and *Wdr20* mutations

CRISPR target sites were identified using https://flycrispr.org/target-finder/^82^. gRNA sequences are listed in Supplementary Table 4. Two guide RNAs for each gene were cloned into the *pCFD6: UAST::gRNA* plasmid. Plasmids were injected by BestGene and integrated at the *attP40* site or the *attP2* site for the *Uaf1* gRNA^1^. Wing-specific mutations were generated by crossing the gRNA lines to *hs-flp; UAS-uMCas9; hh-Gal4/TM6B*^39^ (VDRC #340019).

### Clonal analysis

Mitotic clones in the gut were generated using the MARCM system^84^. Clones were induced by one or two 2-hour heat shocks of 1st and 2nd instar larvae at 37 °C for the *nkd-lacZ* staining or a 2-hour heat shock of early 3rd instar larvae at 37 °C for *fz3-GFP* and Arrow immunostaining. Clones were examined 1–2 days after eclosion for *nkd-lacZ* staining, 5–10 days after eclosion *for fz3-GFP* staining, and 9–11 days after eclosion for Arrow staining.

To generate clones in the adult gut, flies were heat shocked for 30 min in a 37 °C water bath 4 days after eclosion. After heat shock, the flies were maintained at 25 °C for 5 days before analysis.

Clones in wing discs were generated by one or two 2-h heat shocks of 1st and 2nd instar larvae at 37 °C.

### Immunohistochemistry

Adult guts were dissected in PBS and fixed in 4% paraformaldehyde for 45 mins at room temperature; wing discs from 3^rd^ instar larvae were fixed in 4% paraformaldehyde for 20 mins at room temperature. Tissues were washed with PBS + 0.1% Triton X-100 and blocked with PBS + 0.1% Tween-20 + 10% BSA for 1 h at room temperature. The samples were incubated with primary antibody (diluted in PBS + 0.5% Triton X-100) at 4 °C for 1–3 days. Secondary antibody incubation was carried out at room temperature for 2 h. The samples were subsequently stained with DAPI (2 μg/ml) and mounted in Prolong Gold Antifade Reagent (Invitrogen). Confocal images were captured on a Nikon A1RSi laser scanning confocal microscope, Nikon CSU-W1 spinning disk confocal microscope, or Nikon Yokogawa CSU-W1 SoRa spinning disk confocal microscope and processed with Adobe Photoshop / Illustrator software from Adobe Suite 2023. Adult wings were mounted in Mowiol and their images acquired using a Leica MZFLIII stereomicroscope with a Zeiss Axiocam 208 camera and Nikon Zen 3.0 software.

### Plasmids

pUAST-Usp46-Flag-HA (UFO05132, Stock 1643053), pUAST-Uaf1-Flag-HA (UFO07270, Stock 1642047), and pUAST-Wdr20-Flag-HA (UFO09009, Stock 1643053) were obtained from the Drosophila Genomics Resource Center (DGRC). pCS2-Usp46-V5, pCS2-Uaf1-V5, pCS2-Wdr20-V5, pCS2-3XFLAG-Arrow were synthesized by Gene Universal. The V5 tag GKPIPNPLLGLDST was inserted at the carboxy-terminus of the Usp46 complex components and preceded by the linker sequence GGGGS. A 3X FLAG tag in Arrow was inserted after the signal sequence at amino acid 67. The Arrow sequence surrounding the insertion site, with the 3XFLAG tag in brackets is: NVH[DYKDHDGDYKDHDIDYKDDDD]KGGS. HA-ubiquitin was cloned into pCS2^70^.

### Cell culture and DNA transfection

HEK293T cells were purchased from the American Type Culture Collection (ATCC) and maintained in DMEM supplemented with 8% fetal bovine serum. S2R+ cells were purchased from the Drosophila Genomics Research Center (DGRC) and maintained at 25˚C in Schneider’s complete medium: Schneider’s Drosophila medium with L-glutamine (Gibco) supplemented with 10% FBS (Gibco) and 0.1 mg/mL penicillin/streptomycin (Invitrogen). Cells were seeded in plates 24 h before transfection and 20ug of total plasmid was transiently transfected using calcium phosphate. 48 h post-transfection, cells were lysed using NP-40 lysis buffer (50 mM Tris-HCl pH 8.0, 100 mM NaCl, 1% NP-40, 10% glycerol, 1.5 mM EDTA pH 8.0, supplemented with 1X Roche protease inhibitor cocktail).

### dsRNA generation and RNAi-mediated knockdown

Double-stranded RNA (dsRNA) templates of 200-900 nucleotides targeting *Usp46*, *Uaf1*, *Wdr20*, *arrow*, or *white* (negative control) were synthesized by PCR from genomic DNA extracted from S2R+ cells. The PCR templates contained T7 promoter sequences on both ends and were amplified using primers pairs listed in Supplementary Table 5. dsRNAs were transcribed from PCR-generated templates using the T7 Megascript kit (Ambion) according to manufacturer’s instructions. For RNAi-mediated knockdown, S2R+ cells were plated in 6 well plates with 1 mL of serum-free, antibiotic-free Schneider’s medium + L-glutamine. 30 μg of each dsRNA were added to the medium and cells were incubated with gentle rotation at 25˚C for 1 h. Following incubation, 1 mL of complete medium was added and cells were incubated at 25˚C. After 24 h, the medium was removed. This procedure was repeated once every 24 h for a total of 96 h. For Arrow knockdown, an equivalent amount of *Arrow-4* and *Arrow-5* dsRNA (15 μg of each) were mixed and added to the medium; 30 μg of the *white* dsRNA control was used. On the 5th day of dsRNA treatment (at 96 h), cells were lysed via the RIPA lysis method described below.

### Immunoblotting and immunoprecipitation

#### Immunoblotting

At 48 h post-transfection, S2R+ cells were resuspended in media and spun at 400Xg for 2 mins. The media was aspirated, the cells were washed with 1 ml of cold 1X PBS and then spun at 400Xg for 2 mins. The PBS was then aspirated and the pellet was resuspended in 100 μl RIPA buffer (50 mM Tris pH 7.5, 500 mM NaCl, 0.1% Triton, 1% NP-40, 0.1% SDS, supplemented with 1X protease inhibitor cocktail) or NP-40 lysis buffer (50 mM Tris-HCl pH 8.0, 100 mM NaCl, 1% NP-40, 10% glycerol, 1.5 mM EDTA pH 8.0). Lysates were incubated on ice for 15 min and then spun at maximum speed at 4˚C for 15 min. Lysates were stored at −20˚C. Quantification of immunoblots was performed with ImageJ.

#### Immunoprecipitation

HEK293T cells were transfected with 3.5 μg each of V5-tagged Usp46, Uaf1 and Wdr20 plasmids, and 5 μg of either Arrow-FLAG or empty vector using calcium phosphate. After 48 h, cells were lysed using non-denaturing lysis buffer (NDLB; 50 mM Tris pH 7.5, 300 mM NaCl, 1% Triton X-100, 5 mM EDTA, 0.2 g/L sodium azide). Cell lysates were immunoprecipitated using FLAG M2 affinity agarose resin (Sigma), which were washed five times in NDLB, and bound protein eluted into sample buffer. Lysates and immunoprecipitated protein were probed with FLAG, V5, and GAPDH antibodies.

### Ubiquitylation assays

HEK293T cells were co-transfected with HA-ubiquitin and FLAG-Arrow plasmids, along with either empty vector or V5-tagged *Drosophila* Usp46, Uaf1 and Wdr20 plasmids. 2 μg of each plasmid, or the equivalent amount of empty vector were used for each transfection. Transfected cells were treated with 100 nM bafilomycin A for 16 h prior to lysis. Cell lysates were immunoprecipitated with HA antibody (12CA5) conjugated to Protein A/G agarose beads, and elutions were probed with FLAG antibody and V5 antibody. S2R+ cells were transfected with either empty vector or HA-tagged *Drosophila* Usp46, Uaf1 and Wdr20 plasmids. At 48 h post-transfection, cells were treated with MG132 (100um) and bafilomycin (100 nm) for 4 h prior to lysis. Cells were harvested and lysed in NP-40 lysis buffer (50 mM Tris-HCl pH 8.0, 100 mM NaCl, 1% NP-40, 10% glycerol, 1.5 mM EDTA pH 8.0 and supplemented with 1X Roche protease inhibitor cocktail). Lysates were incubated with Arrow antibody or IgG control overnight at 4˚C, followed by incubation with protein A/G-Sepharose beads (Santa Cruz) for 1 h at 4˚C. Beads were washed three times with wash buffer (50 mM Tris-HCl pH 8.0, 150 mM NaCl, 1% NP-40, 10% glycerol, 1.5 mM EDTA pH 8.0) supplemented with Roche protease inhibitor cocktail (1:100) and boiled in 4X sample buffer supplemented with 1 M DTT. Samples were resolved by SDS-PAGE and immunoblotted with the indicated antibodies.

### Antibodies for immunoblotting and immunoprecipitation

The primary antibodies used were mouse ubiquitin (1:500, P4D1, catalog #sc-8017) and mouse IgG (1 ug per ml, catalog #sc-2025) from Santa Cruz Biotechnology, rabbit FLAG (1:1000, catalog #20543-1-AP) from Proteintech, rabbit V5 (1:1000, D3H8Q, catalog # 13202) from Cell Signaling Technology, mouse GAPDH (1:500, 2G7) from the Developmental Studies Hybridoma Bank, rat HA (1:2000 for IB, 3F10, catalog #11867423001) from Roche, mouse HA (4ul per mg protein for IP, 12CA5, catalog #MA1-12429) from Thermo Fisher Scientific, mouse alpha-tubulin (1:10000, DM1A, catalog #T6199) from Sigma, and guinea pig Arrow (1:1000 for IB and 1:500 for IP)^85^. The secondary antibodies used were HRP-conjugated goat anti-mouse (1:10000, catalog #STAR207P) and goat anti-rat (1:10000, catalog #5204-2504) from BioRad, and goat anti-guinea pig (1:10000, catalog #6090-05) from SouthernBiotech.

### Antibodies for immunostaining

The primary antibodies used were mouse Discs Large (1:50, 4F3), mouse Armadillo (1:50, N27A1), mouse Prospero (1:100, MR1A), mouse Wingless (1:500, 4D4), and mouse Engrailed (1:50, 4D9) from the Developmental Studies Hybridoma Bank, chicken GFP (1:10000 catalog # ab13970) from Abcam, rabbit GFP (1:500, catalog # A-11122) and mouse V5 (1:500, SV5-Pk1, catalog # R960-25) from Thermo Fisher Scientific, rabbit dsRed (1:500 for Scar-Sens, catalog # 632496) from Clontech/TaKaRa, mouse beta-galactosidase (1:500, catalog # Z378B) from Promega, rabbit phospho-histone H3 (1:1000, Ser10, catalog # 06-570) from Millipore, rabbit Arrow (1:5000)^86^ and guinea pig Senseless (1:2000)^33^. The secondary antibodies used were goat or donkey Alexa Fluor 488, 555, or 647 conjugates (1:500) from Invitrogen, and goat or donkey Cy5 conjugates (1:500) from Life Technologies/Jackson Immunochemicals.

### Cell-surface biotinylation

S2R+ cells were prepared by resuspension in media and spun at 700 g for 5 min at 4˚C. Cells were then washed 3X with 5 ml pre-chilled modified PBS (1X PBS supplemented with 0.9 mM CaCl_2_ and 0.5 mM MgCl_2_) on ice. Cell surface proteins were biotinylated with 0.5 mg/ml EZ link Sulfo-NHS-SS-Biotin (Thermo Fisher Scientific) dissolved in modified PBS for 2 h with gentle rocking at 4˚C. The reaction was quenched by washing the cells 3X with 5 ml of ice-cold 50 mM Tris-HCl (pH 7.4) for 10 min with gentle rocking at 4˚C. Cells were then lysed in RIPA lysis buffer (50 mM Tris-HCl pH 7.4, 150 mM NaCl, 1% Triton X-100, 0.5% sodium deoxycholate, 0.1% SDS, 1 mM EDTA, supplemented with 1X Roche protease cocktail). Lysates were sonicated for 30 seconds and then centrifuged at 14,000 g for 10 min at 4˚C. After centrifugation, biotinylated proteins were pulled down with NeutrAvidin agarose beads (Pierce), and analyzed by SDS-PAGE, followed by immunoblotting.

### Lifespan assay

For the lifespan assay, wild-type or *Usp46*, *Wdr20*, or *Uaf1* mutant flies between 1 and 4 days after eclosion were placed in empty vials containing a 5 × 2.5 cm piece of filter paper soaked with 400 µl of a 5% sucrose solution. Up to fifteen flies were placed in each vial and reared at 29 °C. Flies were transferred to new vials with fresh sucrose solution each day and the number of survivors counted. Lifespan curves and statistical analysis were performed using OASIS 2 (Online Application for Survival Analysis 2)^87^.

### Quantification, Statistics and Reproducibility

For quantification of progenitor cells in the adult midgut, *esg* > *GFP* or *esg-lacZ* positive cells in a field of 0.051mm^2^ in the R5 region were counted. For quantification of enteroblasts, *GBE-Su(H)-lacZ*-positive cells in a field of 0.051 mm^2^ in the R5 region or R4/R5 boundary were counted.

A minimum of three independent replicates were performed for each experiment. Statistical tests were performed using Prism 9 (GraphPad Software, USA) or the GIGA P-value Calculator (https://www.gigacalculator.com/calculators/p-value-significance-calculator.php).

Quantification of *fz3-GFP* intensity was performed with image analysis tools in NIS-Elements software, AR5.30.01.

### Reporting summary

Further information on research design is available in the Nature Portfolio Reporting Summary linked to this article.

### Supplementary information

Supplementary Information

Reporting Summary

### Source data

Source Data

## Data Availability

All data supporting the findings of this study are available within the published article and the Supplementary Information files. Raw data and original gel images are included in the Source Data file, which is provided with this paper.
