# Supplementary material for: The USP46 deubiquitylase complex increases Wingless/Wnt signaling strength by stabilizing Arrow/LRP6
Source: Nat Commun. 2023 Oct 5;14:6174. doi: 10.1038/s41467-023-41843-0 (PMC10556106; doi:10.1038/s41467-023-41843-0)
Supplement: Supplementary file 1 — Supplementary Information [file 41467_2023_41843_MOESM1_ESM.pdf]

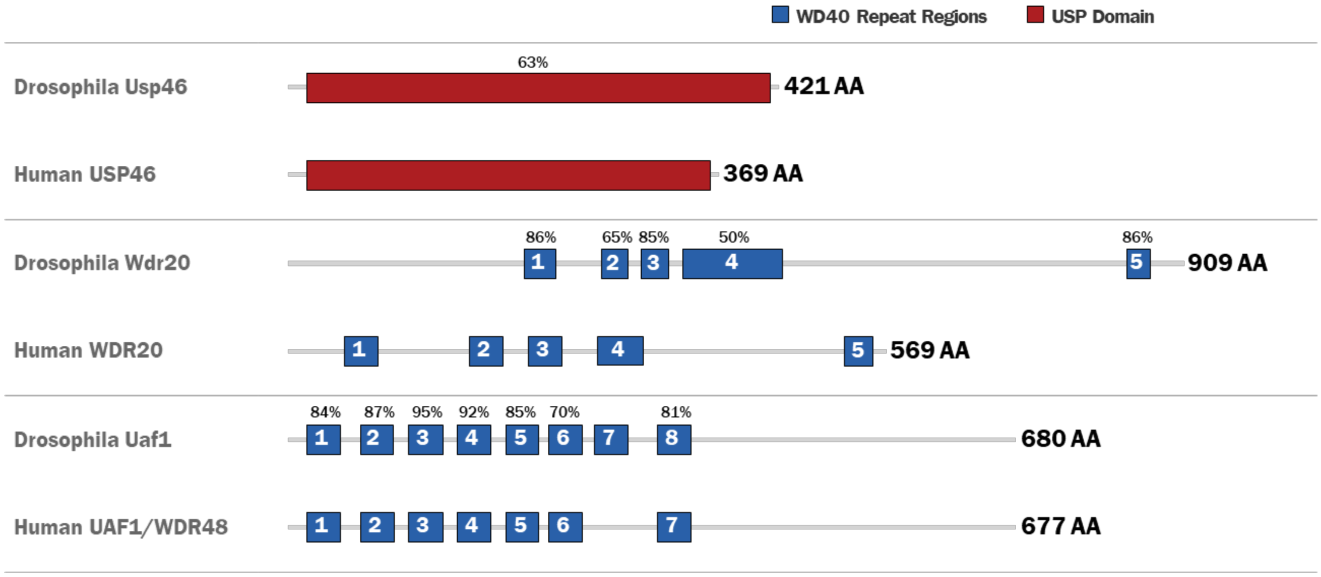

Figure S1. Evolutionary conservation between human and Drosophila Usp46 complex components.
Schematic representation of domains in the Drosophila and human USP46, WDR20, and UAF1 orthologs. The Ubiquitin specific protease (USP) domain (red) in USP46 and the WD40 repeat domains (blue) in WDR20 and UAF1 are indicated. Percent similarity in amino acid sequence within each domain between the Drosophila and human orthologs is indicated.

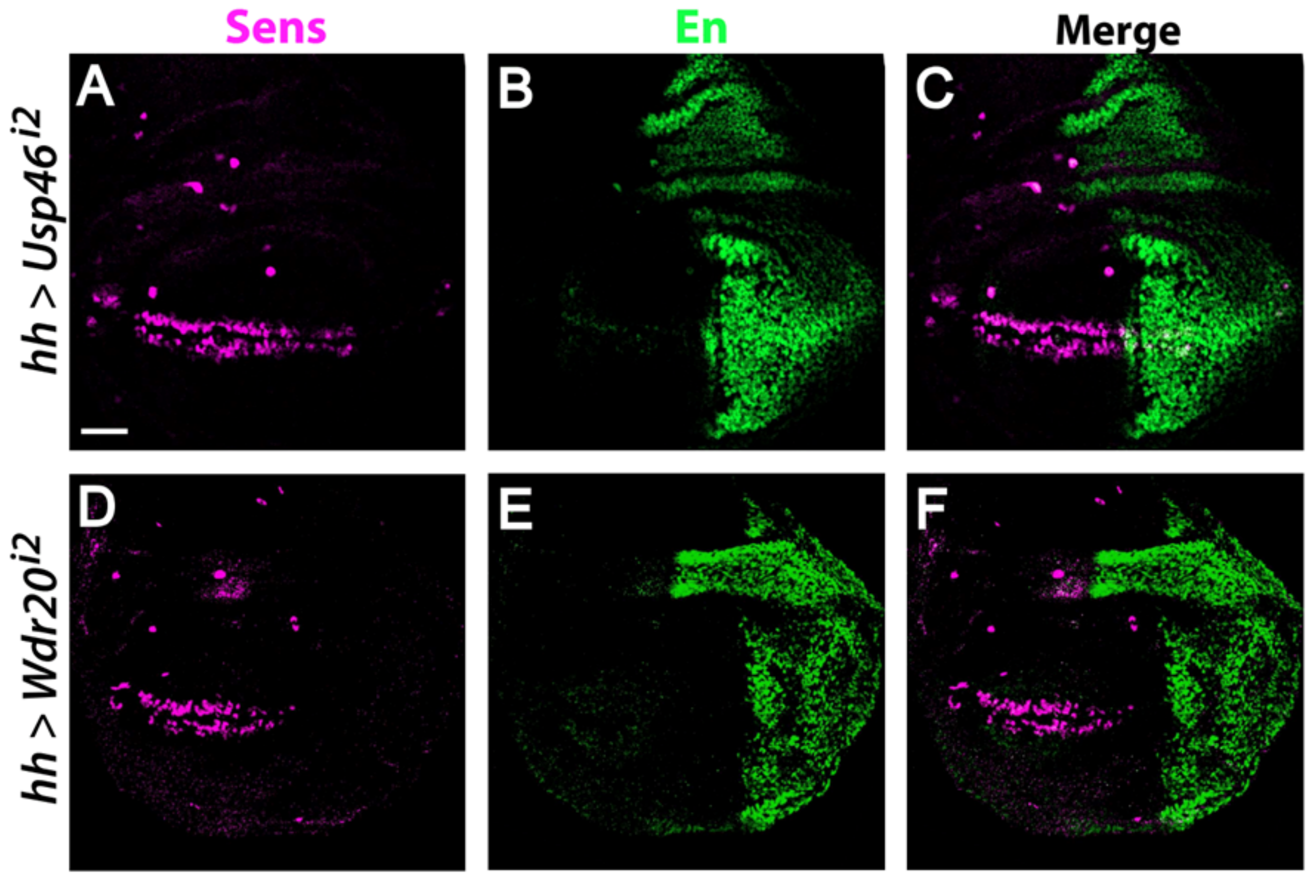

Figure S2. Depletion of Usp46 complex components in the wing disc with independent RNAi constructs.
(**A-F**) Independent RNAi constructs targeting *Usp46* (**A-C**) and *Wdr20* (**D-F**) driven by *hh-Gal4* result in decreased Sens (magenta) in the posterior wing disc, marked by En (green). Dorsal, top and posterior, right.

Scale bar (A-F): 20 μM

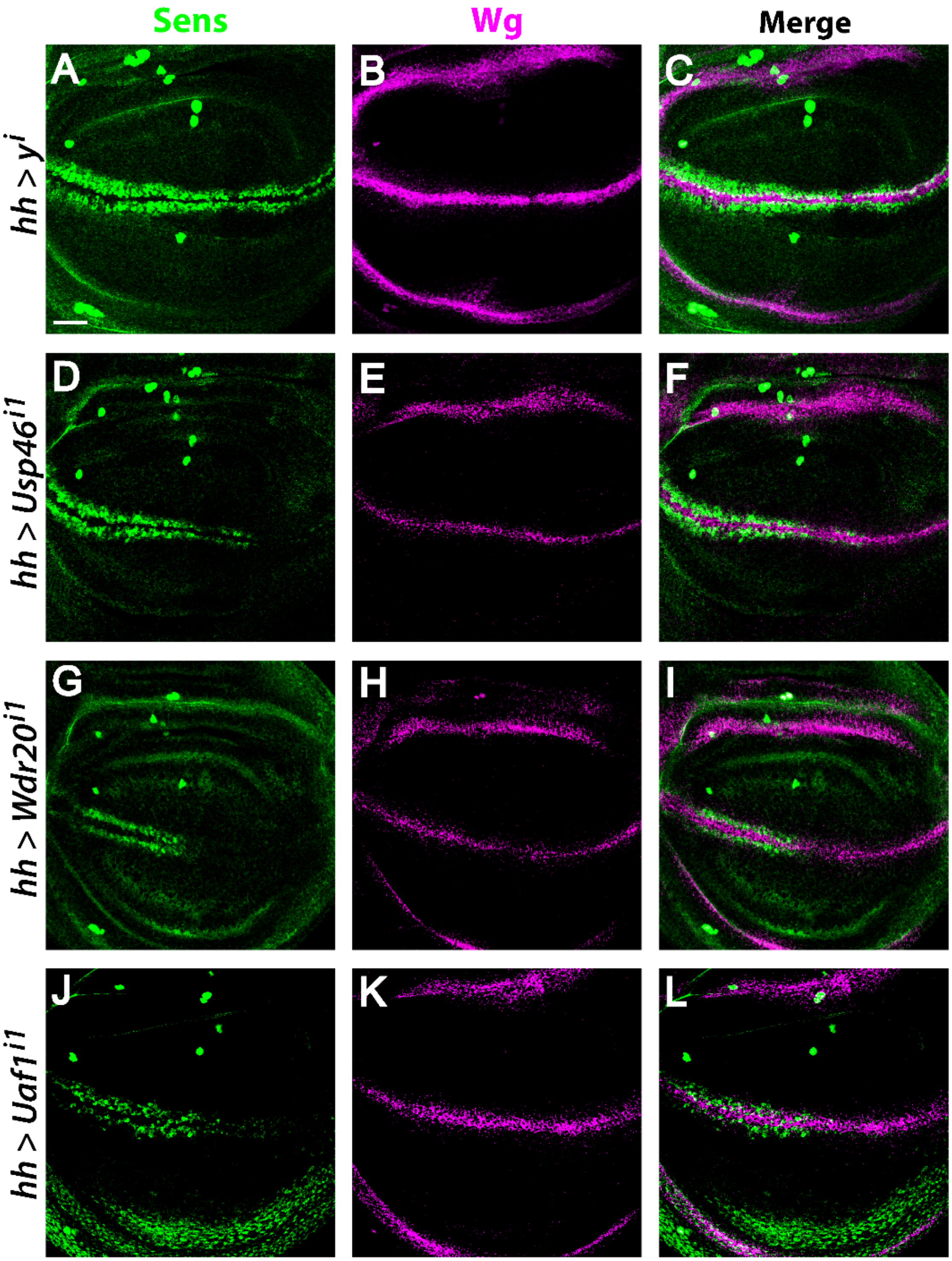

Figure S3. The Usp46 complex does not regulate Wingless expression.
(**A-L**) RNAi constructs targeting each Usp46 complex component or the *yellow* negative control were expressed in the posterior compartment of third instar larval wing discs using the *hh-Gal4* driver. Wingless (Wg, magenta), Senseless (Sens, green). Dorsal, top and posterior, right. Scale bar (A-L): 20 μM

(**A-C**) *hh-Gal4*-driven expression of a control RNAi construct targeting the *yellow (y)* gene. No loss of Sens (green) or Wg (magenta) was observed.

(**D-L**) *hh-Gal4*-driven expression of RNAi constructs targeting *Usp46* (**D-F**), *Wdr20* (**G-I**) or *Uaf1* (**J-L**) results in decreased Sens (green) in the posterior compartment, but no change in the level of Wingless (magenta).

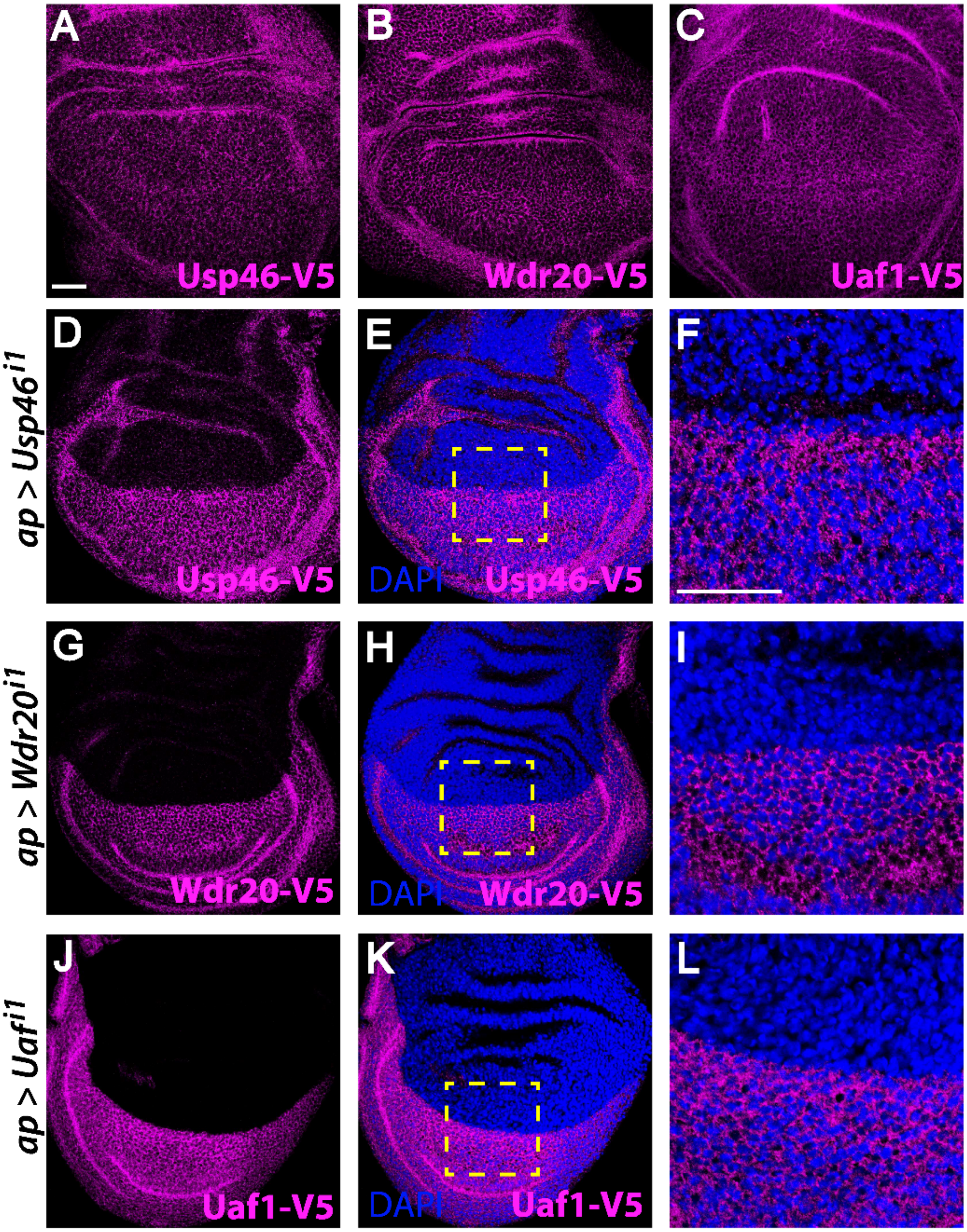

Figure S4. Usp46 complex components are expressed at invariant levels in the larval wing disc.
(**A-C**) Usp46-V5 staining (**A**, magenta), Wdr20-V5 staining (**B**, magenta), and Uaf1-V5 staining (**C**, magenta) in the wing disc are observed at invariant levels. Dorsal, top.

(**D-L**) RNAi-mediated depletion of Usp46 complex components driven by *apterous (ap)-Gal4* in the dorsal compartment of the wing disc. Efficient depletion of Usp46-V5 (**D-E,** magenta), Wdr20-V5 (**G-H**, magenta) or Uaf1-V5 (**J-K**, magenta) is observed in the dorsal compartment, revealing specificity of the V5 signal in the ventral compartment for each Usp46 complex component. DAPI (blue) marks nuclei. Dorsal, top and posterior, right.

(**F, I, L**) Optical zoom of the region indicated in the yellow box (**E, H, K**), respectively, at the dorsoventral boundary of the wing disc. There is nuclear exclusion and cytoplasmic enrichment of Usp46 (magenta, **F**), Wdr20 (magenta, **I**) and Uaf1 (magenta, **L**).

Scale bars (A-E, G, H, J, K) and (F, I, L): 20 μM

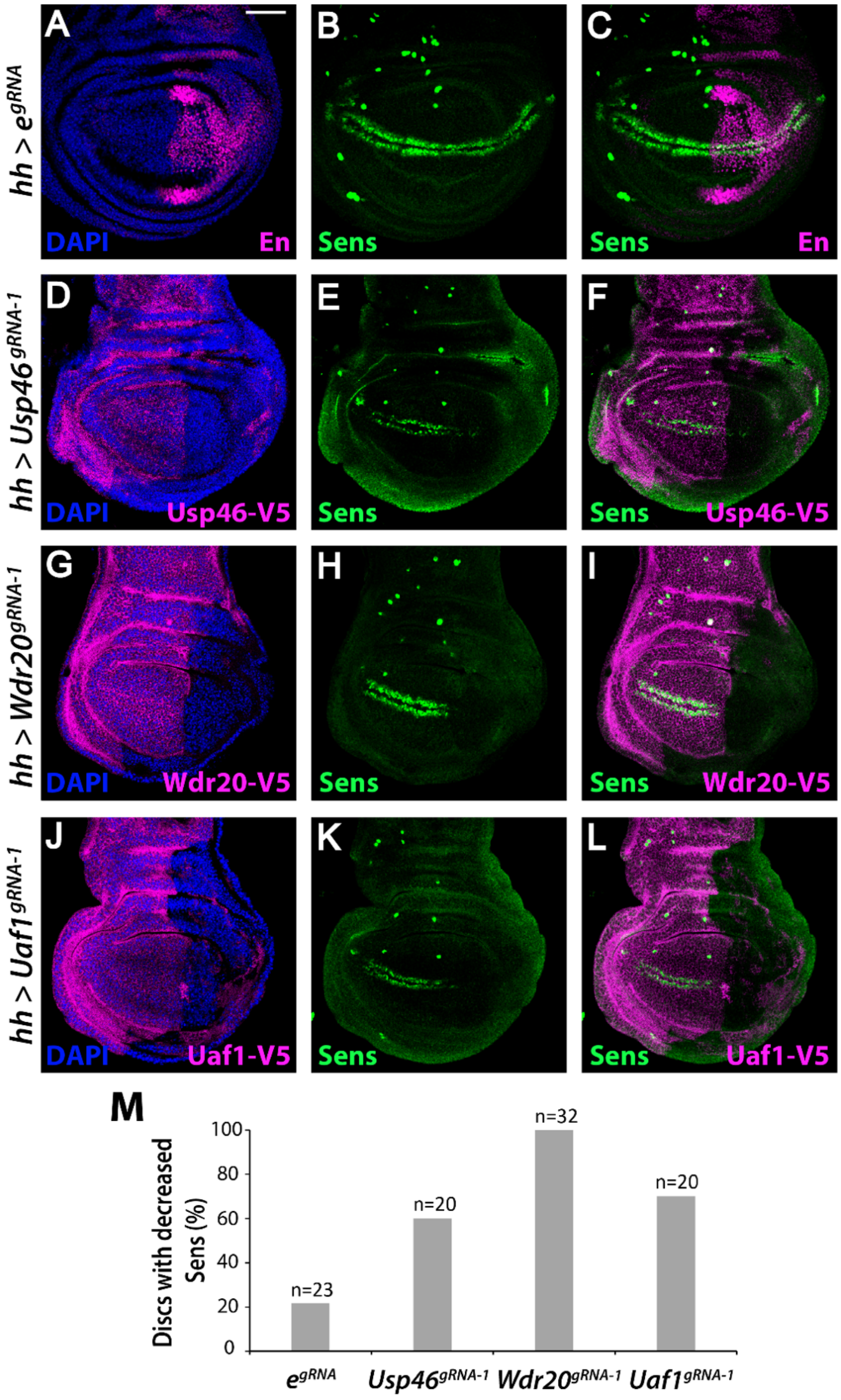

Figure S5. Conditional CRISPR-mediated mutagenesis of Usp46 complex components results in reduced expression of the Wingless target gene *senseless* in the wing disc.
(**A-C**) Cas9 and control sgRNAs targeting *ebony (e)* expressed in the posterior compartment of the wing disc using the *hh-Gal4 (hh)* driver have little effect on Sens (green) levels. The posterior of the wing disc is marked with Engrailed (En, magenta). DAPI (blue) marks nuclei.

(**D-L**) Cas 9 and sgRNAs targeting each component of the Usp46 complex were expressed in the posterior compartment of the wing disc using the *hh-Gal4* driver. CRISPR-mediated mutagenesis resulting in decreased levels of V5-tagged Usp46 (**D-F**), V5-tagged Wdr20 (**G-I**) and V5-tagged Uaf1 (**J-L**) is indicated by decreased V5 staining (magenta) in the posterior disc. Decreases in Sens levels (green) indicate the loss of Wingless signaling, which is observed only in the posterior compartment where Cas9 and the sgRNAs are both expressed. DAPI (blue) marks nuclei. Scale bar (A-L): 50 μM. Dorsal, top and posterior, right.

(**M**) Quantification is shown as percentage of discs from males of each genotype with decreased Sens. N is the number of discs analyzed. Source data are provided in the Source Data file.

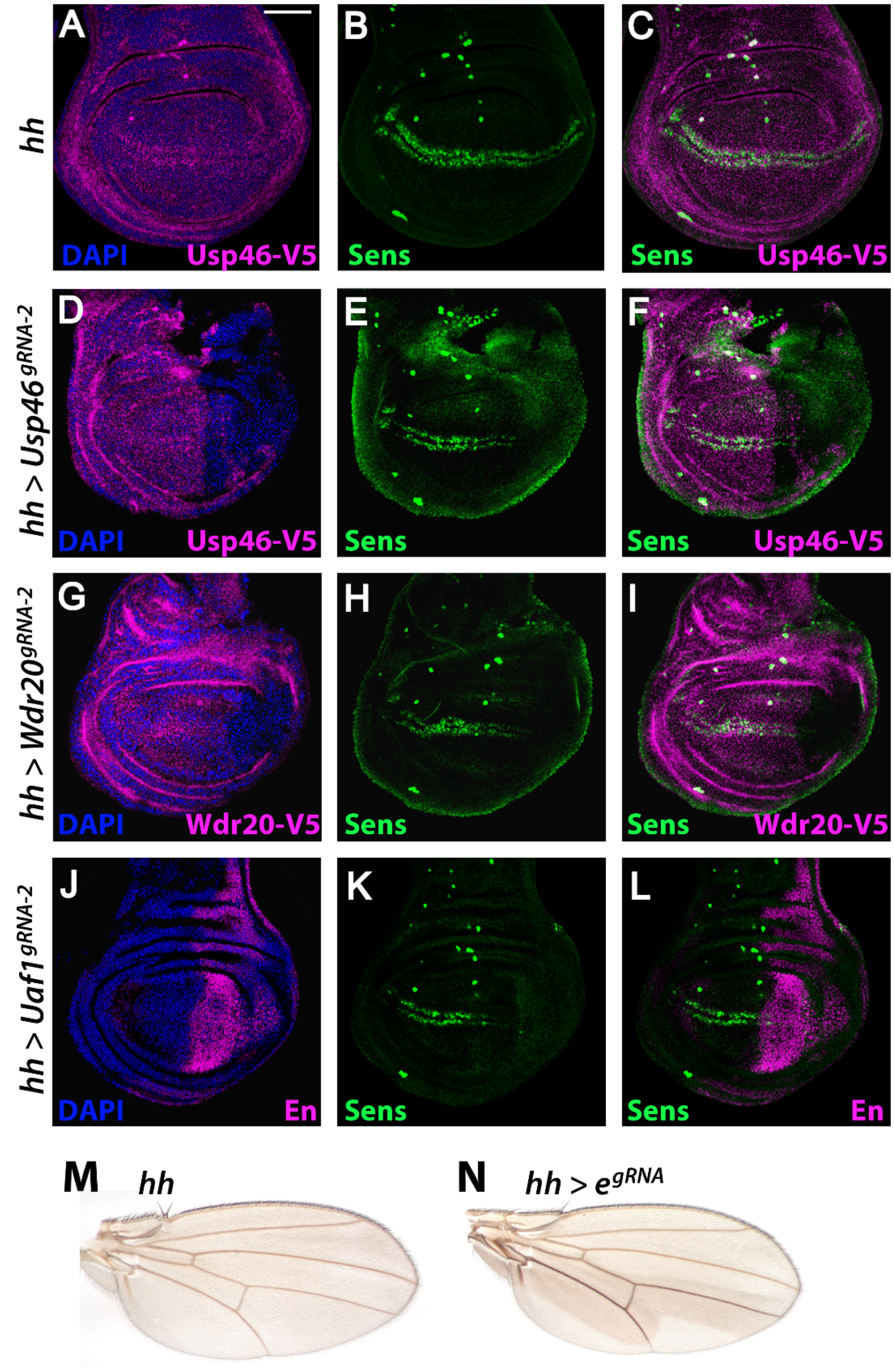

Figure S6. Independent sgRNAs targeting Usp46 complex components result in decreased expression of the Wingless target gene *senseless*.
(**A-I**) Expression of Cas9 only (**A-C**), or together with independent sgRNAs targeting *Usp46* (**D-F**) or *Wdr20* (**G-I**) in the posterior compartment of the wing disc using the *hh-Gal4* driver resulted in decreased Senseless (Sens, green) in the posterior disc. Decreased V5 staining (magenta) indicates cells with reduced Usp46 or Wdr20, respectively. DAPI (blue) marks nuclei.

(**J-L**) Cas9 and independent sgRNAs targeting *Uaf1* expressed in the posterior compartment of the wing disc using *hh-Gal4* result in decreased Sens staining (green) in the posterior compartment, marked with Engrailed (En, magenta). DAPI (blue) marks nuclei. Scale bar (A-L): 50 μM

(**M, N**) Cas9 expression driven by *hh-Gal4* has no phenotype in the adult wing in the absence of sgRNAs (**M**), whereas concomitant expression of *ebony* sgRNAs results in an *ebony* phenotype in the posterior wing (**N**). Posterior, bottom.

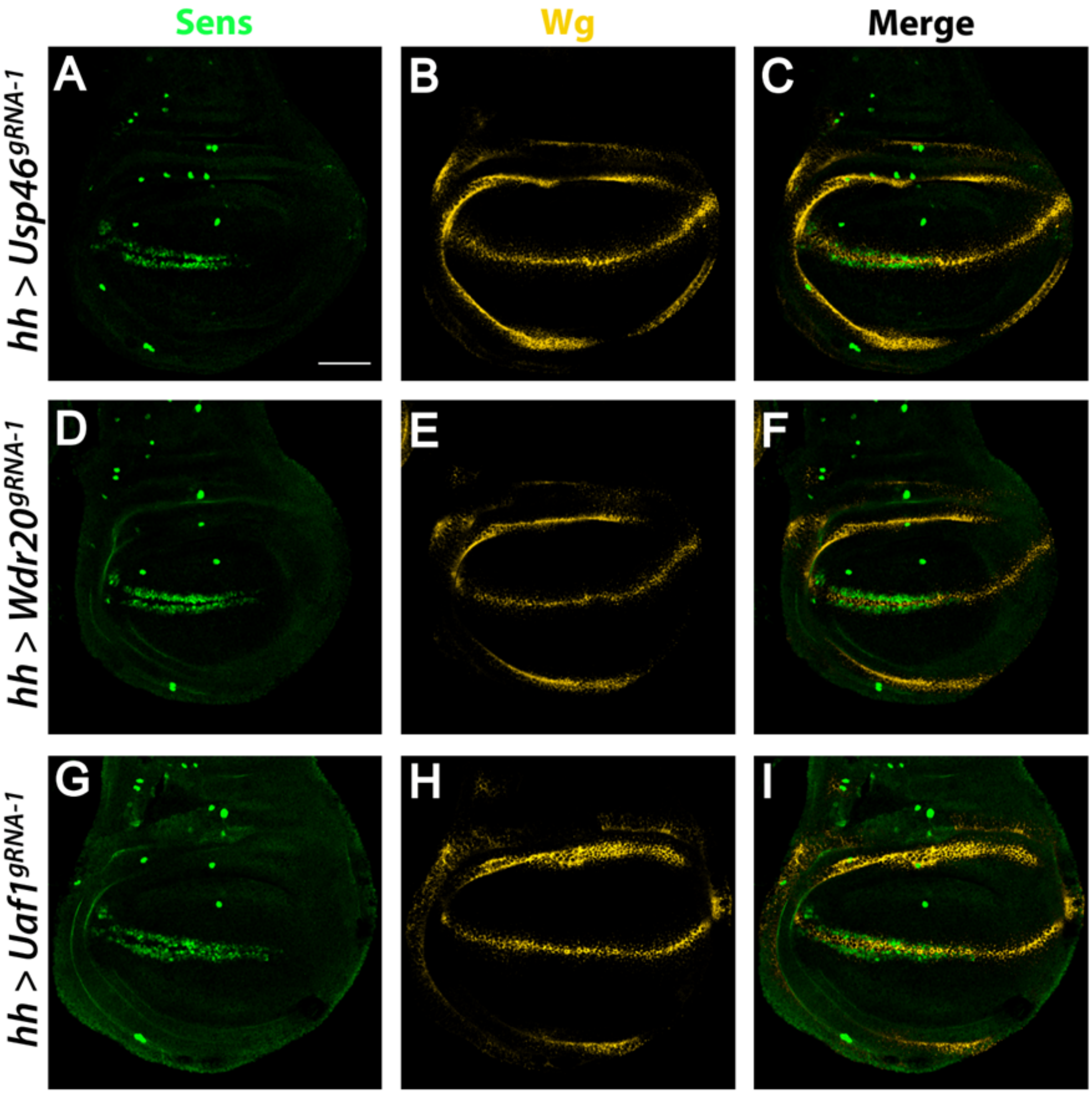

Figure S7. Wingless expression is unaffected by conditional CRISPR-mediated mutagenesis of the Usp46 complex. (**A-I**) Cas9 and sgRNAs targeting *Usp46* (**A-C**), *Wdr20* (**D-F**) or *Uaf1* (**G-I**) were expressed in the posterior compartment of the third instar larval wing disc using the *hh-Gal4* driver. Reduction of Sens (green) was observed specifically in the posterior region of each wing disc examined. In contrast, Wingless (yellow) expression was unaffected.

Scale bar (A-I): 50 µM

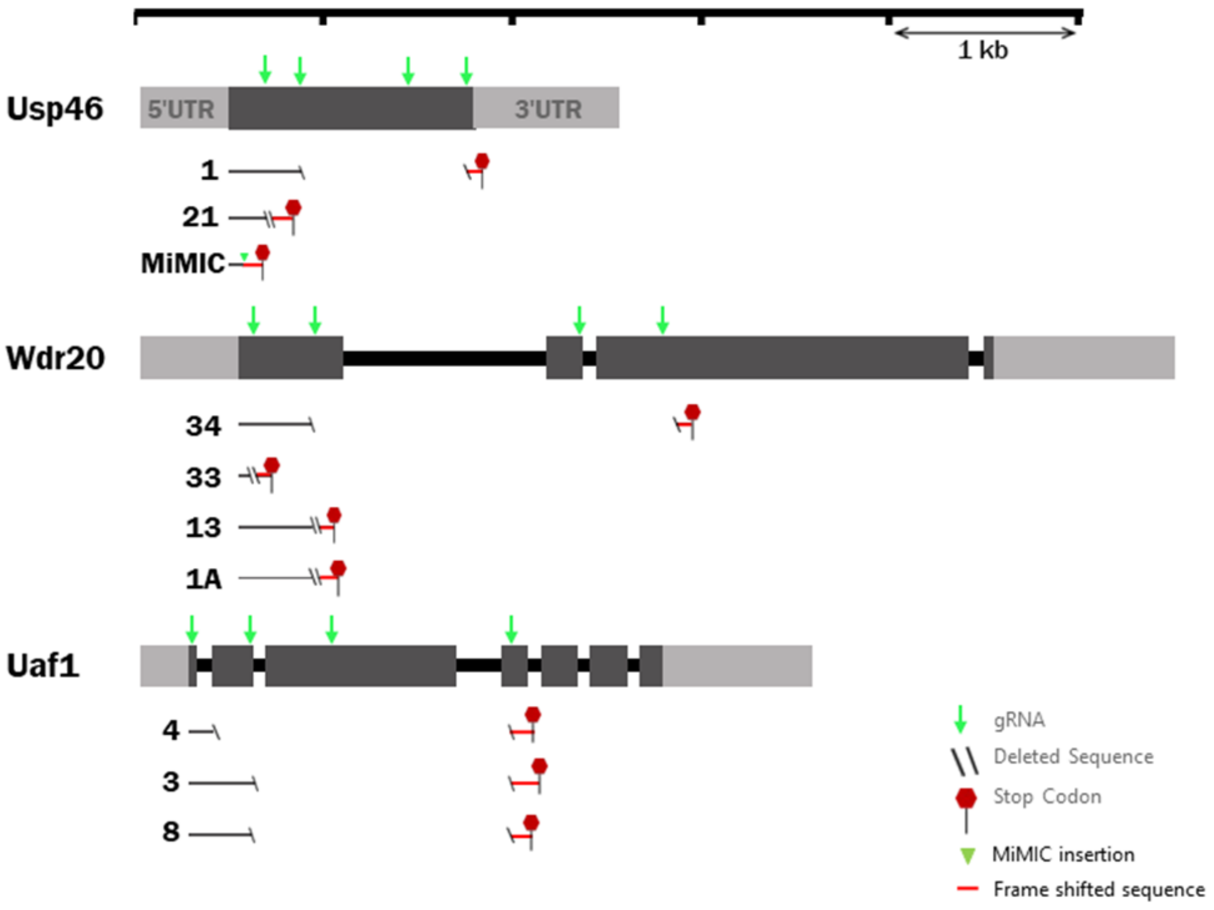

Figure S8. Schematic representation of CRISPR/Cas9-generated mutant alleles of the Usp46 complex. Genomic regions for *Usp46*, *Wdr20* and *Uaf1*, and deletions or truncations in mutant alleles are shown. Intron (black), exons (dark gray), and untranslated regions (UTRs, light gray) are indicated. For mutant alleles, line breaks indicate position of deletion, and stop sign indicates premature stop codons caused by frame shift mutations that result in truncations. The $Usp46^{MiMIC}$ allele contains an insertion of a *Minos*-mediated integration cassette (MiMIC) in the *Usp46* gene. The sites targeted by gRNAs are denoted by green arrows. Genomic scale is shown on top.

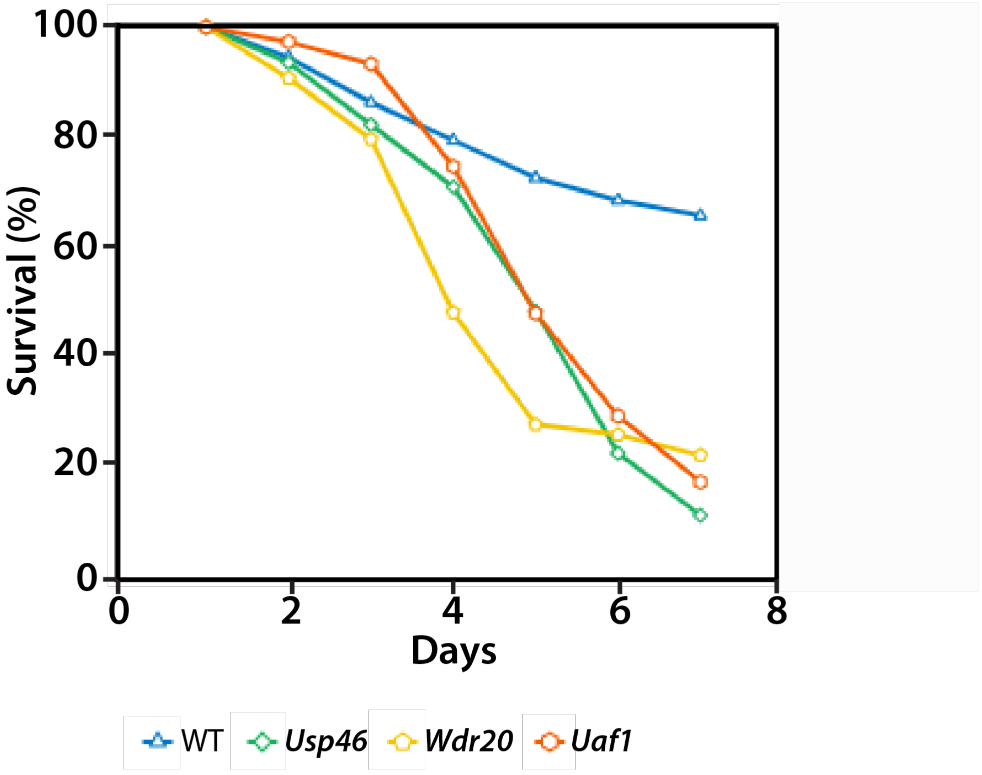

Genotype	No. of subjects	P value
WT	73	
Usp46	62	9.8E-9
Wdr20	54	1.2E-6
Uaf1	75	2.6E-7

Figure S9. *Usp46*, *Wdr20, and Uaf1* mutants have increased mortality.
Usp46$^{1/MiMIC}$ (green), *Wdr20*$^{33/34}$ (yellow), and *Uaf1*$^{3/8}$ (orange) null transheterozygous flies reared on 5% sucrose displayed reduced survival when compared to wild-type (WT, blue). Number of subjects and p values are shown. Source data are provided in the Source Data file.

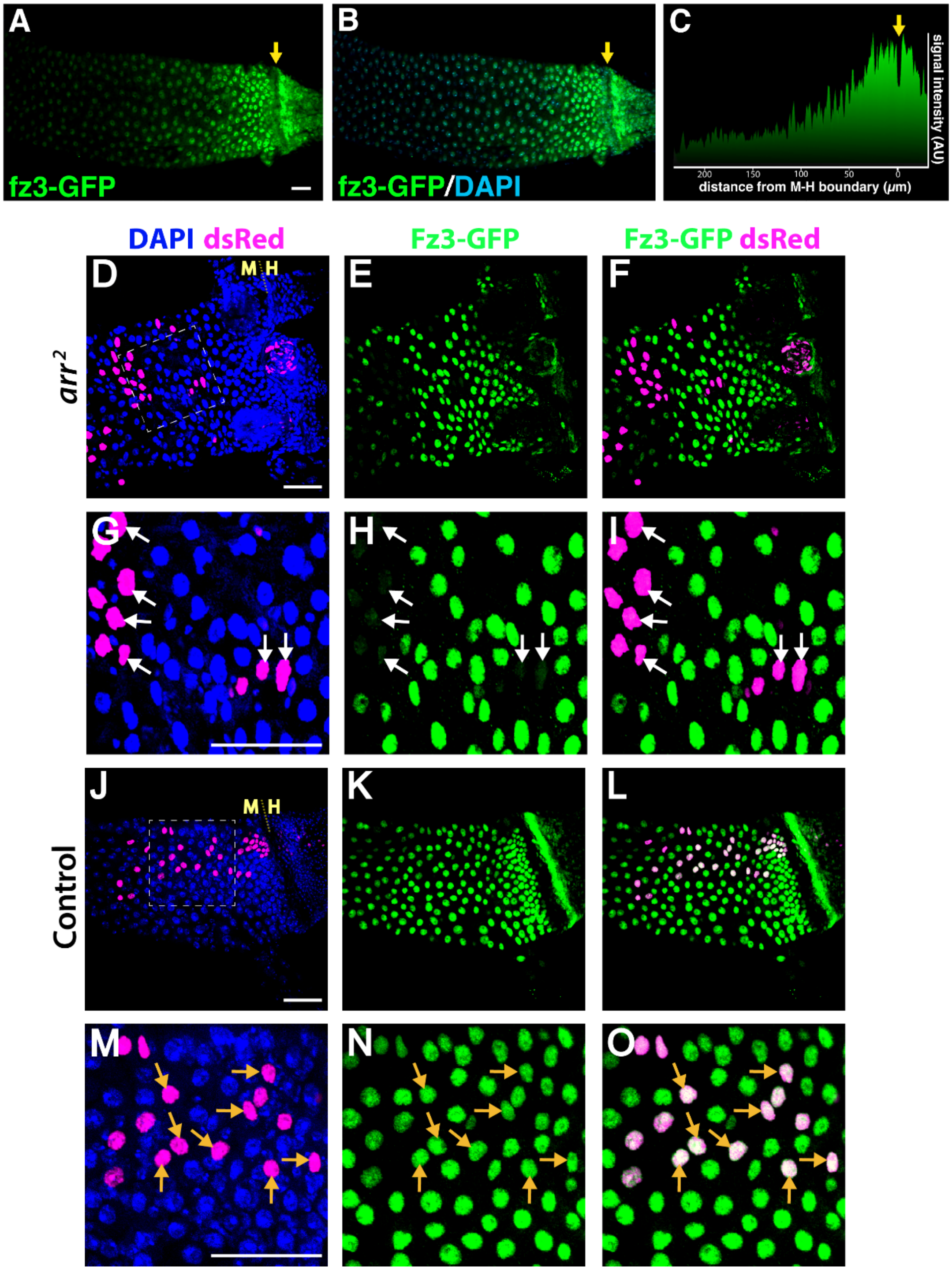

Figure S10. Arrow promotes expression of the Wingless target gene *frizzled3* (*fz3)* in the posterior midgut.
(**A-C**) Expression of *fz3-GFP* (green) in the wild-type adult posterior midgut (**A, B**). Quantification of *fz3-GFP* signal intensity as a function of distance from the midgut-hindgut boundary (MHB, yellow arrow) (**C**). Posterior, right.

(**D-F**) *arrow* null mutant clones (magenta) in the adult intestinal epithelium. *fz3-GFP* expression (green). The MHB is delineated (M|H). Posterior, right. DAPI (blue) marks nuclei.

(**G-I**) Higher magnification of white box in panel D. *arrow* null mutant cells displayed complete loss of *fz3-GFP* (white arrows).

(**J-L**) Control clones of wild-type cells (FRT 82B, magenta) in the adult intestinal epithelium.

(**M-O**) Higher magnification view of white box in panel J. *fz3-GFP* expression is not reduced in control clones (orange arrows).

Scale bars (A-C) 20 μM; (D-F), (G-I), (J-L), and (M-O): 50 μM

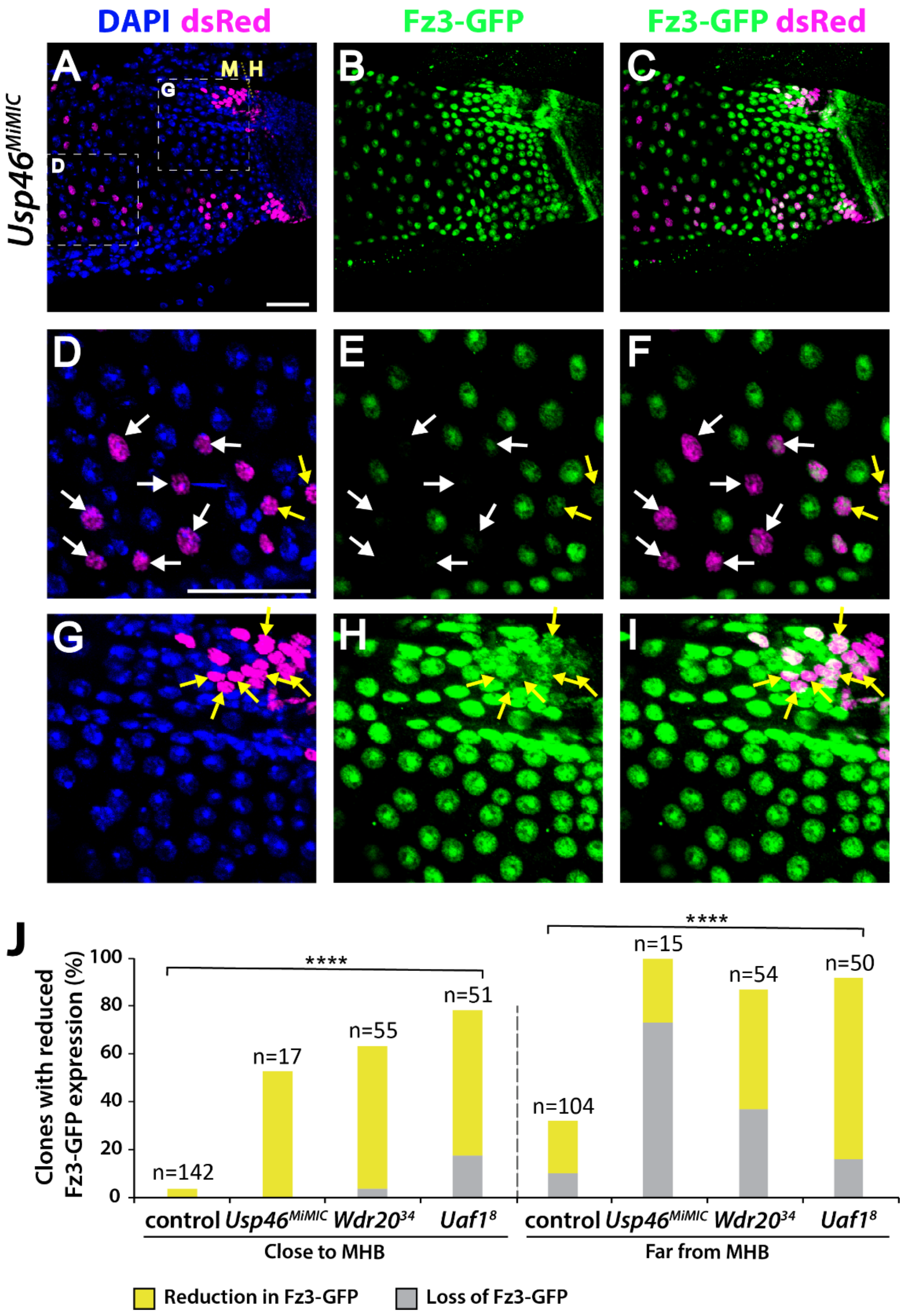

Figure S11. Independent *Usp46, Wdr20* and *Uaf1* null alleles result in reduction of *fz3* expression in the posterior midgut. (**A-C**) *Usp46^{MiMIC}* null mutant clones (magenta) in the adult intestinal epithelium. *fz3-GFP* expression (green). The MHB is delineated (M|H). Posterior, right. DAPI (blue) marks nuclei.

(**D-F**) Higher magnification view of box D in panel A showing a region distant from the MHB. *Usp46^{MiMIC}* null mutant cells in this region showed complete loss of *fz3-GFP* (white arrows) whereas those slightly closer to the MHB showed a nearly complete reduction in *fz3-GFP* (yellow arrows).

(**G-I**) Higher magnification view of box G in panel A showing region near the MHB. *Usp46^{MiMIC}* null mutant cells in this region display either a partial decrease (yellow arrows), or no decrease in *fz3-GFP*. Scale bars (A-C) and (D-I): 50 μM

(**J**) Quantification is shown as percentage of clones of each genotype with decreased *fz3-GFP* (yellow) or the absence of *fz3-GFP* (gray) expression. Clones (n = number) close and far from the MHB were analyzed. ****$p < 0.0001$ (1E-6 for *Usp46^{MiMIC}* clones far from the MHB, 0 for all other genotypes, one-tailed t-test). Source data are provided in the Source Data file.

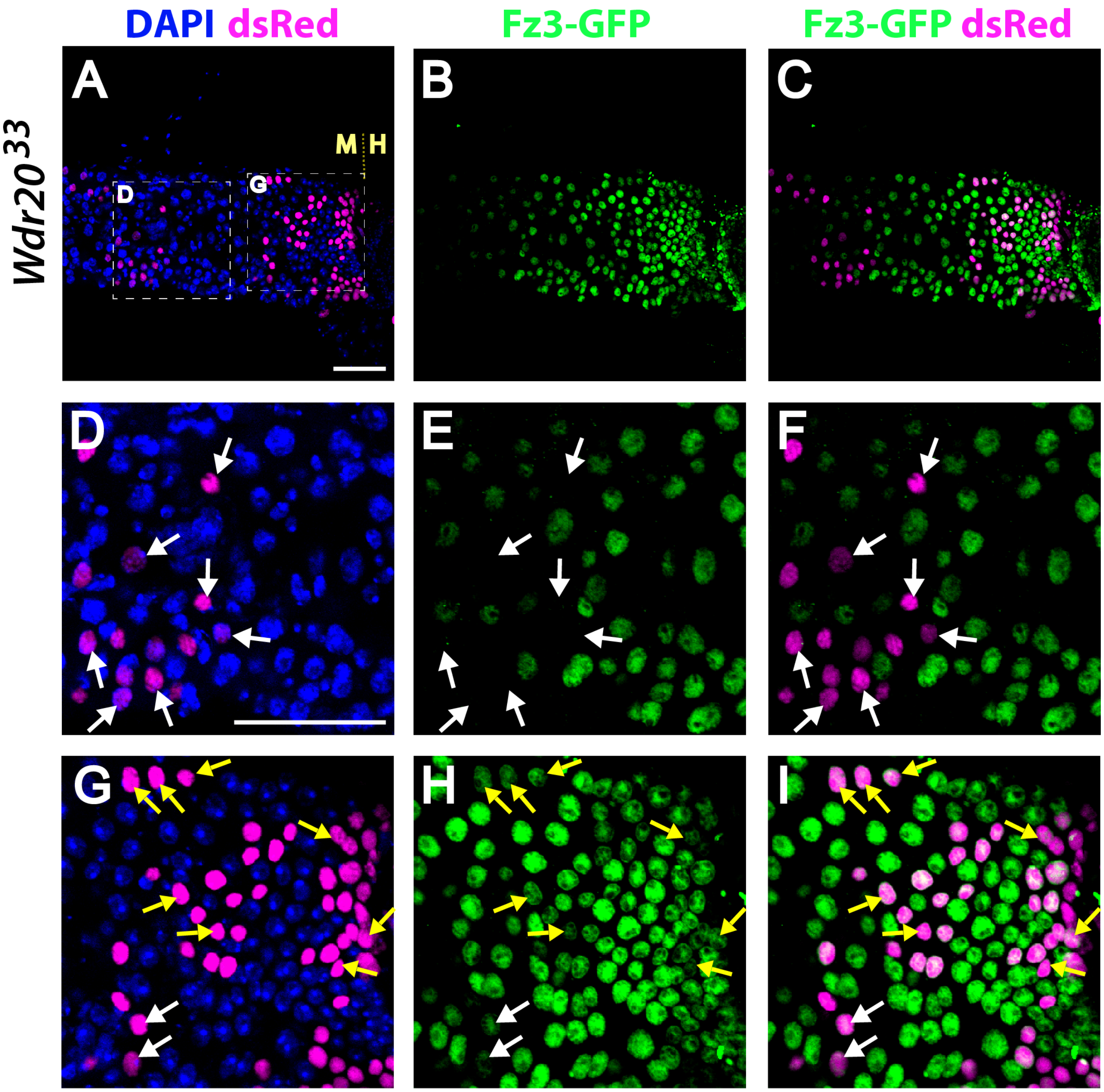

Figure S12. *Wdr20* promotes expression of the Wingless target gene *fz3* in the posterior midgut.
(**A-C**) *Wdr20*^{33} null mutant clones (magenta) in the adult intestinal epithelium. The MHB is delineated (M|H). DAPI (blue) marks nuclei. Posterior, right.

(**D-F**) Magnification of box D from panel A showing a region distant from the MHB. The relative decrease in *fz3-GFP* correlates with distance of the *Wdr20*^{33} mutant clone from the MHB. *Wdr20*^{33} mutant clones (magenta, white arrows) in this region display complete loss of *fz3-GFP*.

(**G-I**) Magnification of box G from panel A showing region near MHB. *Wdr20*^{33} mutant clones located closer to the MHB display a partial reduction in *fz3-GFP* (yellow arrows). A few clones display an almost complete loss of *fz3-GFP* (white arrows).

Scale bars (A-C) and (D-I): 50 μM.

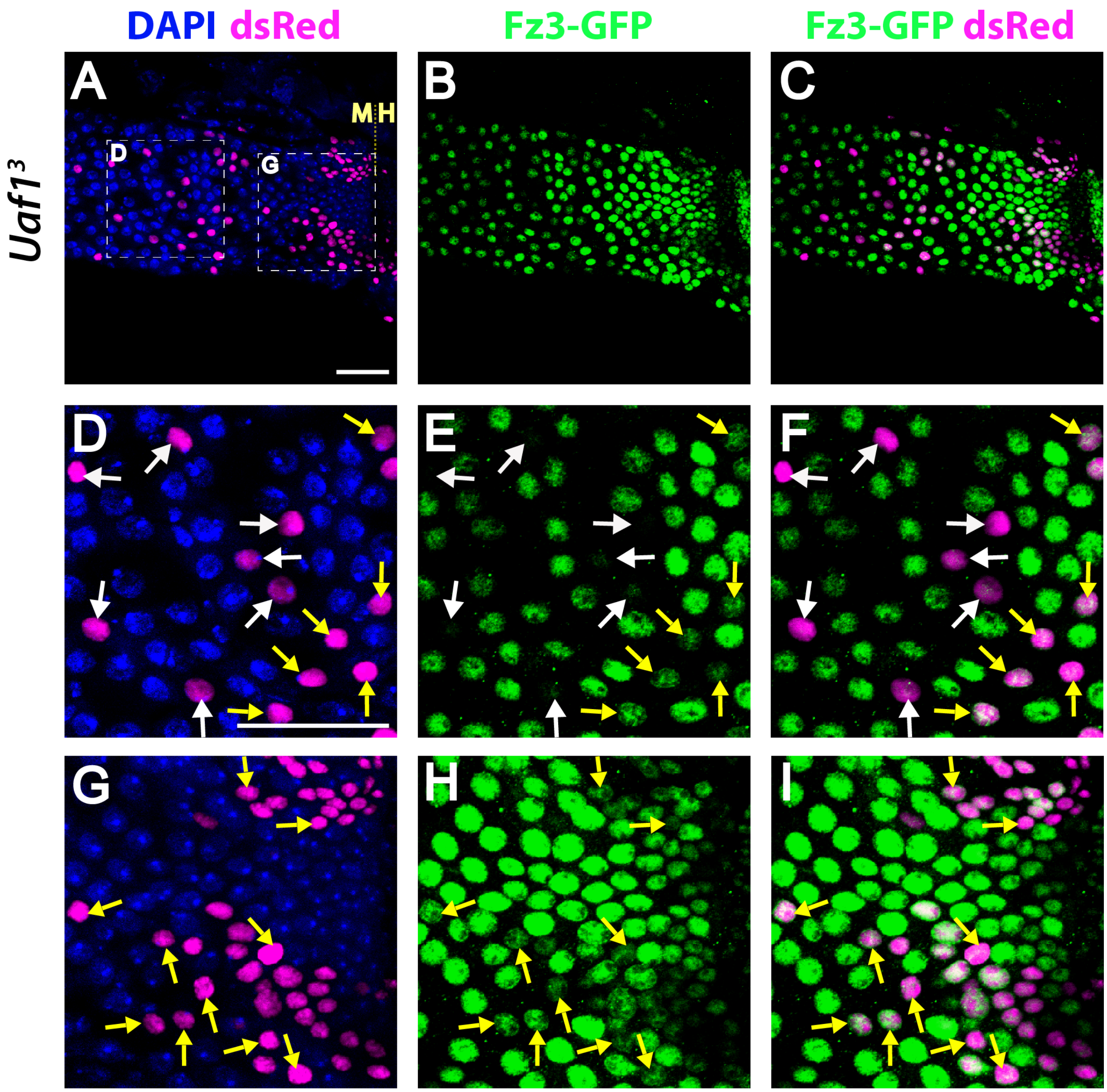

Figure S13. Inactivation of *Uaf1* in the posterior midgut results in decreased expression of the Wingless target gene *fz3*. (**A-C**) *Uaf1*^{3} null mutant clones (magenta) in the adult intestinal epithelium. The MHB is delineated (M|H). DAPI (blue) marks nuclei. Posterior, right.

(**D-F**) Magnification of box D from panel A showing a region distant from the MHB. *Uaf1*^{3} mutant clones (magenta) in this region display complete loss of *fz3-GFP* (white arrows) or strong reduction of *fz3-GFP* (yellow arrows).

(**G-I**) Magnification of box G from panel A showing region near MHB. *Uaf1*^{3} null mutant clones located in this region display a partial reduction in *fz3-GFP* (yellow arrows).

Scale bars (A-C) and (D-I): 50 µM.

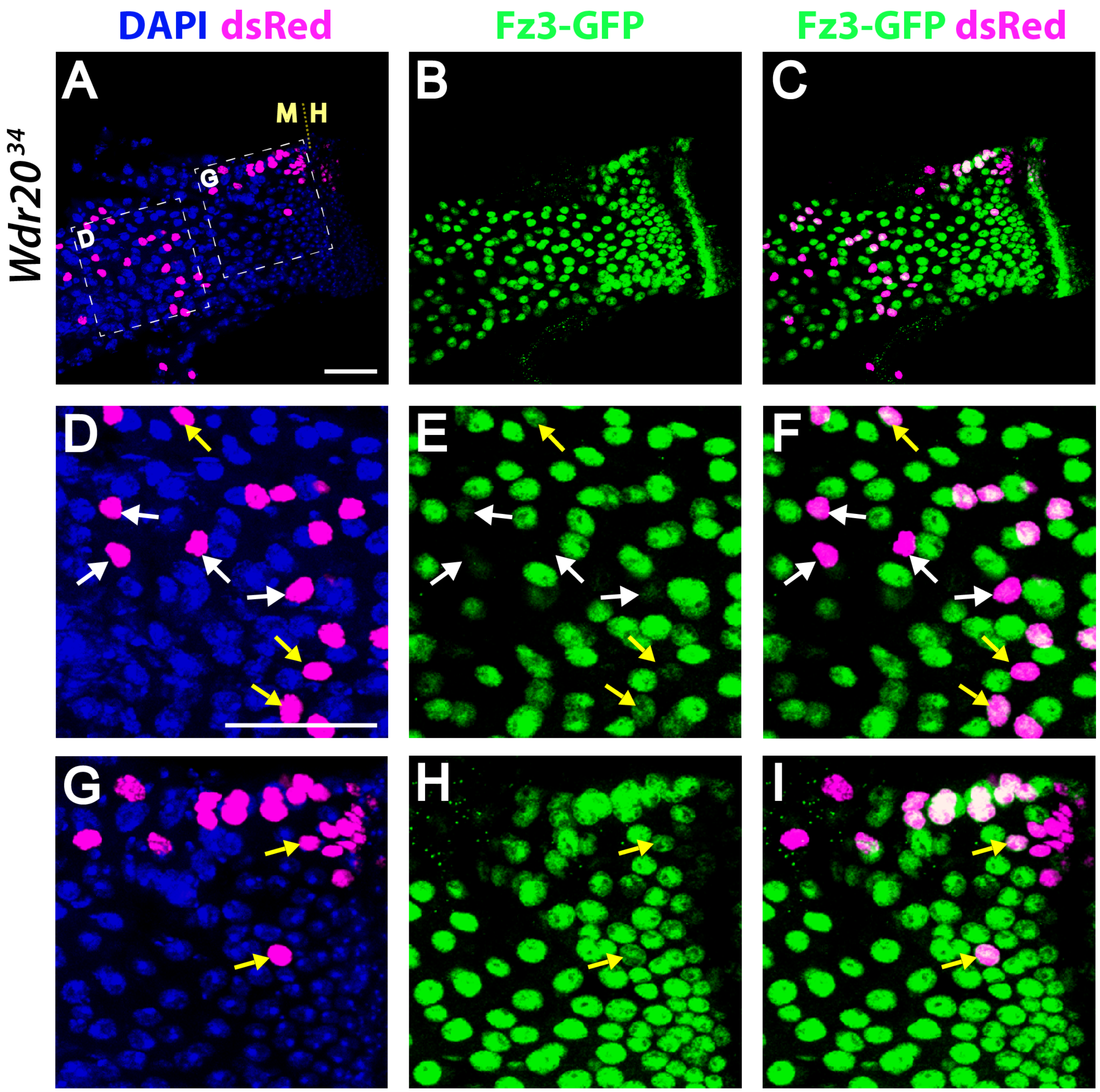

Figure S14. Independent *Wdr20* mutant reduces *fz3* expression in the posterior midgut.
(**A-C**) *Wdr20*^{34} null mutant clones (magenta) in the adult intestinal epithelium. *fz3-GFP* expression (green). DAPI (blue) marks the nuclei. The MHB is delineated (M|H). Posterior, right.

(**D-F**) Magnification of box D from panel A showing a region distant from the MHB. *Wdr20*^{34} mutant clones (magenta) display complete loss of *fz3-GFP* (white arrows) or strong reduction in in *fz3-GFP* (yellow arrows).

(**G-I**) Magnification of box G from panel A showing *Wdr20*^{34} clones (magenta) closer to the MHB, which display a partial reduction in *fz3-GFP* (yellow arrows).

Scale bars: (A-C) and (D-I): 50 μM

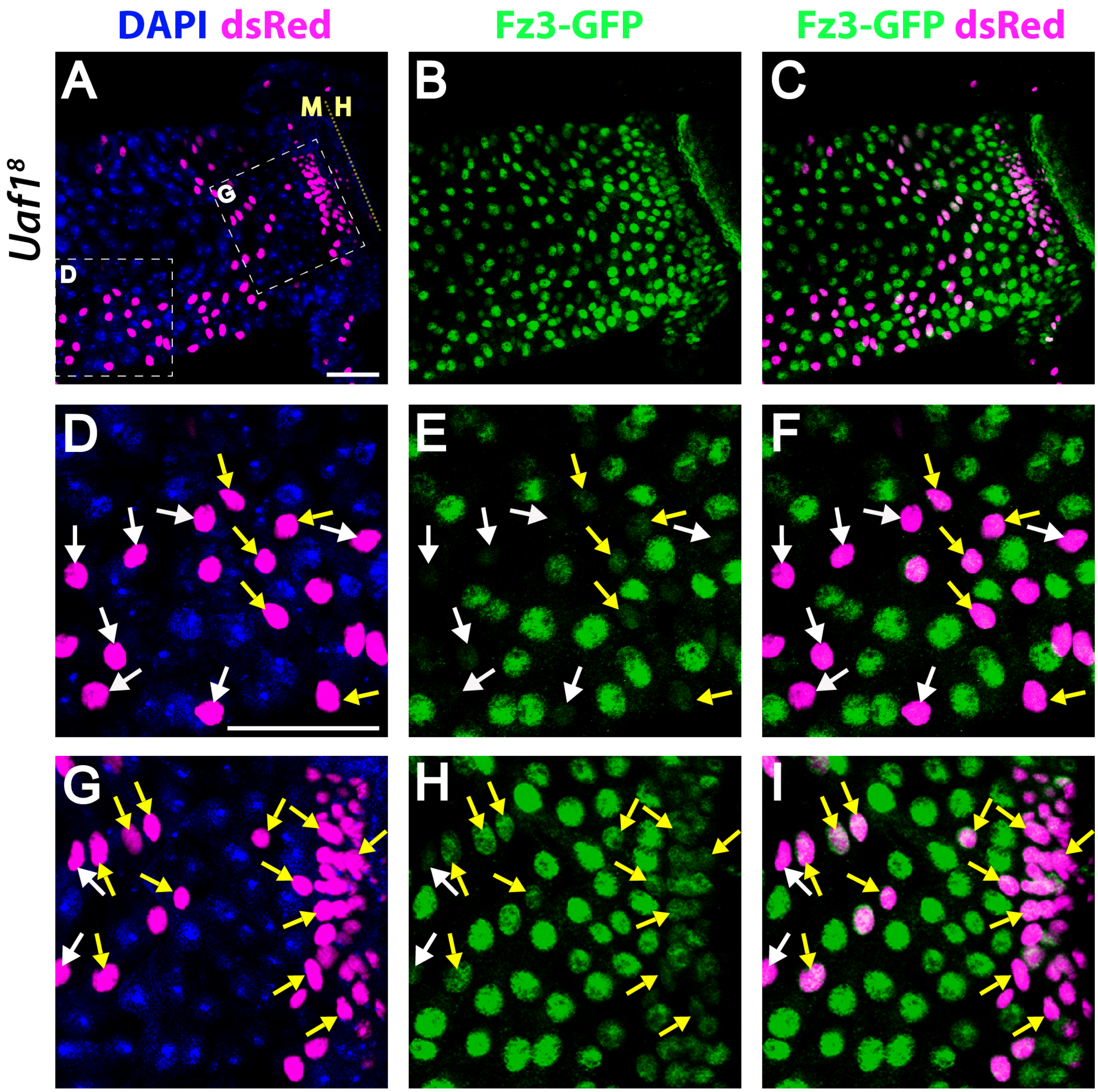

Figure S15. Independent *Uaf1* null mutation results in reduced *fz3* expression in the posterior midgut.
(**A-C**) *Uaf1*^{8} null mutant clones (magenta) in the adult intestinal epithelium. The MHB is delineated (M|H). DAPI (blue) marks nuclei. Posterior, right.

(**D-F**) Magnification of box D from panel A showing a region distant from the MHB. *Uaf1*^{8} mutant clones (magenta) in this region display complete loss of *fz3-GFP* (white arrows) or strong reduction of *fz3-GFP* (yellow arrows).

(**G-I**) Magnification of box G from panel A showing a region near the MHB. *Uaf1*^{8} null mutant clones in this region display a partial reduction in *fz3-GFP* (yellow arrows). A few clones display an almost complete loss of *fz3-GFP* (white arrows).

Scale bars (A-C) and (D-I): 50 µM.

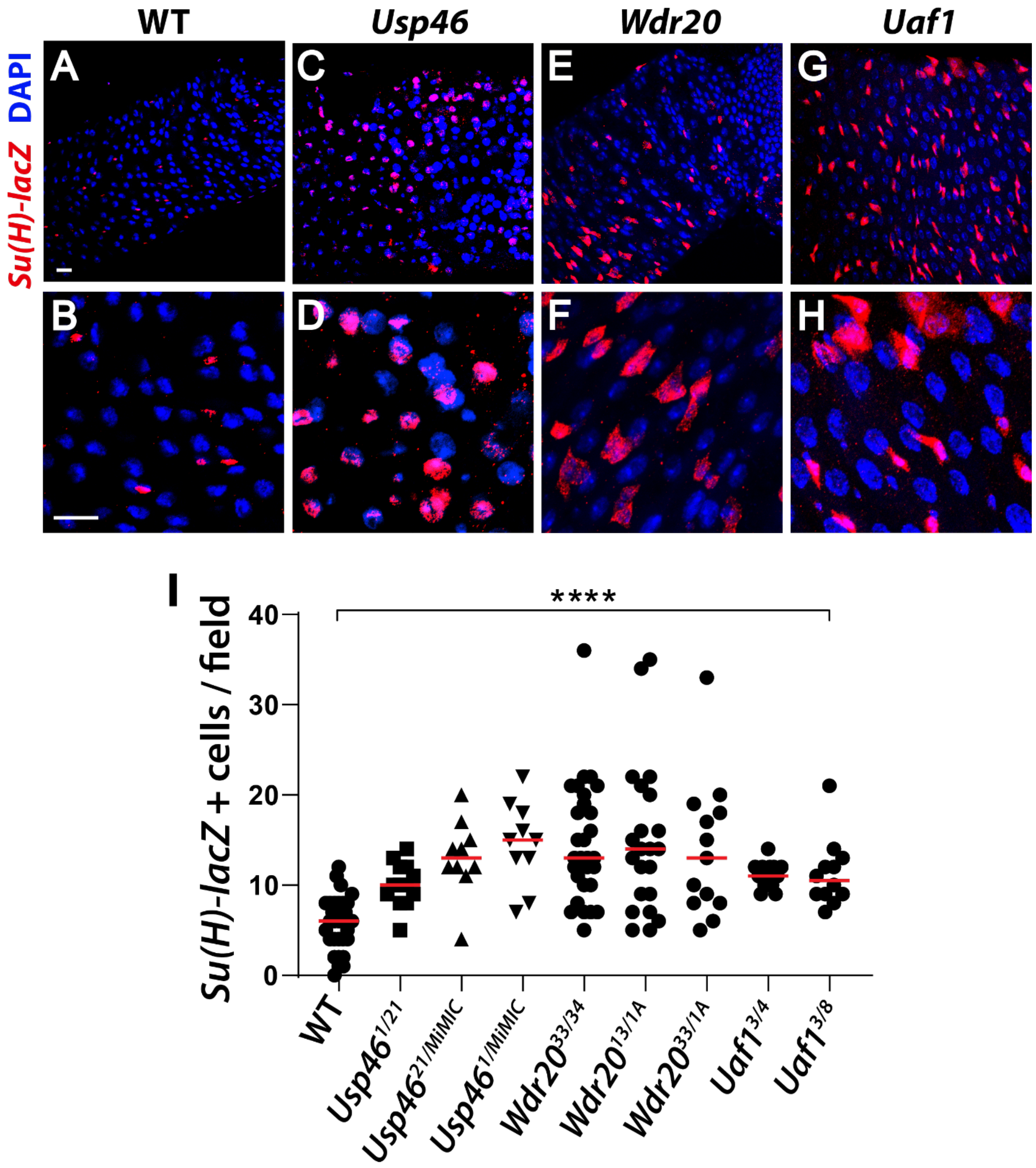

Figure S16. Loss of the Usp46 complex does not block the asymmetric division of ISCs.
(**A-H**) The enteroblast-specific transcriptional marker *Su(H)-lacZ* (red) marks progenitor cells derived from the asymmetric division of intestinal stem cells. Midguts from *Usp46*, *Wdr20* or *Uaf1* null allele transheterozygotes displayed an increased number of enteroblasts when compared to wild-type midguts. Panels B, D, F, and H are higher magnification views of panels A, C, E, and G, respectively. DAPI (blue) marks the nuclei. Scale bars (A,C,E,G) and (B,D,F,H): 20 μM

(**I**) Quantification of *Su(H)-lacZ* positive cells is shown as mean (red line). 0.051mm^2 fields in either the R5 region adjacent the MHB or at the R4/R5 boundary were analyzed. Each point represents an individual posterior midgut. ****p < 0.0001 (p values in order: 3.4E-5, 2.9E-7, 1E-8, <1E-8, 4.8E-7, 5.67E-6, 1E-7 and 8.4E-6, two tailed t-test). Source data are provided in the Source Data file.

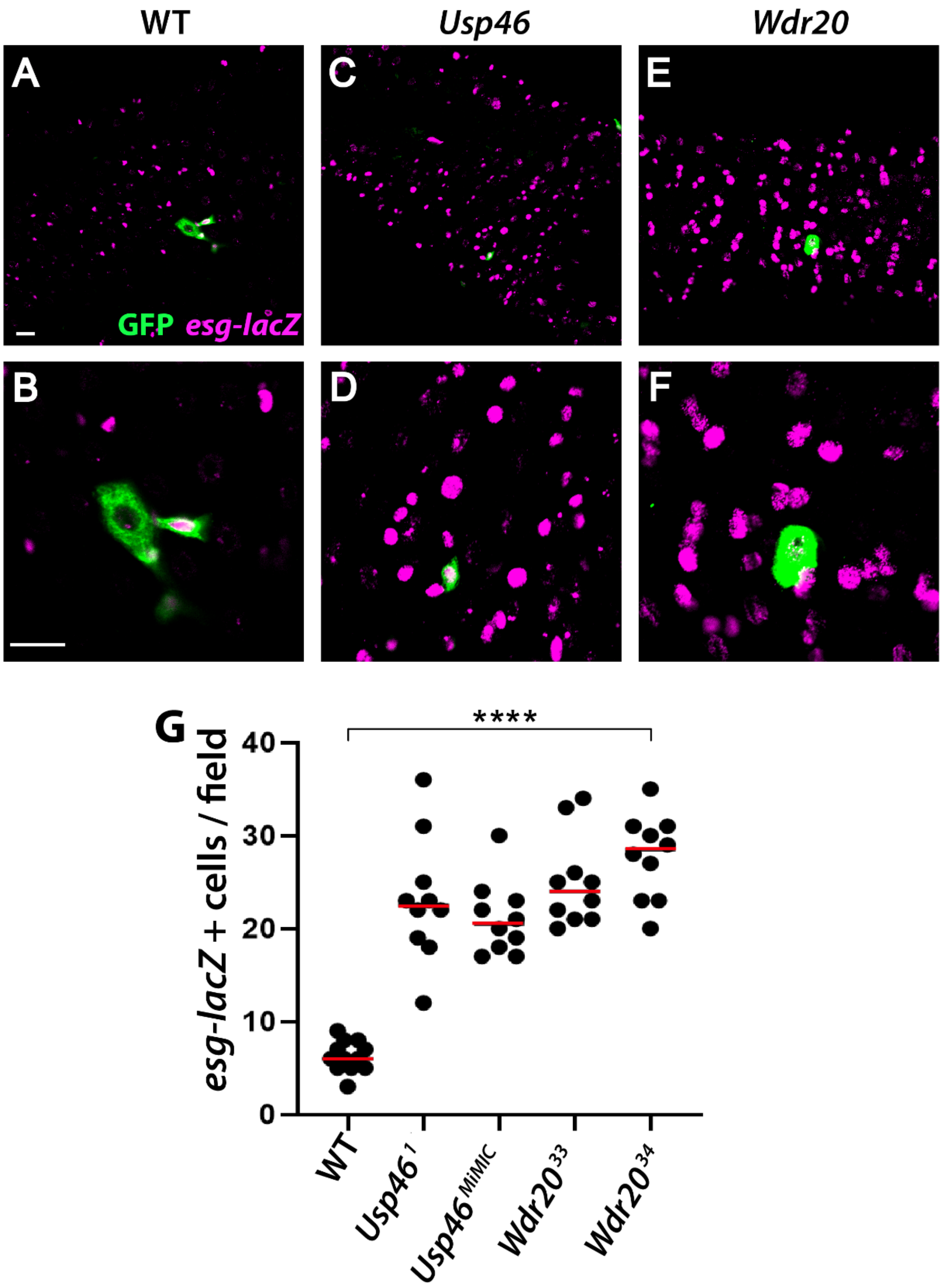

Figure S17. Usp46 and Wdr20 prevent non-autonomous overproliferation of neighboring ISCs.
(**A, B**) Control clones (*FRT82B*) of wild-type (WT) cells marked with GFP (green) were generated using the MARCM system in the wild-type adult midgut epithelium. *esg-lacZ* (magenta). Panel B is a higher magnification view of panel A.

(**C-F**) *Usp46* and *Wdr20* null mutant clones (marked with GFP, green) induce non-autonomous regional increases in the number of intestinal stem and progenitor cells (marked by *esg-lac*Z, magenta). Higher magnification views of panels C and E are shown in panels D and F, respectively. Scale bars (A, C, E) and (B, D, F): 20 µM

(**G**) Quantification of *esg-lacZ* positive cells is shown as mean (red line). Each point represents an individual posterior midgut. 0.051mm^2 fields in the R5 region were analyzed. ****p < 0.0001 (5E-8 for *Usp46*^{1} and <1E-8 for all other genotypes, two-tailed t-test). Source data are provided in the Source Data file.

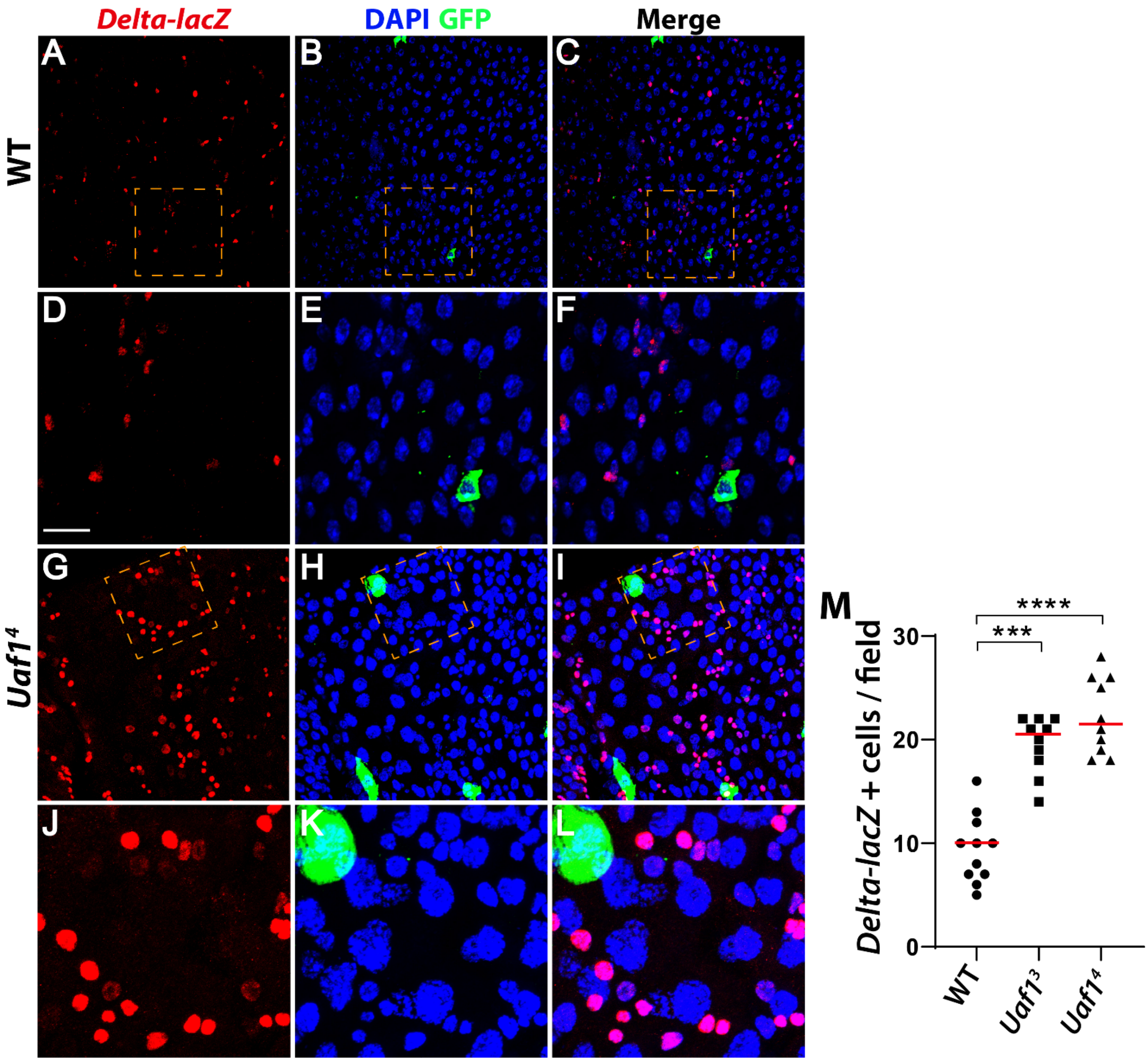

Figure S18. Uaf1 prevents the non-autonomous overproliferation of neighboring ISCs.
(**A-F**) Control clones (*FRT42D*) of wild-type (WT) cells marked with GFP (green) were generated using the MARCM system in the wild-type adult midgut epithelium. *Delta-lacZ* (red) marks intestinal stem cells and DAPI (blue) marks the nuclei. Panels D, E, and F are higher magnification views of the boxed region in panels A, B and C, respectively.

(**G-L**) *Uaf1*^{4} null mutant clones (marked with GFP, green) induce the non-autonomous regional overproliferation of intestinal stem cells (marked by *Delta-lacZ*, red). DAPI (blue) marks the nuclei. Panels J, K, and L are higher magnification views of the boxed area in panels G, H, and I, respectively. Scale bars (A-C, G-I) and (D-F, J-L): 20 μM

(**M**) Quantification of *Delta-lacZ* positive cells is shown as mean (red line). Each point represents an individual posterior midgut. 0.051mm^{2} fields in the R5 region were analyzed. ****p < 0.0001 (4.1E-7 and 8E-8, two-tailed t-test). Source data are provided in the Source Data file.

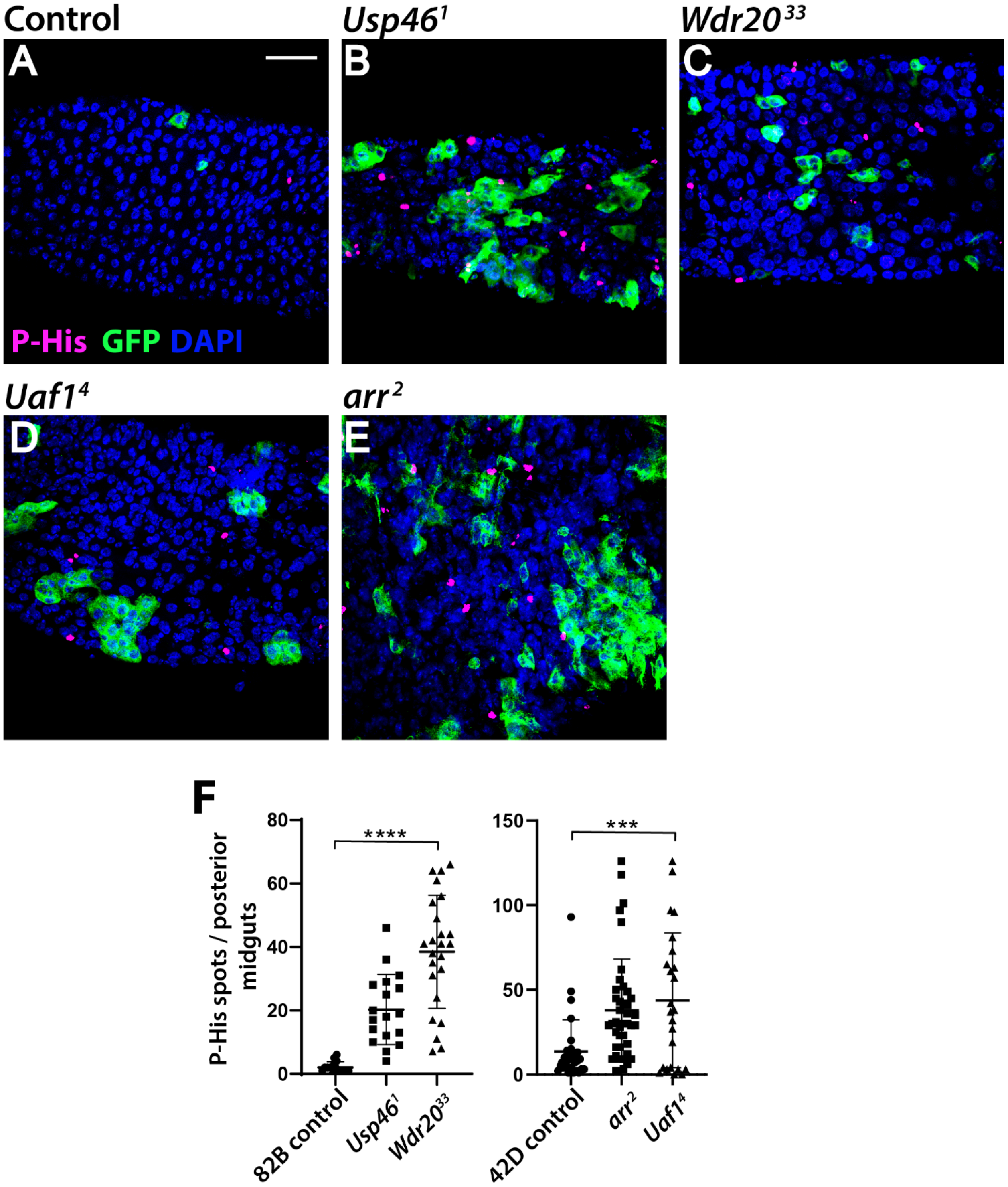

Figure S19. The Usp46 complex regulates cell proliferation in the adult midgut.

(**A**) Control clones (*FRT42D* or *FRT82B* as indicated) of wild-type cells marked with GFP (green) were generated using the MARCM system. Phospho-histone H3 staining in dividing cells is marked in magenta. DAPI (blue) marks the nuclei.

(**B-E**) The number of dividing cells, marked with phospho-histone H3, is increased in midguts with *Usp46* (**B**), *Wdr20* (**C**) and *Uaf1* (**D**) mutant clones, as well as in midguts with *arrow* mutant clones (**E**). Scale bar (A-E): 50 µM.

(**F**) Quantification of phospho-histone H3 in posterior midguts for indicated genotype. Each point represents an individual posterior midgut. Mean and standard deviations are shown. ****p < 0.0001 (5E-7 and <1E-8 for $Usp46^1$ and $Wdr20^{33}$, respectively), ***p < 0.001 (1.96E-4 and 4.62E-4 for arr^2 and $Uaf1^4$, respectively, two-tailed t-test). Source data are provided in the Source Data file.

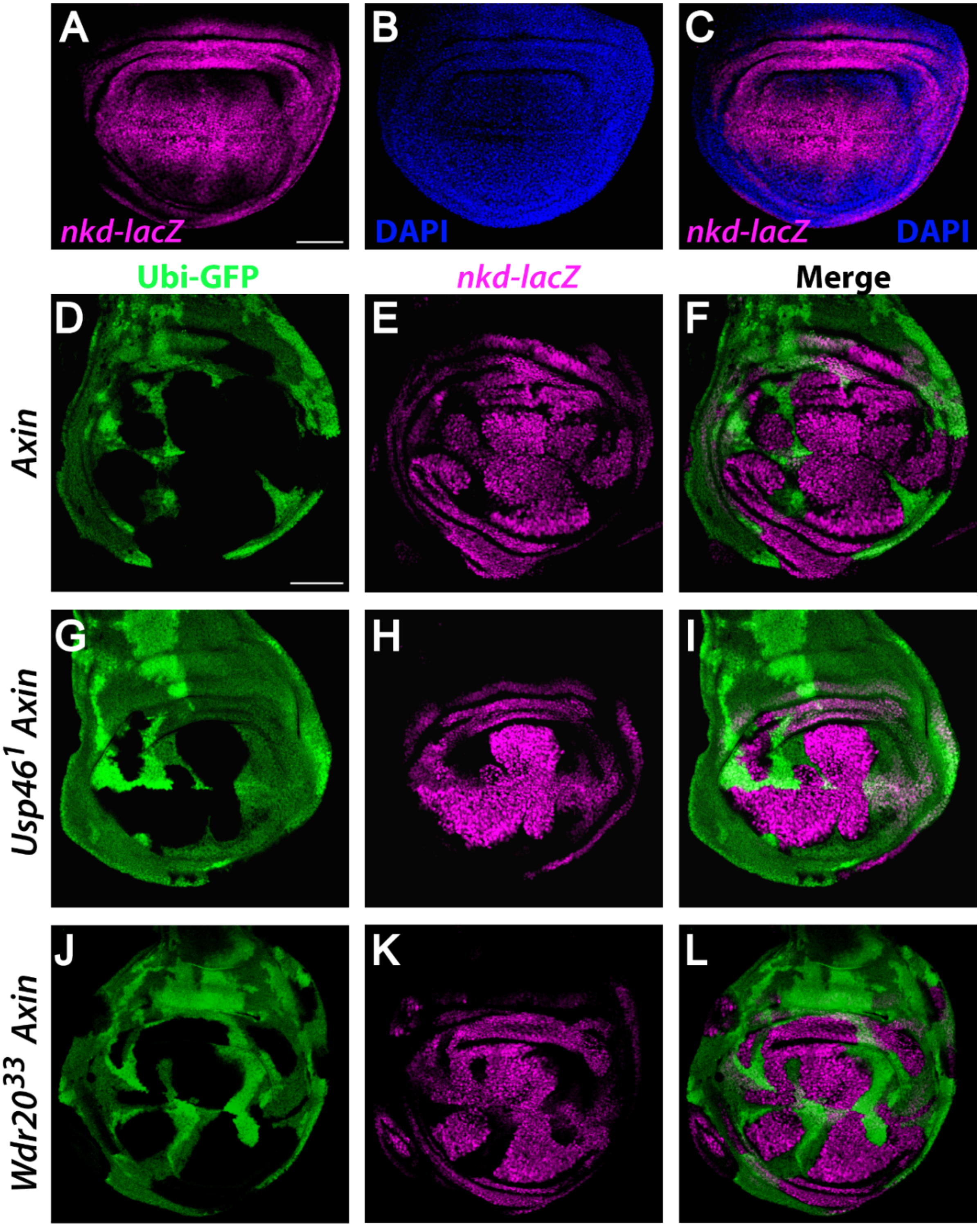

Figure S20. The Usp46 complex acts upstream of the destruction complex.
(**A-C**) Expression of the Wingless target gene reporter *naked-lacZ* (*nkd-lacZ*, magenta) in a wild-type third instar larval wing disc. *nkd-lacZ* expression is observed in a broad band surrounding the dorsoventral boundary. DAPI (blue) marks the nuclei. Dorsal, top.

(**D-F**) *Axin* null mutant clones (marked by the absence of GFP, green) in the wing disc display ectopic expression of *nkd-lacZ* (magenta), indicating the constitutive activation of Wingless signaling.

(**G-L**) *Usp46^{1} Axin* (**G-L**) or *Wdr20^{33} Axin* (**J-L**) double null mutant wing disc clones (marked by absence of GFP, green) display ectopic expression of *nkd-lacZ* (magenta).

Scale bars: (A-C) and (D-L): 50 μM

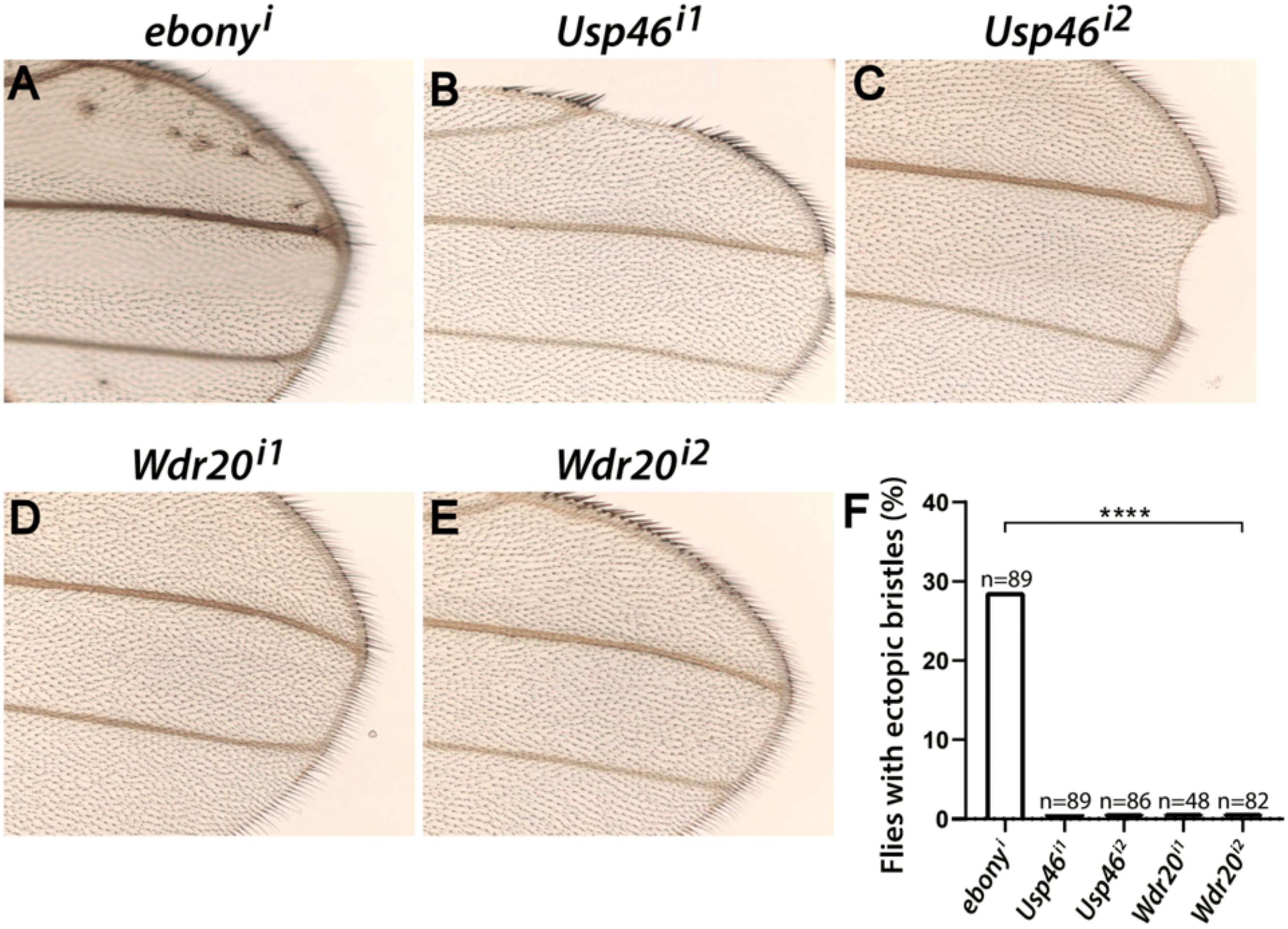

Figure S21. Usp46 complex components promote ectopic Wingless signaling induced by Arrow.
(**A**) Overexpression of *arrow* with the *C96-Gal4* driver results in ectopic sensory bristles in the adult wing blade caused by the ectopic activation of Wingless signaling, which is not rescued by RNAi-mediated depletion of the *ebony* control.

(**B-E**) Expression of two different *Usp46* RNAi (**B, C**) or *Wdr20* RNAi (**D, E**) constructs with the *C96-Gal4* driver reduced the ectopic bristles induced by *arrow* overexpression. (**B, C**) Notches at the wing margin, a hallmark of Wnt signaling reduction, were observed in some samples. Scale bar (A-E): 50 μM.

(**F**) Quantification of percent of flies with ectopic wing bristles induced by *arrow* overexpression coupled with RNAi-mediated knockdown of *Usp46*, *Wdr20,* or *ebony*. ****$p < 0.0001$ (0 for all genotypes, one-tailed t-test). Source data are provided in the Source Data file.

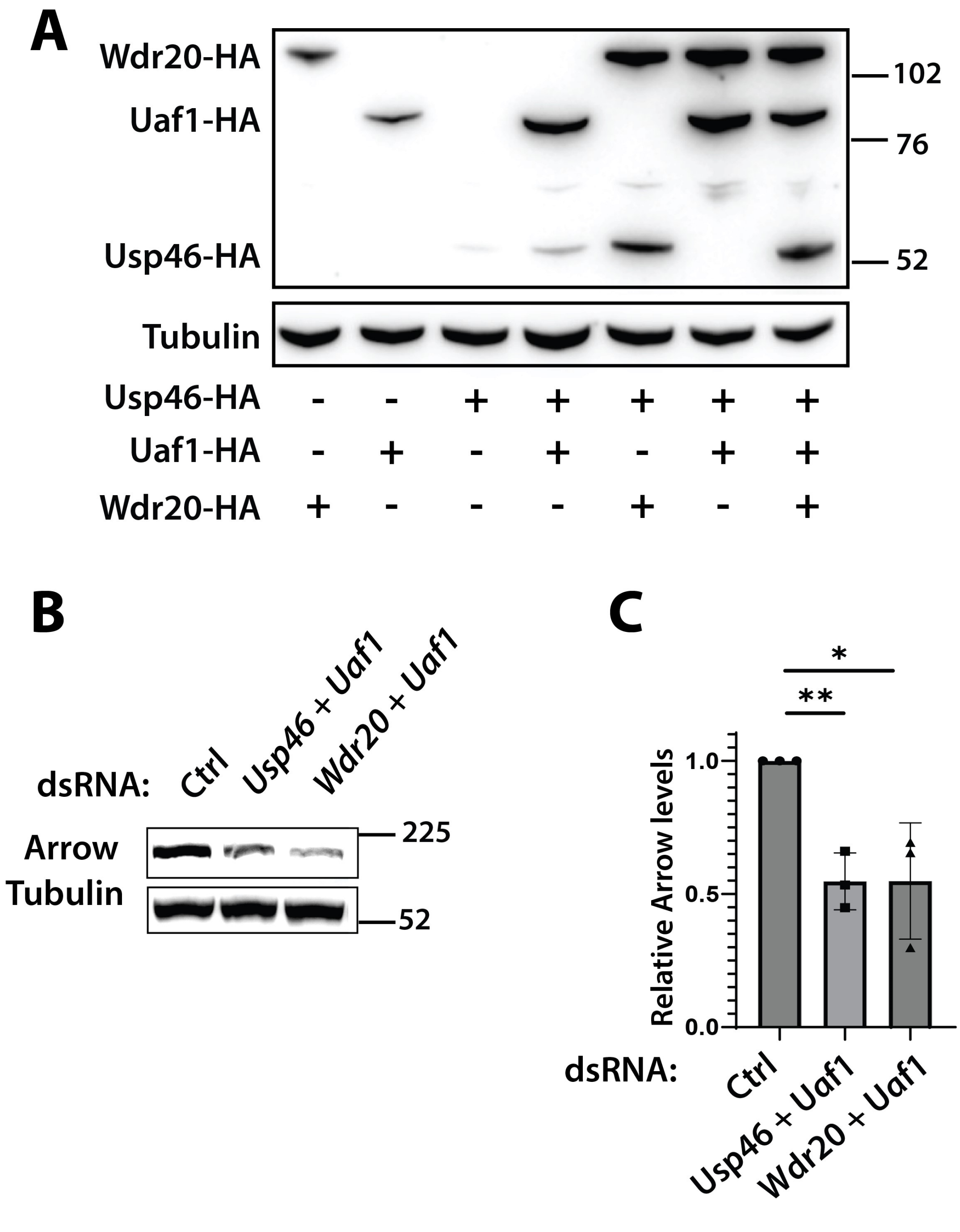

Figure S22. Usp46 complex stability.

(**A**) Auto-stabilization of the Usp46 complex. *Drosophila* S2R+ cells were transfected with individual or combinations of HA-tagged Usp46 complex components. Usp46 is stabilized upon co-transfection of Wdr20 and to a lesser degree upon co-transfection of Uaf1. Co-transfection of Usp46 stabilizes Uaf1 and Wdr20. Tubulin was used as a loading control.

(**B**) Knockdown of Usp46 complex components resulted in decreased Arrow levels. RNAi-mediated knockdown of the Usp46 complex decreases the steady-state levels of Arrow. S2R+ cells were treated with Ctrl or Usp46 complex dsRNAs as indicated, followed by immunoblotting with Arrow antibody. Tubulin was used as a loading control.

(**C**) Quantitation of Arrow levels normalized to tubulin**.** Mean and standard deviations are shown, n=3.*p = 0.0018 for *Usp46* + *Uaf1*,**p = 0.0231for *Wdr20* + *Uaf1* (two tailed t-test). Source data are provided in the Source Data file.

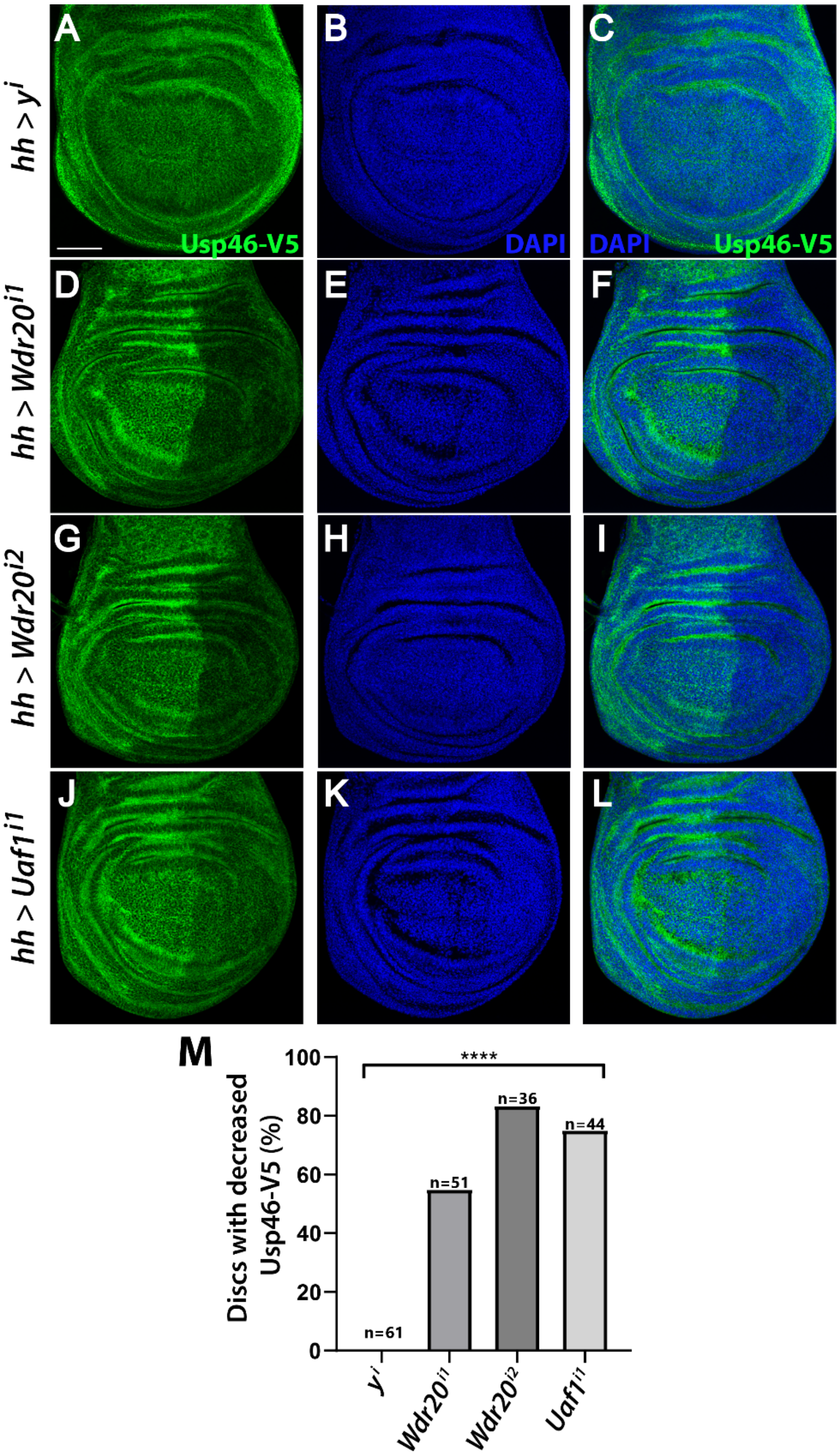

Figure S23. Wdr20 and Uaf1 stabilize Usp46 in larval wing imaginal discs.

(**A-C**) *hh-Gal4*-driven expression of a control RNAi construct targeting the *yellow (y)* gene. No reduction of Usp46-V5 (green) was observed.

(**D-I**) *hh-Gal4*-driven expression of two independent RNAi constructs targeting Wdr20 result in decreased Usp46-V5 in the posterior wing disc.

(**J-L**) *hh-Gal4*-driven expression of an RNAi construct targeting Uaf1 results in decreased Usp46-V5 in the posterior wing disc. Scale bar (A-L): 50 μM

(**M**) Quantification of percentage of wing discs with reduction of Usp46-V5 upon expression of the indicated RNAi construct. ****$p < 0.0001$ (0 for all genotypes, one-tailed t-test). Source data are provided in the Source Data file.

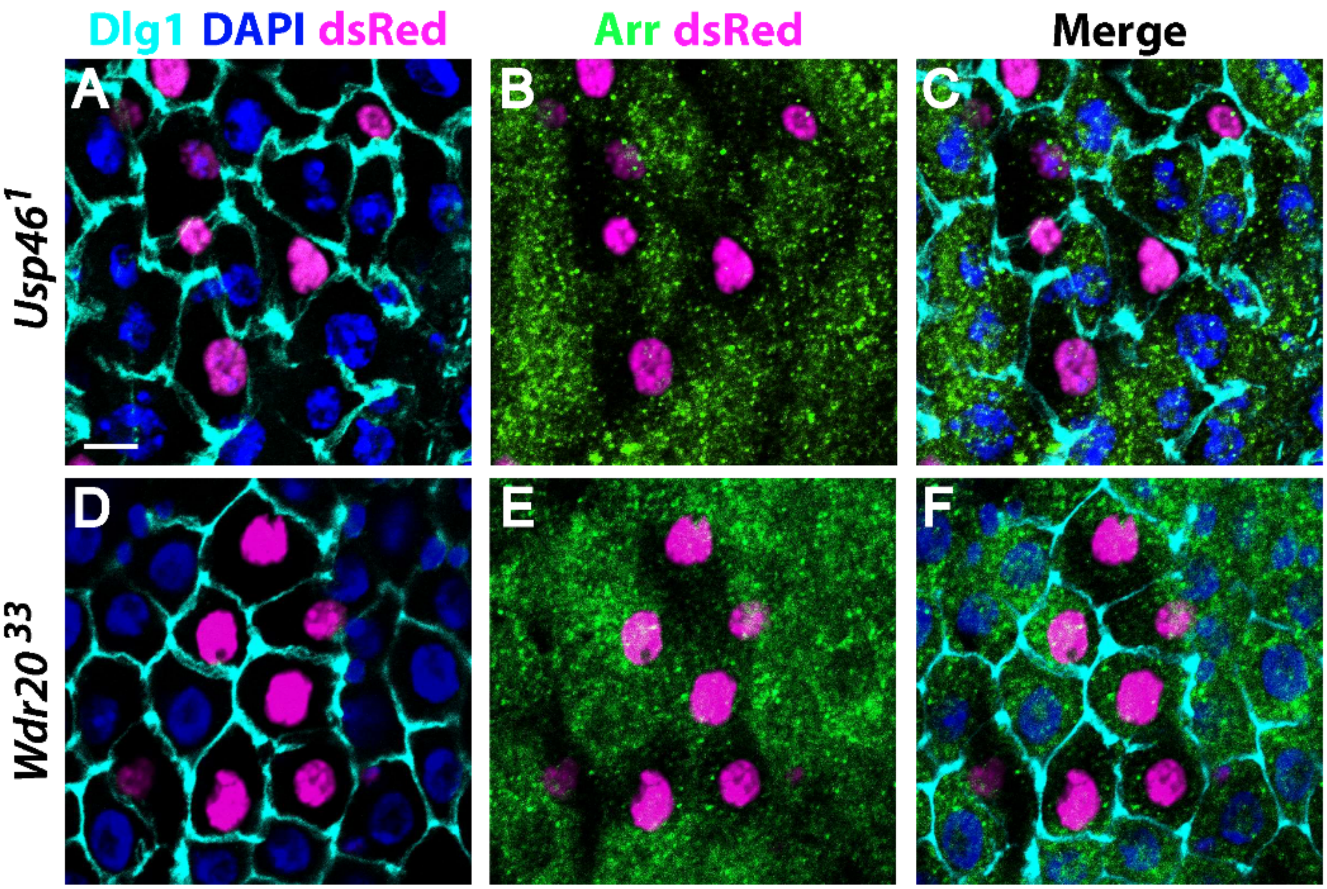

Figure S24. Usp46 and Wdr20 inactivation does not disrupt membrane integrity or cause cell death.
(**A-C**) *Usp46^{1}* null mutant clones (magenta) in the adult posterior midgut. Arrow (green) levels are decreased at the cell membrane and in the cytoplasm. Dlg1 (cyan) localization at the cell membrane and DAPI (blue) in the nuclei are normal, indicating normal apico-basal polarity.

(**D-F**) *Wdr20^{33}* null mutant clones (magenta) displayed wild-type staining of Dlg1 (cyan) and DAPI (blue), while showing reduction in Arrow levels (green).

Scale bar (A-F): 10 μM

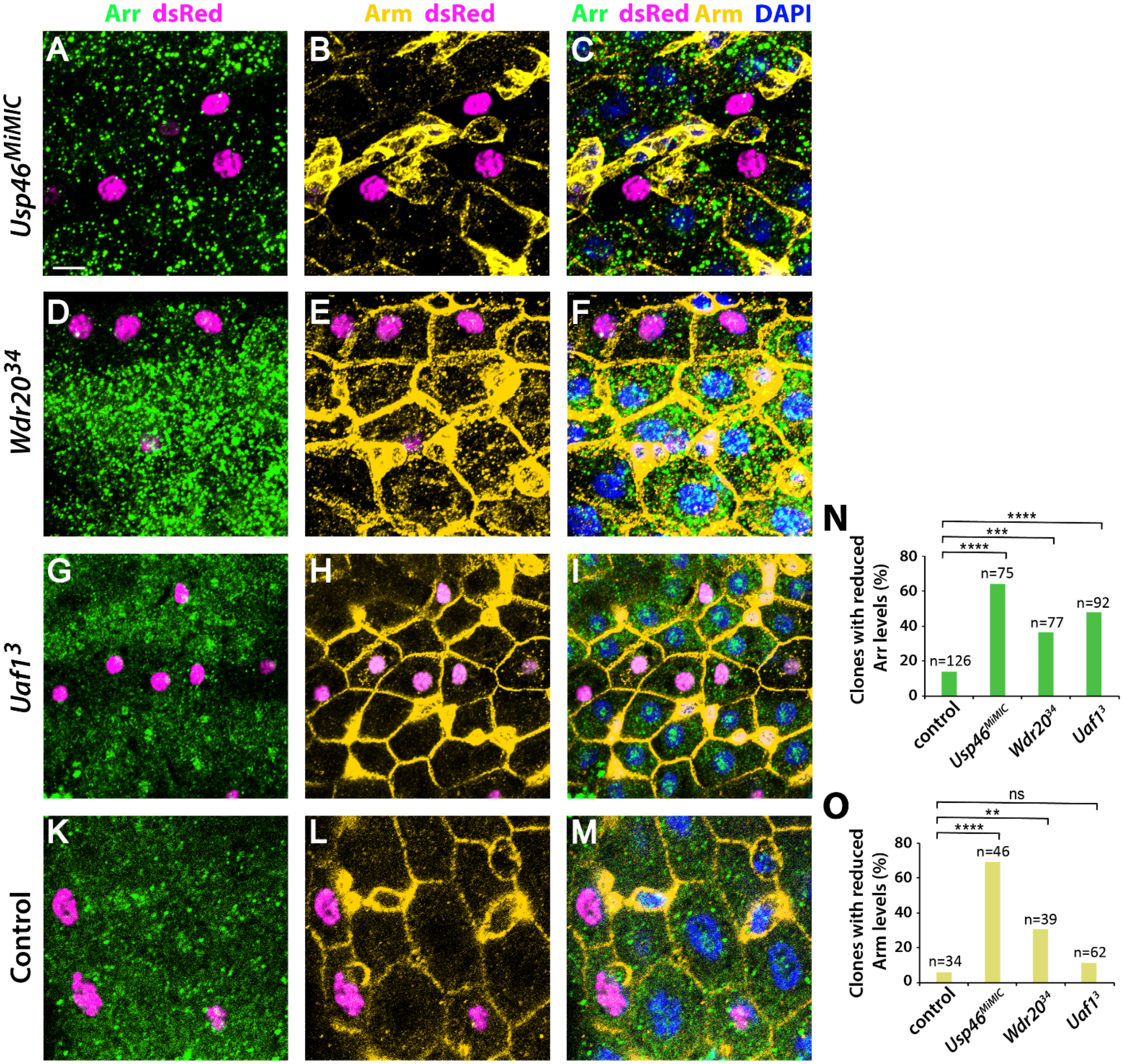

Figure S25. Independent null alleles confirm that Arrow levels are dependent on the Usp46 complex.
(**A-C**) *Usp46^{MiMIC}* null mutant clones (magenta) in the adult posterior midgut. Upon Usp46 inactivation, the levels of cell membrane-associated and cytoplasmic Arrow (green) are decreased cell-autonomously, as are the levels of cytoplasmic Arm (yellow). DAPI (blue) marks the nuclei.

(**D-F**) *Wdr20^{34}* null mutant clones (magenta) in the adult posterior midgut. Arrow (green), Arm and Prospero (yellow), DAPI (blue). Both Arrow (green) and cytoplasmic Arm (yellow) are decreased cell-autonomously upon Wdr20 inactivation. Some cells also display a non-autonomous decrease in membrane-associated Arm.

(**G-I**) *Uaf1^{3}* null mutant clones (magenta) in the adult posterior midgut. Upon Uaf1 inactivation, the levels of cell membrane-associated and cytoplasmic Arrow (green) are decreased cell-autonomously. DAPI (blue) marks cell nuclei.

(**K-M**) Control clones of wild-type cells (FRT82B, magenta) in the adult posterior midgut. Arrow (green) and DAPI (blue).

Scale bar (A-M): 10 μM

(**N, O**) Quantification is shown as percentage of clones of each genotype with decreased Arrow (N) or Arm (O) levels. N is the number of clones analyzed. ns = not significant (0.192955), $**p < 0.01$ (0.004368), $***p < 0.001$ (1.42E-4), ****p 0.0001 (0 for all genotypes, one-tailed t-test). Source data are provided as a Source Data file.,

Supplementary Table 1. Usp46 complex mutant alleles

Allele	Class	Aberration	Notes
Usp46^{1}	Null	Deletion of 862 bp (828-1689)	Wild-type translation to AA 125
Usp46^{21}	Null	Deletion of 5 bp (657-661)<br>3 bp deletion (826-828)	Wild-type translation to AA 68
Wdr20^{34}	Null	Deletion of 1852 bp (901-2752), with insertion of 10 bp	Wild-type translation to AA 132
Wdr20^{33}	Null	Deletion of 30 bp (565-594), with 2 bp insertion<br>Deletion of 1 bp (901)	Wild-type translation to AA 44
Wdr20^{13}	Null	Deletion of 10 bp (894-903)<br>Deletion of 3 bp (2258-2260)<br>Deletion of 1 bp (2749)	Wild-type translation to AA 132
Wdr20^{1A}	Null	Deletion of 1851 bp (903-2753)	Wild-type translation to AA 132
Uaf1^{4}	Null	Deletion of 4 bp (242-245)<br>Deletion of 48 bp (331-378)<br>Deletion of 1530 bp (385-1914)	Wild-type translation to AA 30
Uaf1^{3}	Null	Deletion of 1 bp (243)<br>Deletion of 1338 bp (576-1913), with insertion of 12 bp at deletion site	Wild-type translation to AA 31
Uaf1^{8}	Null	Deletion of 1339 bp (572-1910)	Wild-type translation to AA 31

*All positions with respect to the transcriptional start site

Supplementary Table 2. Usp46 complex gRNAs

Gene	gRNA	*PAM*
Usp46	GGAGACGCTGCTGTCGTGTC	***TGG***
	TCACATCAATGAGATCATAC	***TGG***
	AGCGCTTGAGATGCAGGGCC	***AGG***
	ATACACAAGTCTTCGGAGAC	***CGG***
Wdr20	GCAGGCGGTAGGTGCCCTCG	***CGG***
	ACTGTGGAGGCGCGTCTGGG	***CGG***
	CTCCCCGCACGTTGGTCCTC	***GGG***
	TGCGTGTGCTGGTCCCCTGA	***CGG***
Uaf1	GTGCGTCAACATGTCGTCCA	***CGG***
	TAGTACTTTGCTGTAATGGC	***AGG***
	ATGCGCAGAATGAAGCTGCG	***AGG***
	TAGAAGCCCTGCTAGAGTAC	***TGG***

Supplementary Table 3. gRNAs for V5 Tagging

V5 Conjugated Gene	gRNA	*PAM*
Usp46-V5	ACATACTGTTCTATCAGTCG	***CGG***
Wdr20-V5	TCCAGGTGACAAGTGCTGAG	***TGG***
	AACTAAATTACCGTCCCGCC	***GGG***
Uaf1-V5	ATCGTAGTTGAAATTCTGTT	***TGG***

Supplementary Table 4. gRNAs for tissue-specific CRISPR mutagenesis

Gene	Line #	gRNA 1	gRNA 2
Wdr20	Line 1	CTCCCCGCACGTTGGTCCTC ***GGG***	ATACTCGCGGCCCAACCGGG ***TGG***
	Line 2	TCGCCGGGAGCGTCGCCTAC ***GGG***	CCAAAGTGACCTGCCTAAAG ***TGG***
Uaf1	Line 3	CTTCGTGATACGGGACGCCG ***AGG***	CGTGGACCCGCTCACAATCA ***CGG***
	Line 4	GATTATGGCGTCGCGACCGG ***CGG***	CAGGCGCCCGTGCTGAGTCT ***GGG***
Usp46	Line 5	TCACATCAATGAGATCATAC ***TGG***	TGGGTGCACGAGATCTTTCA ***GGG***
	Line 6	CGCCCAAAAAGTTCATAACC ***AGG***	ACACCTATAATCTCAGGAAA ***CGG***
ebony	Line 8	GGCTGTGTGCATGCAGCCGT ***CGG***	GTTCTTCACCGGACGATAGT ***CGG***

Supplementary Table 5. Primers to amplify DNA templates for RNAi-mediated knockdown of *white, Usp46*, *Uaf1*, *Wdr20* and *arrow*

Gene	Sequence (5'→3')
white control	forward 5'-T7- ACCTGTGGACGCCAAGG-3'<br>reverse 5'-T7- AAAAGAAGTCGACGGCTTC-3'
Usp46-1	forward 5'-T7- GTGGACCAGAACACCTCGAT-3'<br>reverse 5'-T7- AGTGGATAACCACAGCGGTC-3'
Usp46-2	forward 5'-T7- GTTATCAGGAGGCGCAGAA-3'<br>reverse 5'-T7- AGTGGATAACCACAGCGGTC-3'
Uaf1-1	forward 5'-T7- AAAGGCTTCTGCATGTCCAC-3'<br>reverse 5'-T7- CGTGGATGGTTTGAACACAG-3'
Uaf1-2	forward 5'-T7- CTGTGTTCAAACCATCCACG-3'<br>reverse 5'-T7- AACAGCGAATGTCCGAGTTC-3'
Wdr20-1	forward 5'-T7-TGACCTGGTGACTGTGTGGT-3'<br>reverse 5'-T7- ACACCACCGTTGAGATAGGC-3'
Wdr20-2	forward 5'-T7- GGTCCTGGGTCTCTGTGGTA-3'<br>reverse 5-T7- ACACCACCGTTGAGATAGGC-3'
Arrow-4	forward 5'-T7- CGATATGGATGAGCCGTATGCGGT-3'<br>reverse 5'-T7- CCGTTGATGTCAATGGCATCGA-3'
Arrow-5	forward 5'-T7- GAGAACTGCGCCGATGGAGCT-3'<br>reverse 5'-T7- GCAGGAGGATTCCGGCACAATG-3'
