# Supplementary material for: The USP46 deubiquitylase complex increases Wingless/Wnt signaling strength by stabilizing Arrow/LRP6
Source: Nat Commun. 2023 Oct 5;14:6174. doi: 10.1038/s41467-023-41843-0 (PMC10556106; doi:10.1038/s41467-023-41843-0)
Supplement: Supplementary file 2 — Reporting Summary [file 41467_2023_41843_MOESM2_ESM.pdf]

nature portfolio

Corresponding author(s): Yashi Ahmed

Last updated by author(s): August 30, 2023

Reporting Summary

Nature Portfolio wishes to improve the reproducibility of the work that we publish. This form provides structure for consistency and transparency in reporting. For further information on Nature Portfolio policies, see our Editorial Policies and the Editorial Policy Checklist.

Statistics

For all statistical analyses, confirm that the following items are present in the figure legend, table legend, main text, or Methods section.

n/a	Confirmed	
☐	☒	The exact sample size (*n*) for each experimental group/condition, given as a discrete number and unit of measurement
☐	☒	A statement on whether measurements were taken from distinct samples or whether the same sample was measured repeatedly
☐	☒	The statistical test(s) used AND whether they are one- or two-sided<br>*Only common tests should be described solely by name; describe more complex techniques in the Methods section.*
☒	☐	A description of all covariates tested
☒	☐	A description of any assumptions or corrections, such as tests of normality and adjustment for multiple comparisons
☐	☒	A full description of the statistical parameters including central tendency (e.g. means) or other basic estimates (e.g. regression coefficient) AND variation (e.g. standard deviation) or associated estimates of uncertainty (e.g. confidence intervals)
☐	☒	For null hypothesis testing, the test statistic (e.g. *F*, *t*, *r*) with confidence intervals, effect sizes, degrees of freedom and *P* value noted<br>*Give P values as exact values whenever suitable.*
☒	☐	For Bayesian analysis, information on the choice of priors and Markov chain Monte Carlo settings
☒	☐	For hierarchical and complex designs, identification of the appropriate level for tests and full reporting of outcomes
☒	☐	Estimates of effect sizes (e.g. Cohen's *d*, Pearson's *r*), indicating how they were calculated

Our web collection on statistics for biologists contains articles on many of the points above.

Software and code

Policy information about availability of computer code

Data collection	Images of Drosophila larval wing discs and adult intestine were obtained using a Nikon A1Rsi laser scanning, Yokogawa CSU-W1 spinning disk SoRa or Nikon CSU-W1 spinning disk confocal microscopes and Photometrics Prime BSI sCMOS cameras and NIS Elements AR 5.30.01 software. Images of adult wings were obtained using a Leica MZFLIII stereomicroscope, Zeiss Axiocam 208 camera, and Nikon Zen 3.0 software.
Data analysis	Two-tailed student's t-test was performed using Prism GraphPad 9. One-tailed student's t-test was performed for using the GIGA P-value Calculator (https://www.gigacalculator.com/calculators/p-value-significance-calculator.php). Quantification of immunoblots was performed using ImageJ. Confocal images were processed with Adobe Photoshop and Illustrator software from Adobe Suite (2021-2023).

For manuscripts utilizing custom algorithms or software that are central to the research but not yet described in published literature, software must be made available to editors and reviewers. We strongly encourage code deposition in a community repository (e.g. GitHub). See the Nature Portfolio guidelines for submitting code & software for further information.

Data

Policy information about availability of data

All manuscripts must include a data availability statement. This statement should provide the following information, where applicable:

- Accession codes, unique identifiers, or web links for publicly available datasets
- A description of any restrictions on data availability
- For clinical datasets or third party data, please ensure that the statement adheres to our policy

All data supporting the findings of this study are available in the paper and the Supplementary information files. Raw data and original gel images are included in the Source Data file. All other relevant data are available from the authors upon reasonable request.

Field-specific reporting

Please select the one below that is the best fit for your research. If you are not sure, read the appropriate sections before making your selection.

☒ Life sciences ☐ Behavioural & social sciences ☐ Ecological, evolutionary & environmental sciences

For a reference copy of the document with all sections, see nature.com/documents/nr-reporting-summary-flat.pdf

Life sciences study design

All studies must disclose on these points even when the disclosure is negative.

Sample size	No sample size statistical calculations were performed. The n-numbers were determined based on standard practices in Drosophila studies and consistency of measurable differences between groups in preliminary experiments. We analyzed at least 10 samples per genotype.
Data exclusions	No data were excluded.
Replication	Each experiment in this study was successfully repeated at least three times with at least three biological replicates to verify the reproducibility of the findings.
Randomization	Flies used in this study for in vivo experiments were allocated into each group based on genotype and developmental time. Within each genotype, individuals were randomly chosen. For experiments using cell lines, no specific passage of cells was required and cells were randomly separated into groups.
Blinding	Researchers were not blinded during this study to ensure data were correctly collected from each treatment group. Sample preparation, data collection and image analysis were performed under the same conditions for all samples. To avoid bias, most of the data were analyzed in a quantifiable manner, followed by determination of statistical significance.

Reporting for specific materials, systems and methods

We require information from authors about some types of materials, experimental systems and methods used in many studies. Here, indicate whether each material, system or method listed is relevant to your study. If you are not sure if a list item applies to your research, read the appropriate section before selecting a response.

Materials & experimental systems

n/a	Involved in the study	
☐	☒	Antibodies
☐	☒	Eukaryotic cell lines
☒	☐	Palaeontology and archaeology
☐	☒	Animals and other organisms
☒	☐	Human research participants
☒	☐	Clinical data
☒	☐	Dual use research of concern

Methods

n/a	Involved in the study	
☒	☐	ChIP-seq
☒	☐	Flow cytometry
☒	☐	MRI-based neuroimaging

Antibodies

Antibodies used

chicken anti-GFP, Abcam ab13970 (1:10000)
rabbit anti-GFP, Thermo Fisher Scientific A-11122 (1:500)
rabbit anti-dsRed, Clontech/TaKaRa 632496 (1:500)
mouse anti-Discs Large (4F3), Developmental Studies Hybridoma Bank (DSHB) (1:50)
mouse anti-Armadillo (N2 7A1), DSHB (1:50)
mouse anti-β-gal, Promega Z378B (1:500)
rabbit anti-Arrow, gifted by S. Dinardo (see reference 87) (1:5000)
guinea-pig anti-Sens, gifted by H. Bellen (see reference 33) (1:2000)
mouse anti-V5 (SV5-Pk1), Thermo Fisher Scientific R960-25 (1:500)
rabbit anti-V5 (D3H8Q), Cell Signaling Technology, 13202 (1:1000)
mouse anti-Prospero (MR1A), DSHB (1:100)
mouse anti-Wg (4D4), DSHB (1:500)
mouse anti-En (4D9), DSHB (1:50)
rabbit anti-Phospho-Histone H3 (Ser10), Millipore 06-570 (1:1000)
mouse anti-ubiquitin (P4D1), Santa Cruz Biotechnology sc-8017 (1:500)
rabbit anti-FLAG, Proteintech 20543-1-AP (1:1000)
mouse anti-GAPDH, DSHB (1:500)
rat anti-HA (3F10), Roche (sold by Millipore Sigma) 11867423001 (1:2000)
mouse anti-HA (12CA5), Thermo Fisher Scientific MA1-12429

mouse anti-alpha-tubulin (DM1A), Sigma T6199 (1:10000)
guinea pig anti-Arrow, gifted from S. Eaton (see reference 88) (1:1000 (IB); 1:500 (IP))

Validation

We used all antibodies under manufacturers' recommended conditions and/or based on multiple previous publications. Certificates of analysis were provided on the supplier website.
chicken anti-GFP, Abcam ab13970: https://www.abcam.com/products/primary-antibodies/gfp-antibody-ab13970.html
rabbit anti-GFP, Thermo Fisher Scientific A-11122: Thermo Fisher Scientific, A-11122
https://www.thermofisher.com/antibody/product/GFP-Antibody-Polyclonal/A-11122
rabbit anti-dsRed, Clontech/TaKaRa 632496: https://www.takarabio.com/products/antibodies-and-elisa/fluorescent-protein-antibodies/red-fluorescent-protein-antibodies?catalog=632496
mouse anti-Discs Large (4F3), Developmental Studies Hybridoma Bank (DSHB):https://dshb.biology.uiowa.edu/4F3-anti-discs-large
mouse anti-Armadillo (N2 7A1), DSHB: https://dshb.biology.uiowa.edu/N2-7A1-Armadillo
mouse anti-β-gal, Promega Z378B (1:500): https://www.promega.com/products/protein-detection/primary-and-secondary-antibodies/anti_beta_galactosidase-mab/?catNum=Z3781
rabbit anti-Arrow, gifted by S. Dinardo (see reference 84)
guinea-pig anti-Sens, gifted by H. Bellen (see reference 33)
mouse anti-V5 (SV5-Pk1), Thermo Fisher Scientific R960-25: https://www.thermofisher.com/antibody/product/V5-Tag-Antibody-clone-SV5-Pk1-Monoclonal/R960-25
rabbit anti-V5 (D3H8Q), Cell Signaling Technology, 13202: https://www.cellsignal.com/products/primary-antibodies/v5-tag-d3h8q-rabbit-mab/13202?_requestid=957792
mouse anti-Prospero (MR1A), DSHB: https://dshb.biology.uiowa.edu/Prospero-MR1A
mouse anti-Wg (4D4), DSHB: https://dshb.biology.uiowa.edu/4D4
mouse anti-En (4D9), DSHB: https://dshb.biology.uiowa.edu/4D9-anti-engrailed-invected
rabbit anti-Phospho-Histone H3 (Ser10), Millipore 06-570: https://www.emdmillipore.com/US/en/product/Anti-phospho-Histone-H3-Ser10-Antibody-Mitosis-Marker,MM_NF-06-570?ReferrerURL=https%3A%2F%2Fwww.google.com%2F
mouse anti-ubiquitin (P4D1), Santa Cruz Biotechnology sc-8017: https://www.scbt.com/p/ubiquitin-antibody-p4d1
rabbit anti-FLAG, Proteintech 20543-1-AP: https://www.ptglab.com/products/Flag-Tag-Antibody-20543-1-AP.htm
mouse anti-GAPDH, https://dshb.biology.uiowa.edu/DSHB-hGAPDH-2G7
rat anti-HA (3F10), Roche (sold by Millipore Sigma) 11867423001: https://www.sigmaaldrich.com/US/en/product/roche/roahaha?gclid=CjwKCAjw5_GmBhBIEiwA5QSMxDQMe2v-o9uAukFBuGftm1EsGZqcrtyOSn-0l_g8vuBJL5x4OYaIChoCM-YQAvD_BwE&gclsrc=aw.ds
mouse anti-HA (12CA5), Thermo Fisher Scientific MA1-12429: https://www.thermofisher.com/antibody/product/HA-Tag-Antibody-clone-12CA5-Monoclonal/MA1-12429
mouse anti-alpha-tubulin (DM1A), Sigma T6199: https://www.sigmaaldrich.com/US/en/product/sigma/t6199
guinea pig anti-Arrow, gifted from S. Eaton (see reference 88)
goat anti-guinea pig IgG HRP, SouthernBiotech: https://www.southernbiotech.com/goat-anti-guinea-pig-igg-h-l-hrp-6090-05
goat anti-rat IgG HRP, BioRad, https://www.bio-rad-antibodies.com/polyclonal/rat-igg-antibody-5204-2504.html?f=hrp&_ga=2.32029565.2135686566.1637450992-1257771570.1634319661
goat anti-mouse IgG HRP: https://www.bio-rad-antibodies.com/polyclonal/mouse-igg-antibody-star207.html?f=hrp

Eukaryotic cell lines

Policy information about cell lines

Cell line source(s)

S2R+ cells used in this study were purchased from the Drosophila Genomics Resource Center. HEK293T used in this study was purchased from American Type Culture Collection

Authentication

The HEK293T cell line used in this study is routinely authenticated by ATCC using STR profiling.

Mycoplasma contamination

S2R+ cells are not subject to mycoplasma infection. HEK cells were tested for mycoplasma and tested negative.

Commonly misidentified lines
(See ICLAC register)

To our knowledge, there are no commonly misidentified cell lines that were used in the study.

Animals and other organisms

Policy information about studies involving animals; ARRIVE guidelines recommended for reporting animal research

Laboratory animals

The following Drosophila melanogaster wild-type, mutant and transgenic lines were used in this study:
nkd-lacZ (nls)61, esg-lacZ 62 esg-Gal4, UAS-GFP (esg>GFP) 62, GBE-Su(H)-lacZ 62, Delta-lacZ (Bloomington Drosophila Stock Center (BDSC #11651), mScar:T2A:sens, and fz3-GFP.

MARCM and hs>flp lines: MARCM 82B: y w hs-flp UAS-CD8::GFP; tub-Gal4 FRT82B tub-Gal80/TM6B 69 and yw hs-flp tub-Gal4 UAS-dsRed; FRT82B tub-Gal80/TM3, Ser 70. MARCM 42D: y w hs-flp; UAS-(nls)GFP tub-Gal4; FRT42D tub-Gal80 71 and yw hs-flp tub-Gal4 UAS-dsRed; FRT42D tub-Gal80/CyO, tub-Gal4/TM3 Sb. hs-flp; 82B ubi-GFP (B#52012).
RNAi lines and Gal4 drivers: The RNAi lines Usp46i1 (Vienna Drosophila Resource Center (VDRC) #27799), Usp46i2 (VDRC #100586), Wdr20i1 (VDRC #110609), Wdr20i2 (VDRC #42060), Uaf1i1 (VDRC #3810) and yi (VDRC #106068) were expressed in third instar larval wing discs using hh-Gal4 72 with UAS-dcr2 73 (BDSC #25757) or C96-Gal4 with UAS-dcr2, or ap-Gal4

Other Stocks : arr2, Axins044230. Usp46MiMIC (y1 w*; MiMIC Usp12-46MI02353 CG7029MI02353) (Bloomington Drosophila Stock Center (BDSC #35110), Wdr20 Df (w[1118]; Df(3R) BSC524/TM6C, Sb) (Bloomington Drosophila Stock Center #25025). Usp46 Df (w

[1118]; Df(3R) BSC618/TM6C, cu[1] Sb[1]) (Bloomington Drosophila Stock Center #25693), UAS-GFP::lacZ.nls (BDSC #6452) and UAS-Arrow-HA. Wild-type controls were FRT42D, FRT82B, and Canton S.

Both sexes of animals were used in larval wing disc experiments unless otherwise indicated. Females were used in adult intestine experiments. Individuals ranged in age from 4 to 24 days to investigate larval and adult phenotypes.

Wild animals	No wild animals were used in the study.
Field-collected samples	No field collected samples were used in the study.
Ethics oversight	The study did not require an ethical approval. The study used invertebrate laboratory animals (Drosophila melanogaster).

Note that full information on the approval of the study protocol must also be provided in the manuscript.
